# ﻿Checklist of the Cucurbitaceae of Madagascar

**DOI:** 10.3897/phytokeys.267.159218

**Published:** 2025-12-05

**Authors:** Minosoa B. Andriamiharisoa, Edgardo M. Ortiz, Tendro Radanielina, Hanno Schaefer

**Affiliations:** 1 Department of Plant Biology and Ecology, Campus universitaire d’Ambohitsaina, University of Antananarivo, BP 566, 101, Antananarivo, Madagascar University of Antananarivo Antananarivo Madagascar; 2 Plant Biodiversity, Department of Life Science Systems, Technical University of Munich (TUM), Emil-Ramann-Str. 2, D-85354 Freising, Germany Technical University of Munich Freising Germany

**Keywords:** Andohahela National Park, biodiversity hotspot, crop wild relatives, endemic species, *
Kedrostis
*, *
Peponium
*, Zombitsy-Vohibasia National Park

## Abstract

This checklist of Malagasy Cucurbitaceae comprises 26 genera with 77 species and five varieties, including five new species to be formally described when more complete ecological and genetic data are available. Of the 82 taxa, 65 are classified as indigenous, and of those, 52 species and 5 varieties are endemic (88% of the native taxa). Six exotic species have permanent populations outside cultivation (naturalised exotics). Another eleven exotics are cultivated and occasionally found escaped from cultivation (casuals). The checklist covers taxonomic information including type specimen(s), vernacular names, DNA sequence information, photos, global and national distribution data with status (endemic, non-endemic native, naturalised, or cultivated), plus ecological and habitat information. Our analysis of IUCN conservation status suggests that half of the native species (32) are threatened, mainly due to habitat destruction. For 12 taxa we lack information to suggest IUCN status but many of them might turn out to be threatened or even extinct. Nine species are absent from protected area, 21 were found only in a single reserve. The Andohahela National Park is home to the greatest Cucurbitaceae diversity with 13 species, followed by Zombitsy-Vohibasia with 12 species. The most species-rich genera are *Peponium* (up to ten endemic species), *Zehneria* (eight species, six of them endemic), *Ampelosycios* and *Xerosicyos* (each seven endemic species). Our study reveals overlooked diversity in a plant family of global importance and highlights the need for additional fieldwork and collection in several genera, including *Cayaponia*, *Cyclantheropsis, Peponium*, and *Kedrostis*.

## ﻿﻿Introduction

### ﻿﻿Geography

**Figure 1.**
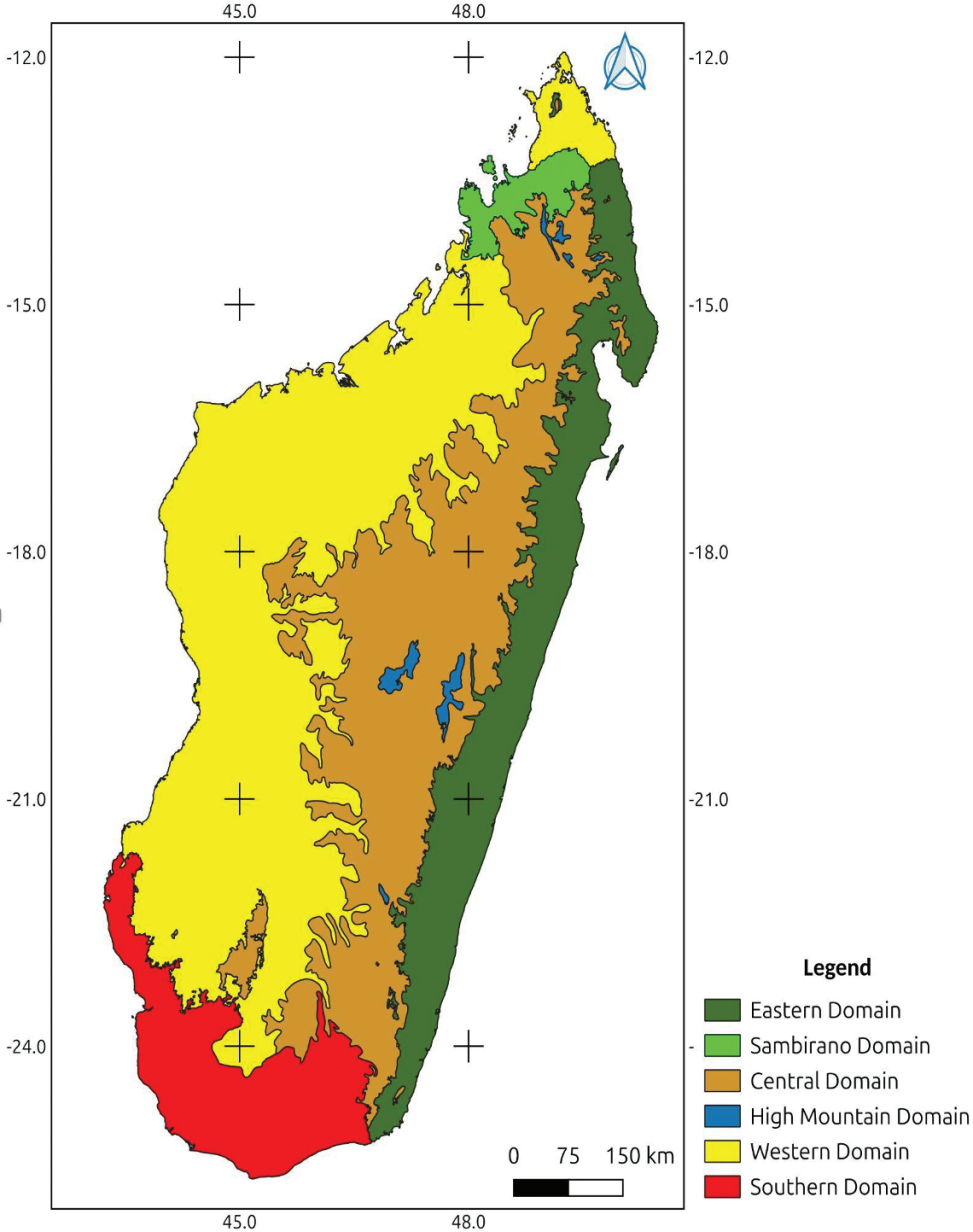
Phytogeographic domains of Madagascar based on Humbert (1955).

Madagascar is located between 12°–26°S and 43°–51°E in the southwestern part of the Indian Ocean, separated from the African continent by the Mozambique Channel. From north to south, the country stretches over 1,650 km and from west to east over 560 km, with a total surface area of 587,000 km^2^ (Goodman 2022). The western part accounts for up to two thirds of Madagascar’s total surface area, separated from the eastern part by the High Plateau with the highest mountain Tsaratanana, 2,876 m a.s.l. (Goodman 2022). The island can be divided into six phytogeographic domains: Central, Eastern, High Mountain, Sambirano, Southern, and Western domain (Fig. 1) (Humbert 1955), and six political provinces: Antananarivo, Toamasina, Antsiranana, Mahajanga, Fianarantsoa, and Toliary which are further divided into 24 regions (Faritra): Diana, Sava, Itasy, Analamanga, Vakinankaratra, Bongolava, Sofia, Ambatosoa, Boeny, Betsiboka, Melaky, Alaotra-Mangoro, Atsinanana, Analanjirofo, Amoron’i Mania, Vatovavy, Haute Matsiatra, Fitovinany, Atsimo-Atsinanana, Ihorombe, Menabe, Atsimo-Andrefana, Androy, and Anôsy. These regions are subdivided into 119 districts and 1,693 municipalities. In terms of demographics, according to the Institut National de la Statistique, in 2018 the total population of Madagascar was 25,674,196 (INSTAT 2020).

### ﻿﻿Geology of Madagascar

The 3-billion-year geological history of Madagascar includes continental separation, tectonic plate convergence, and subsequent continental collisions (Goodman 2022). More than two thirds of Madagascar’s bedrock is of Cambrian age, so it is roughly 540 million years (Myr) old (Collins and Windley 2002). The island of Madagascar broke away from Gondwana in the Late Cretaceous between 170–185 million years ago (Mya) (Goodman 2022) and today’s island of Madagascar is the result of the unification of several Gondwana blocks, the Antongil Block, Antananarivo Block, Tsaratanana Sheet, and Bemarivo Belt (Collins 2006). Several regions are today dominated by Neoproterozoic metasedimentary rocks, including the Molo, Betsimisaraka, Vohibory, and Androyen domains. The Antananarivo Block is underlain by gneiss, granitoid, and gabbros with an age of c. 2.5 billion years (Collins and Windley 2002). The Itremo sheet is mainly formed from Neoproterozoic metasedimentary rocks thrust over and imbricated with the Antananarivo Block. The Tsaratanana sheet lies on Middle to Late Archean gneiss with an age of 2.7–2.5 billion years (Cox et al. 1998). The Bemarivo Belt, located in the North, lies on east to west striking meta-sediments, granites, and gneiss overlapped by meta-volcanic rocks deformed by contraction (Thomas et al. 2009). The western part of Madagascar is dominated by meta-sediments, represented in the Northwest by the Mahajanga Basin and in the Southwest by the Morondava Basin (Collins and Windley 2002), which includes Upper Cretaceous volcanic rocks (Goodman 2022). Additionally, two types of volcanic formations are present in the central part of Madagascar: the massifs of Ankaratra and Itasy, which contain basaltic rocks from the Miocene epoch, 18 Myr old (Cucciniello et al. 2017).

### ﻿﻿Climate of Madagascar

Due to its wide latitude, mountain ranges, and influence of the sea, Madagascar has a very diverse climate. The warm winds from the Indian Ocean significantly increase humidity on the island’s eastern side. The dynamic interaction of subtropical anticyclones, trade winds, and monsoon flow also affects the climate (Nassor and Jury 1998). Broadly speaking, the climate of Madagascar can be categorized into four different climatic zones: the arid south, the temperate highlands, and the tropical eastern and western coastlines (Goodman 2022). The highlands experience a summer rainy season, the Southwest faces a leeward and dry climate, while the Northwest exhibits a tropical climate with heavy summer rain (Nassor and Jury 1998). The eastern part of the island has high humidity and 2,400 mm of precipitation per year, which can rise to over 6,000 mm in some places due to moisture laden winds from the Indian Ocean. In the South, it is much drier with annual precipitation of less than 600 mm, in the highlands about 1,600 mm are reached (Benstead and Goodman 2003). In the south and southwest, the lowest temperatures are between 20–25 °C in the coldest months of May–August. In comparison, average temperatures during the warmer months of November through mid-April range from 25–29 °C in the East to 27–28 °C in the South and Southwest. The central plateau, located at an elevation of around 1,650 m, typically has average temperatures between 12.5–20 °C throughout the hottest and coldest months and frost can occur at the highest elevations (Goodman 2022).

### ﻿﻿History of botanical exploration in Madagascar

The discovery of the Malagasy flora began in the 16^th^ and 17^th^ centuries, when European navigators first visited the island and brought back mainly medicinal plants and spices. Among them, Étienne de Flacourt (1607–1660), French governor of Madagascar for the French East India Company, is the most renowned. He arrived in Fort Dauphin (Tolagnaro) in 1648 and stayed until 1655. In 1658, he published the “Histoire de la grande île de Madagascar”, which includes the first description of Malagasy plant species. The French naturalist Philibert Commerson (1727–1773) came to collect for four months in 1770, while French aristocrat Louis-Marie Aubert du Petit Thouars (1758–1831) spent ten years (1792–1802) in exile in Madagascar after the French Revolution and collected about 2000 plant specimens. In 1822–1823, the Czech botanist Wenceslas Bojer (1795–1856) explored western Madagascar and visited together with the German botanist Carl Theodor Hilsenberg (1802-1824) the rainforests of the East, where Hilsenberg died of Malaria at the age of 22. French botanist Louis Hyacinthe Boivin (1808–1852) visited Madagascar between 1847–1852 and died of Malaria the day after his return to France.

The second era began in 1865 with the arrival of French explorer Alfred Grandidier (1836–1921) and his son, Guillaume Grandidier (1873–1957). Supported by the Museum d’Histoire Naturelle de Paris, they published the “Histoire physique, naturelle et politique de Madagascar” (1861) and “La bibliographie de Madagascar” (1906). The British missionary and botanist Richard Baron (1847–1907) arrived in 1872 and spent several decades collecting plants in the North and Centre. In his book “Compendium des plantes malgaches” (1901–1906), he listed 4,100 species in 970 genera.

The arrival of Henri Perrier de la Bâthie (1873–1958) in 1896 marked the beginning of the third great era of botanical discovery in Madagascar. He collected more than 20,000 herbarium specimens for the herbarium of the Museum d’Histoire Naturelle de Paris, and the National Herbarium of Madagascar (TAN) housed in the Tsimbazaza Botanical Garden in Antananarivo, which he had founded in 1925 and directed for over 40 years. In 1936, Henri Humbert (1887–1967) initiated the project “Flore de Madagascar (Plantes vasculaires)”, which later became the “Flore de Madagascar et des Comores”, published by the Museum d’Histoire Naturelle in Paris. In the same period, French botanists Raymond Decary (1891–1973), Henri Louis Poisson (1877–1963), Jacques D. Leandri (1903–1982), Jean Marie Bosser (1922–2013), and Bernard Descoings (1931–2018) contributed to the development of our knowledge of the Malagasy flora. These colleagues collected a significant part of the Malagasy Cucurbitaceae specimens available today, among them many later type specimens but they described very few of the species. The French botanist Monique Keraudren (1928–1981) wrote her PhD thesis on the Cucurbitaceae of Madagascar (Keraudren 1968) and she worked intensively with the available herbarium material in Paris and her own new collections and described 20 new taxa. Since Keraudren, hardly any research has been conducted on the Cucurbitaceae of Madagascar. The only exception was the German Werner Rauh (1913–2000), who was broadly interested in succulent plants of all kinds and did some work on the cucurbit genera *Seyrigia*, *Corallocarpus*, and *Xerosicyos* (Rauh 1995).

### ﻿﻿Vegetation

The first phytogeographical classification of Madagascar was established by Richard Baron who divided the country into three phytogeographical zones: East, Centre, and West (Baron 1889). Henri Perrier de la Bâthie (1921) added two types of formation in relation to southeast trade wind exposure: “Flore du vent” (east) and “Flore sous le vent (west). He divided the “Flore du vent” into three distinct zones: East, Central and Sambirano, and the “Flore sous le vent” into a western and a southern zone. Madagascar is marked by an aridity that increases from east to west due to the drying out of the trade wind after its passage over the eastern flank and central part of the island. This so-called “Foehn Effect” causes the “Flore sous le vent” of the West and South with distinct xerophytes and forms of adaptation to drought. The East of Madagascar is exposed to the trade wind all year. In the central part, the remaining wind, charged with moisture, causes the formation of fog and drizzle, which reduces the aridity of the dry season. In consequence, the vegetation varies according to temperature and rainfall in the eastern part of the island, while it varies according to different soil types in the western part. In 1955, Henri Humbert integrated both vegetation and plant distribution data into a new proposal for the phytogeographic division of Madagascar (Humbert 1955), which would later become a more refined classification in his “Carte de la végétation de Madagascar”, which comprised three scales: “région”, “domaine”, and “secteur”. He divided the island into two zones: East and West (Humbert and Darne 1965), which differ mainly in their humidity levels, underlining the edaphic variations within the different districts. The Eastern zone comprises three domains: East, Sambirano, and Center plus High Mountains; the Western zone is made up of two domains: West and South. The original classification of Humbert (1955) has been refined by Faramalala et al. (1995), Du Puy and Moat (1996), and Gautier et al. (2018) taking anthropogenic transformation into account.

### ﻿﻿Objectives

With this checklist, we want to provide an update on taxonomy and distribution of the Cucurbitaceae of Madagascar and add, wherever possible, also ecological information and evaluations of the threat level. While the information for most taxa is still incomplete, we see this as a first important step toward a modern flora treatment of the Cucurbitaceae of Madagascar. Several of the Malagasy cucurbit species are close relatives of important crops (crop wild relatives, CWR) and often evolved unique adaptations to drought (succulent organs) which makes their study also relevant for breeding of new crop varieties in a world with changing climates.

## ﻿﻿Material and methods

The basis for our work is the last comprehensive Cucurbitaceae treatment of the “Flore de Madagascar et des Comores” (Keraudren 1966), which listed 27 genera with 70 species. To evaluate and update Keraudren’s treatment, we studied and collected Cucurbitaceae in ten protected areas of Madagascar from December 2021 to January 2025: Andasibe-Mantadia National Park (NP), Andohahela NP, Ankarafantsika NP, Ankarana Special Reserve, Ankodida, Behara-Tranomaro, Berenty private reserve, Ranomafana NP, Montagne d’Ambre NP, Montagne des Français, and Zombitsy-Vohibasia NP. Herbarium voucher specimens have been deposited at the herbaria of Department of Botany, University of Antananarivo, DBEV, and Technical University of Munich, TUM. In addition, we examined several thousand herbarium specimens of the following herbaria: A, BR, CAS, CNARP, G, K, L, MO, P, S, TAN, US, W, and WAG, herbarium acronyms follow Index herbarorium (sweetgum.nybg.org/science/ih/). We also examined all Cucurbitaceae records on iNaturalist (https://www.inaturalist.org) and Tropicos (legacy.tropicos.org/Project/Madagascar), the latter including records from the Missouri Botanical Garden Team (MBG), Tsimbazaza Botanical and Zoological Park Team (PBZT-TAN), and Kew-Madagascar Conservation Centre Team (KMCC). Finally, we assembled a database of geographic occurrence records for all Malagasy Cucurbitaceae from the Global Biodiversity Facility (GBIF.org 2022, 2023). Relevant protologues and diagnoses were studied on Biodiversity Heritage Library (BHL, https://www.biodiversitylibrary.org/), JSTOR Global Plants (http://plants.jstor.org), and Botanicus (https://www.botanicus.org/). The vegetation classification used in this study follows the typology of Gautier et al. (2018). Information on vernacular names was compiled from herbarium labels.

To suggest the IUCN conservation status for all native species, we determined the Extent of Occurrence (EOO, criterion B1) and Area of Occupancy (AOO, criterion B2) (IUCN 2012) to identify species or populations with a restricted distribution that are either fragmented or occupy few locations. Population declines or extreme fluctuations in distribution areas could not be considered due to the lack of temporal information. To assess AOO, grid cells of 2 × 2 km were used as recommended by IUCN (IUCN Standards and Petitions Committee 2022) and EOO computing was based on the minimum convex polygon (MCP) from the REDLISTR package (Lee et al. 2019) using R version 4.4.2 (R Core Team 2024). A species is classified as Data Deficient (DD), when there are fewer than two occurrence data points. To obtain distribution maps, occurrence data was plotted on maps showing the phytogeographic domains (Humbert 1955) and protected areas of Madagascar (https://protectedareas.mg/). From these maps, we inferred diversity hotspots at both genus and species levels and tried to identify key areas for Cucurbitaceae conservation in Madagascar.

## ﻿﻿Checklist of the species in alphabetical order

### ﻿﻿Accepted species found outside cultivation

A total of 66 species of Cucurbitaceae have been found in the wild in Madagascar. Of those, 60 species (plus five varieties) are classified as indigenous species (Table 1) and most of them are endemic to Madagascar (52 species plus five varieties). An additional six species are classified as naturalised exotics, which have been introduced by human activities (Table 2), some of them with invasive tendencies, others with localised but well-established populations in the wild.

**Table 1.** Native Cucurbitaceae of Madagascar with status and distribution.

No.	Scientific name	Status	Phytogeographic domain
1.	*Ampelosycios bosseri* (Keraudren) H. Schaef. & S.S. Renner	Endemic	Western
2.	*Ampelosycios humblotii* (Cogn.) Jum. & H. Perrier	Endemic	Sambirano, Western, Central, Eastern
3.	*Ampelosycios leandrii* (Keraudren) H. Schaef. & S.S. Renner	Endemic	Western
4.	*Ampelosycios meridionalis* Keraudren	Endemic	Western, Southern
5.	*Ampelosycios scandens* Thouars	Endemic	Central
6.	*Ampelosycios* sp. nov. (Ankarana)	Endemic	Western
7.	*Ampelosycios* sp. nov. (Montagne des Français)	Endemic	Western
8.	*Blastania lucorum* (Keraudren) H. Schaef.	Endemic	Western, Southern
9.	Cayaponia africana (Hook.f.) Exell var. madagascariensis Keraudren	Endemic	Western
10.	*Citrullus mucosospermus* (Fursa) Fursa	probably native but non-endemic, CWR	Western, Central, Eastern, Southern
11.	*Corallocarpus grevei* (Keraudren) Keraudren	Endemic	High Mountain, Western, Southern
12.	*Corallocarpus perrieri* (Keraudren) Keraudren	Endemic	Southern
13.	*Corallocarpus poissonii* Cogn.	Native non-endemic	Western
14.	*Cucumis sacleuxii* Paill. & Bois	Native but not endemic, CWR	Western, Central, Eastern
15.	*Cucumis* sp. nov. (aff. cinereus)	Endemic, CWR	Western
16.	*Cucumis* sp. nov. (aff. hirsutus)	Endemic, CWR	High Mountain, Central
17.	*Cucumis subsericeus* Hook.f.	Native non-endemic, CWR	Sambirano, High Mountain, Central
18.	*Cyclantheropsis madagascariensis* Keraudren	Endemic	Western, Southern
19.	*Gerrardanthus lobatus* (Cogn.) C. Jeffrey	Native non-endemic	Western
20.	*Kedrostis cogniauxii* Keraudren	Endemic	Sambirano
21.	*Kedrostis dissecta* Keraudren	Endemic	Western, Central
22.	*Kedrostis elongata* Keraudren	Endemic	Sambirano, Western, Central, Eastern
23.	*Kedrostis lanuginosa* Keraudren	Endemic	Western, Eastern
24.	*Kedrostis laxa* Keraudren	Endemic	Western
25.	*Kedrostis perrieri* Keraudren	Endemic	Western
26.	*Lemurosicyos variegata* (Cogn.) Keraudren	Endemic	Western, Southern
27.	*Muellerargia jeffreyana* Keraudren	Endemic, CWR	Western, Central
28.	*Peponium betsiliense* Keraudren	Endemic	Western, Central
29.	*Peponium boivinii* (Cogn.) Engl.	Endemic	Sambirano, Western
30.	*Peponium grandidieri* Keraudren	Endemic	Sambirano, Western, Southern
31a.	Peponium hirtellum Keraudren var. hirtellum Keraudren	Endemic	Southern
31b.	Peponium hirtellum Keraudren var. longiracemosum Keraudren	Endemic	Central, Eastern, Southern
32.	*Peponium humbertii* Keraudren	Endemic	High Mountain, Western, Central
33.	*Peponium laceratum* Keraudren	Endemic	Central
34a.	Peponium perrieri Keraudren var. perrieri	Endemic	Western, Central
34b.	Peponium perrieri Keraudren var. glabrescens Keraudren	Endemic	Western
35.	*Peponium poissonii* Keraudren	Endemic	Western, Eastern, Southern
36.	*Peponium racemosum* Keraudren	Endemic	Western, Central
37a.	Peponium seyrigii Keraudren var. seyrigii	Endemic	Southern
37b.	Peponium seyrigii Keraudren var. linearilobum Keraudren	Endemic	Western
38.	*Raphidiocystis brachypoda* Baker	Endemic	High Mountain, Western, Central
39.	*Seyrigia bosseri* Keraudren	Endemic	Central, Southern
40.	*Seyrigia gracilis* Keraudren	Endemic	Western, Southern
41.	*Seyrigia humbertii* Keraudren	Endemic	Southern
42.	*Seyrigia marnieri* Keraudren	Endemic	Western, Southern
43.	*Seyrigia multiflora* Keraudren	Endemic	Central, Southern
44.	*Seyrigia napifera* Rauh	Endemic	Central, Southern
45.	*Trochomeriopsis diversifolia* Cogn.	Endemic	Sambirano, Western, Central, Southern
46.	*Xerosicyos danguyi* Humbert	Endemic	Western, Eastern, Southern
47.	*Xerosicyos decaryi* Guillaumin & Keraudren	Endemic	Western, Southern
48.	*Xerosicyos hirtellus* (Humbert) H. Schaef. & S.S. Renner	Endemic	Southern
49.	*Xerosicyos perrieri* Humbert	Endemic	Western, Central, Southern
50.	*Xerosicyos pubescens* Keraudren	Endemic	Southern
51.	*Xerosicyos* sp. nov. (North Madagascar)	Endemic	Western
52.	*Xerosicyos tripartitus* (Humbert) H. Schaef. & S.S. Renner	Endemic	Central, Southern
53.	*Zehneria emirnensis* (Baker) Keraudren	Native non-endemic	High Mountain, Central, Eastern, Southern
54.	*Zehneria madagascariensis* Keraudren	Endemic	High Mountain, Central
55.	*Zehneria martinez-crovettoi* Keraudren	Endemic	Central, Southern
56.	*Zehneria peneyana* (Naudin) Schweinf. & Asch.	Native non-endemic	Western
57a.	Zehneria perrieri Keraudren var. perrieri	Endemic	Central, Western
57b.	Zehneria perrieri Keraudren var. parvula Keraudren	Endemic	Central
57c.	Zehneria perrieri Keraudren var. tsaratananensis Keraudren	Endemic	High Mountain
58.	*Zehneria polycarpa* (Cogn.) Keraudren	Endemic	Sambirano, Western, Central, Eastern, Southern
59.	*Zehneria rutenbergiana* (Cogn.) Keraudren	Endemic	Central, Eastern
60.	*Zehneria tridactyla* (Hook.f.) R. Fern. & A. Fern.	Native non-endemic	Sambirano, Western, Central, Eastern

**Table 2.** Naturalised Cucurbitaceae of Madagascar with status and distribution.

No.	Scientific name	Status	Distribution
1.	*Coccinia grandis* (L.) Voigt	Naturalised	Eastern, Southern
2.	*Cucumis anguria* L.	Naturalised	Southern
3.	*Cucumis dipsaceus* Ehrenb. ex Spach	Naturalised	Southern
4.	*Cucumis melo* L.	Naturalised	Sambirano, Western, Central, Southern
5.	*Diplocyclos palmatus* (L.) C.Jeffrey	Naturalised	Western, Central, Eastern
6.	*Momordica charantia* L.	Naturalised	All

***Ampelosycios* Thouars, Hist. Vég. Isles Austral. Afriq.: 68 (1808).**

**Generic type.***Ampelosycios scandens* Thouars.

**Worldwide Distribution.** endemic to Madagascar (Schaefer 2020).

***Ampelosycios bosseri* (Keraudren) H. Schaef. & S. S. Renner, Taxon 60: 133 (2011)**

Fig. 2

**Basionym.***Odosicyos bosseri* Keraudren, *Bull. Soc. Bot. France, Lett. Bot.* 127(5): 518. 1980 [1981].

**Vernacular name.** Belataka antaninolo (Masikoro).

**Holotype.** Madagascar • Forêt de Lambomakandro entre Ranohira et Sakaraha, forêt sèche, Jan. 1962, *M. Keraudren 1312* P (P00464856!).

**Isotypes.** P (P00464854!, P00464855!).

**GenBank information.** DNA sequences on NCBI from Kocyan et al. (2007): DQ535832, DQ536710, DQ536773, DQ648183 and Filipowicz and Renner (2010): HM008593.

**Distribution.** Endemic to Madagascar, where it is found only in the surroundings of Sakaraha (Fig. 3).

Western Domain: Atsimo-Andrefana region, from Ranohira to Sakaraha, Lambomakandro, *M. Keraudren 485, 1312* (P), *J. Leandri 3891* (P), *M.M. Debray 1914* (P), *S. Hofstätter 73242* (P); Ambiamena, Zombitsy NP, *H. Schaefer et al. 88, 90* (TUM).

**Occurrence in Protected Areas.** Zombitsy-Vohibasia NP.

**Ecology and habitat.** Remnants of the western moist semi-deciduous forest on sandy soil at an altitude of 500 m asl.; flowering Nov.–Oct. (Keraudren-Aymonin 1980, 1982a).

**Uses.** The species is occasionally grown as an ornamental caudex plant in the Northern Hemisphere, and wild-collected material of dubious origin is sold online.

**Taxonomy.** According to Schaefer et al. (2009), *A. bosseri* is sister to *A. leandrii*.

**IUCN.** The AOO of the species is equal to 7 km^2^, which classifies the species as Critically Endangered (CR). In addition to strong pressures from forest fires and illegal grazing, its habitat is threatened by mining activities (Gautier et al. 2018).

***Ampelosycios humblotii* (Cogn.) Jum. & H. Perrier, Ann. Fac. Sci. Marseille 23: 29 (1915)**

Fig. 4

**Basionym.***Delognaea humblotii* Cogn., *Bull. Mens. Soc. Linn.* Paris 1: 425 (1884).

**Vernacular name.** Vahitakaboka (Betsileo), Vahanonoka (Betsimisaraka), Voanono (Betsimisaraka).

**Holotype.** Madagascar • 28 Oct. 1883, *Humblot 203* P (P00135525!).

**Isotypes.** P (P00135526!), K (K000310405!), MO (MO2945897!).

**GenBank information.** DNA sequences on NCBI published by Kocyan et al. (2007) and Bellot et al. (2020): DQ501254, DQ501261, DQ521608, NC046869, MN542396.

**Distribution.** Endemic to Madagascar where it is found mainly in the East (Fig. 5).

Sambirano Domain: Diana region, Lokobe, *L.H. Boivin 1851-3* (P).

Central Domain: Ihorombe region, Iakora, Begogo, Bekora, Sahalava Forest, *Andrianjafy 776* (MO); Ivohibe, *P. De Block, J. Tosh & F. Rakotonasolo 320, 321* (BR).

Eastern Domain: Vatovavy region, Ranomafana NP, *R. Rakoto 46* (WAG), *H. Schaefer et al. 110, 121, 126* (DBEV, TUM); Ambatosoa region, Maroantsetra, Ambinanitelo, Marovovonana, *Antilahimena 2881* (MO), *G. Cours 4075* (P); Alaotra-Mangoro region, Analamazaotra, Andasibe, *M. Keraudren 1752* (P), *G.G. Aymonin 25373* (P), *M. Keraudren-Aymonin 25366, 25373* (P), *H. Perrier de la Bâthie 9017* (P), *J. Bosser 15952* (P); Sava region, Masoala Peninsula *Vasey 117* (WAG), *R. Behasy 117* (P).

**Occurrence in Protected Areas.** Lokobe NP, Masoala NP, Mantadia NP, Ranomafana NP, Tsingy de Bemaraha NP, and Ankeniheny-Zahamena Corridor Natural Resource Reserve.

**Ecology and habitat.** Lowland and medium altitude moist evergreen forest on alluvial deposits between 200–1,700 m asl.

**Uses.** Locals eat the roasted seeds (Keraudren 1968).

**Taxonomy.** According to Schaefer et al. (2009), *A. humblotii* is sister to *A. scandens*.

**IUCN.** Based on the AOO assessment the species is Critically Endangered (CR), but the EOO indicates a status of Least Concern (LC), which might be more appropriate.

***Ampelosycios leandrii* (Keraudren) H. Schaef. & S.S. Renner, Taxon 60: 133 (2011)**

Fig. 6

**Basionym**. *Tricyclandra leandrii* Keraudren, *Bull. Soc. Bot. France* 112: 327 (1966).

**Holotype.** Madagascar • Kamakama sur le plateau d’Ankara, Jan. 1900, *H. Perrier de la Bâthie 1016* P (P00462257!).

**Isotype.** P (P00462258!).

**GenBank information.** DNA sequences on NCBI by Kocyan et al. (2007): DQ491015, DQ491023, DQ501259, DQ501272.

**Distribution.** Endemic to Madagascar where it has been found only in the West (Fig. 7).

Western Domain: Boeny Region, Ankarafantsika, *H. Perrier de la Bâthie 6787, 1016* (P), *P.B. Phillipson 1922* (P); Melaky Region, Bekopaka, *P. Morat 894* (P); Ampijoroa forest station, forest around banks of the lake, Tsingy de Bemaraha, *C.C.H. Jongkind 3532* (WAG), *J. Léandri 2761* (P); Maintirano, Tsingy of Beanka, *Razafimandimby 6139* (P).

**Occurrence in Protected Areas.** Ankarafantsika NP, Tsingy de Bemaraha NP.

According to label information of *Jardin Botanique Tananarive 1447* (P), *A. leandrii* used to be in cultivation at the botanical garden of Tsimbazaza, Antananarivo, but we were unable to find it there in 2023.

**Ecology and habitat.** Moist semi-deciduous and dry deciduous forest, on sandy soils, and on limestone rocks at altitudes up to 800 m asl., characterised by moderate rainfall (400–1,400 mm/year), pronounced dry season (4–9 months), high temperatures, and low annual and daily temperature ranges (Gautier et al. 2018).

**Uses.** Cultivated as a caudex ornamental in the Northern Hemisphere. Wild-collected material (mainly seeds) of dubious origin is sold online.

**Taxonomy.***Ampelosycios leandrii* is sister to *A. bosseri* (Schaefer et al. 2009).

**IUCN.** We suggest Critically Endangered (CR) based on IUCN criterion B2.

***Ampelosycios meridionalis* Keraudren, Bull. Soc. Bot. France 112: 71 (1965).**

**Holotype.** Madagascar • Toliara, entre Tranoroa et Beloha, bush dégradé sur sable, Mar. 1964, *J. Bosser 19154* P (P00464871!), our Fig. 8.

**GenBank information.** DNA sequences are available from the P herbarium material and will be released with our revision of the genus (Andriamiharisoa et al., in prep.).

**Distribution.** Endemic to Madagascar, where it has been found only in three places in the Southeast (Fig. 9).

Western Domain: Ihorombe region, Horombe, *H. Poisson 701* (P).

Southern Domain: Ambovombe, *Decary 8590* (P); between Tranoroa and Beloha, *J. Bosser 19154* (P).

**Occurrence in Protected Areas.** Not known from protected areas.

**Ecology and habitat.** Dry and spiny thickets and moist semi-deciduous forest, between 0–500 m asl., characterised by low and irregular rainfall (< 600 mm/year), dry season of over 10 months, high to very high temperatures, significant annual, and high daily temperature ranges (Gautier et al. 2018).

**Taxonomy.** Close relative to *A. scandens* and *A. humblotii* (unpubl. data).

**IUCN.** We suggest Data Deficient (DD) since the last collection dates to the 1960s, and the species might have become extinct. A search expedition by the authors in the species’ former range in December 2024/January 2025 could not trace any plants and only few remnants of native vegetation within huge areas of sisal plantations.

**Figures 2–7.**
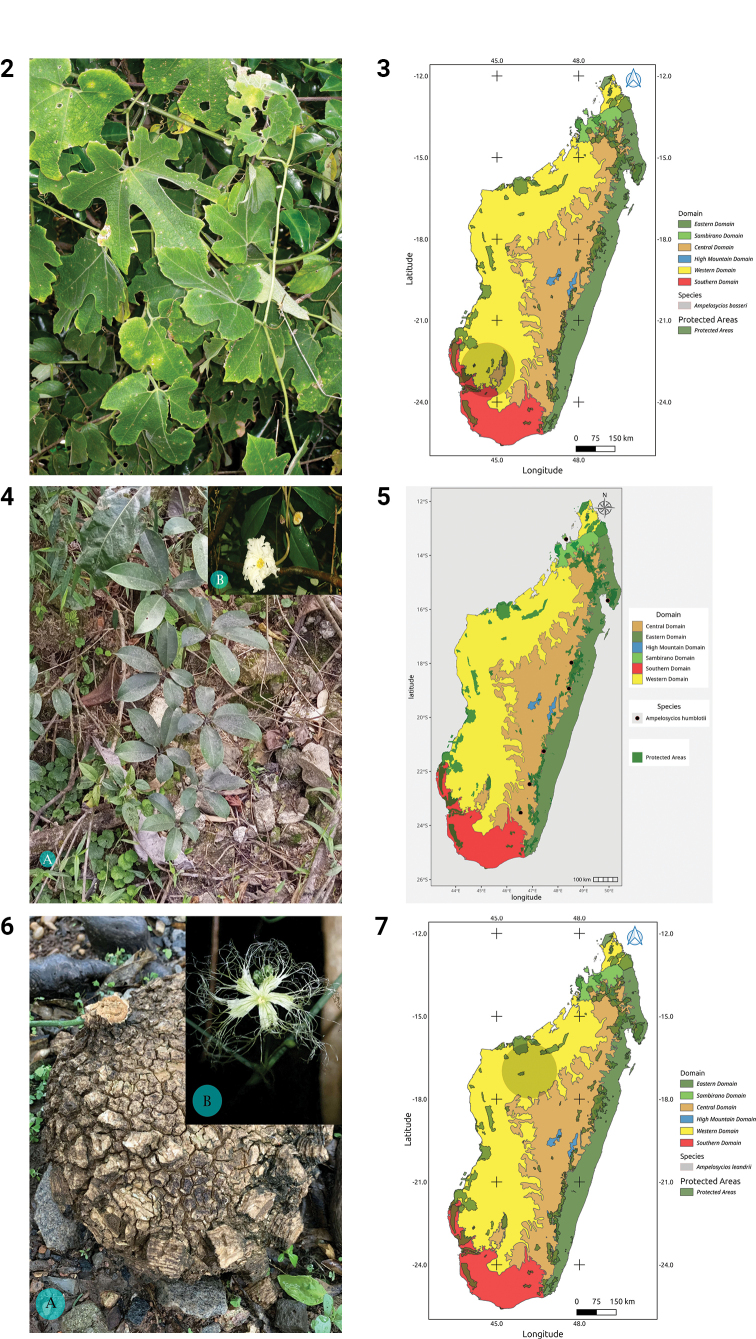
**2.***Ampelosycios bosseri*, habit (photo by HS, Zombitsy NP, March 2013). **3.** Distribution of *Ampelosycios bosseri*. **4.***Ampelosycios humblotii*. **A.** Habit; **B.** Flower (photos by MBA, Ranomafana NP., October 2022 (**A**) and by H. Schaefer, Ranomafana NP, March 2013 (**B**)). **5.** Distribution of *Ampelosycios humblotii*. **6.***Ampelosycios leandrii*. **A.** Tuber from a plant cultivated in Antananarivo; **B.** Male flower blooming during the night (photos by HS, Antananarivo and Ankarafantsika NP, December 2021). **7.** Distribution of *Ampelosycios leandrii*.

***Ampelosycios scandens* Thouars, Hist. Vég. Isles Austral. Afriq.: 68 (1808)**

Fig. 10

**Holotype.** Madagascar • Foulpointe, Mahavelona, *L.M.A. Du Petit-Thouars s.n.* P (P00135529!).

**Vernacular name**. Voanono (Betsimisaraka).

**GenBank information.** DNA sequences published by Kocyan et al. (2007): DQ535874, DQ536527, EF066328, EF066331.

**Distribution.** Endemic to Madagascar, where it is found in the East and eastern part of the Center (Fig. 11).

Eastern Domain: Antsinanana region, Foulpointe, *L.M.A. Du Petit-Thouars s.n.* (P).

Central Domain: Alaotra-Mangoro region, Analamazaotra, *G. Cremers 30671* (P), *H. Perrier de la Bâthie 6066, 6766, 8149* (P), *M. Keraudren-Aymonin 25372* (BR, P); Rainforest along the road from Moramanga to Anosibe, *J. Bosser 16615* (P); Mandraka forest, *C. d’ Alleizette 981* (P), *H. Perrier de la Bâthie 17297* (P); Ambatovy, *Rakotondrafara 748* (MO); Andranobe Forest, on the road to Andriamena, *J. Bosser 19851* (BR, P); Analamanga region: Anjozorobe, *Razakamalala 1940* (P); Vatovavy Region, Ranomafana NP, *H. Schaefer et al. 111, 123*, *127* (DBEV, TUM).

**Occurrence in Protected Areas.** Torotorofotsy New Protected Area, Analamazaotra NP, Mantadia NP, Ranomafana NP, Anjozorobe-Angavo Complex Protected Harmonious Landscape, and Ankeniheny-Zahamena Corridor Natural Resources Reserve.

**Ecology and habitat.** Restricted to the medium altitude moist evergreen forest, from 400–1,400 m asl, which is characterised by high rainfall (>1,200 mm per year) and no, or brief, dry season (< 4 months), and high air humidity (Gautier et al. 2018).

**Taxonomy.** Sister species to *A. bosseri* and *A. leandrii* (Schaefer et al. 2009).

**IUCN.** Following IUCN criterion B1 the species would be classified as Critically Endangered (CR), but we suggest using B2 which classifies the species as Least Concern (LC). The species is threatened by illegal logging and forest fires destroying the humid forest remnants.

***Ampelosycios* sp. nov. Ankarana**

Fig. 12

**GenBank information.** Has been sequenced by the authors (unpubl. data).

**Distribution.** Endemic to Madagascar, where it is only known from the North (Fig. 13).

Western Domain: Diana region, Ankarana Special Reserve, *Martine Bardot Vaucoulon 151* (P), *J.N. Labat 2792*, *2834* (P), *H. Schaefer & M.B. Andriamiharisoa 15*, *17, 18, 26*, *26B*, *37, 37B*, *37C* (TUM, DBEV); SAVA region, Daraina, *P. Ranirison & L.P.G. Nusbaumer 1151* (G).

**Occurrence in Protected Areas.** Ankarana Special Reserve, Loky Manambato Protected Harmonious Landscape.

**Figures 8–13.**
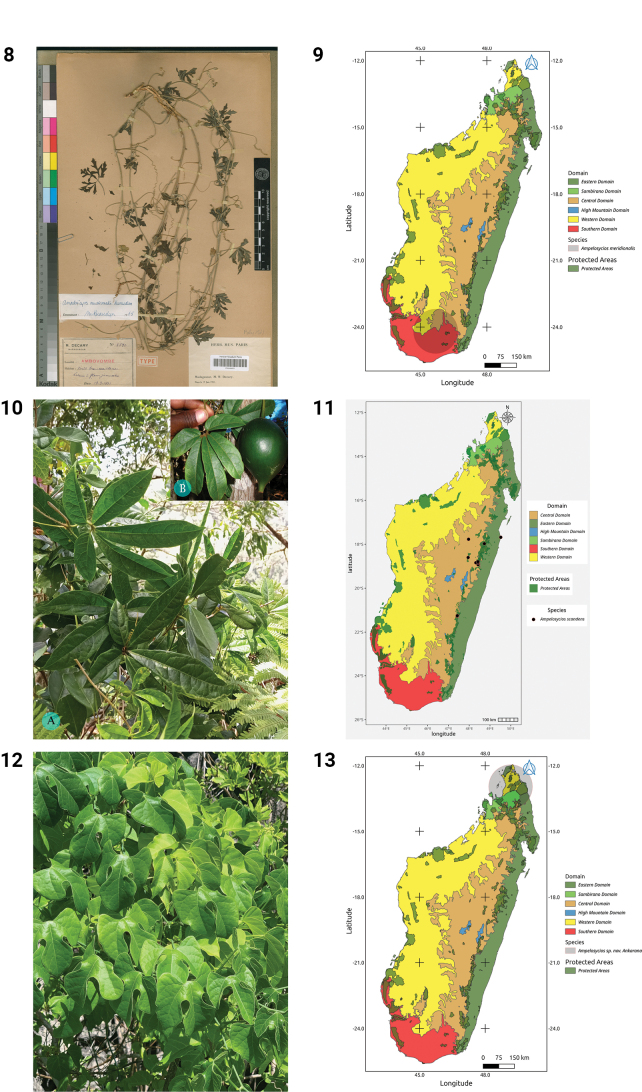
**8.***Ampelosycios meridionalis*, Holotype, between Tranoroa and Beloha, *J. Bosser 19154* (P). **9.** Distribution of *Ampelosycios meridionalis*. **10.***Ampelosycios scandens*. **A.** Habit; **B.** Fruit (photo A by MBA, Ranomafana NP, October 2022, B by A. Rakotondrafara, Moramanga, Ampitabe, Ambatovy, May 2008). **11.** Distribution of *Ampelosycios scandens*. **12.***Ampelosycios* sp. nov. Ankarana (photo by HS, Ankarana Special Reserve, December 2024). **13.** Distribution of *Ampelosycios* sp. nov. Ankarana.

**Ecology and habitat.** Dry deciduous forest on limestone formations (tsingy) and sand at 90–150 m asl., characterised by annual rainfall between 400–1,400 mm/year, with a pronounced dry season (4–9 months), high temperatures and low seasonal and daily temperature ranges (Gautier et al. 2018).

**Taxonomy.** Belongs to the genus *Ampelosycios* based on the presence of spoon-shaped stipels at the base of the petiole, but its leaf shape is different from all described species in this genus. Unpublished DNA data confirms its identity as a new species of *Ampelosycios* with closest relations to *A. leandrii* (Andriamiharisoa et al., in prep.).

**IUCN.** We suggest Critically Endangered (CR) based on IUCN criterion B1. The species is threatened due to the very small population size and illegal collection for ornamental purposes.

***Ampelosycios* sp. nov. Montagne des Français**

Fig. 14

**GenBank information.** DNA data available (unpubl.).

**Distribution.** Endemic to Madagascar where it has been found only in the extreme North (Fig. 15).

Western Domain: Diana region, Montagne des Français, Paysages Harmonieux d’Ambohitr’Antsingy, *H. Schaefer & M.B. Andriamiharisoa 4, 4A, 4C, 8* (TUM, DBEV).

**Occurrence in Protected Areas.** Ambohitr’Antsingy, Montagne des Français Protected Harmonious Landscape.

**Ecology and habitat.** Dry deciduous forest on sand at 200 m asl., characterised by annual rainfall between 400–1,400 mm, with a pronounced dry season of 4–9 months (Gautier et al. 2018).

**Taxonomy.** Belongs to the genus *Ampelosycios* based on the presence of spoon-shaped stipels at the base of the petiole and fimbriate petals, but its leaf shape is different from all described species in this genus and from the plants found at Ankarana. Unpublished DNA data shows that it is closely related to the clade including *A. leandrii, A.* spec. nov. Ankarana, and *A. bosseri* (Andriamiharisoa et al., in prep.).

**IUCN.** We suggest Endangered (EN) based on IUCN criterion B2. Threatened due to very small population size and high tourist pressure at Montagne des Français.

***Blastania* Kotschy & Peyr., Pl. Tinn.: 15 (1867).**

**Generic type.***Blastania cerasiformis* (Stocks) A. Meeuse.

**Worldwide Distribution.** Subtropical Africa to India, Sri Lanka, Pakistan and Madagascar (Schaefer 2020).

***Blastania lucorum* (Keraudren) H. Schaef., Syst. Bot. 42(1): 70. 2017**

Fig. 16

**Basionym.***Zombitsia lucorum* Keraudren, *Adansonia*, n.s., 3: 167 (1963).

**Synonym**: *Ctenolepis lucorum* (Keraudren) H. Schaef. & S.S. Renner, *Taxon* 60: 133 (2011).

**Holotype.** Madagascar • Toliara, Lisière de la forêt du Zombitsy (Sakaraha), Jan. 1962, *M. Keraudren 1337* P (P00475464!).

**Isotypes.** P (P00475460!, P00475461!, P00475462!, P00475463!).

**GenBank information**. DNA sequences published by Kocyan et al. (2007): DQ491024, DQ501260, DQ501273.

**Distribution.** Endemic to Madagascar, where it has been found in the South and West (Fig. 17).

Western Domain: Melaky region, Dokolahy, *J. Léandri 603* (P); Atsimo-Andrefana region, *M. Keraudren 1337* (MO, P), *J. Bosser 15765* (P, MO), and *H. Schaefer et al. 96*, *97* (DBEV, TUM).

Southern Domain: Atsimo-Andrefana region, Beza Mahafaly Special Reserve, *P.B. Phillipson 2541* (P, MO).

**Occurrence in Protected Areas.** Zombitse-Vohibasia NP and Bezà-Mahafaly Special Reserve.

**Ecology and habitat.** Moist semi-deciduous forests at 500 m asl., characterised by moderate rainfall (600–1,200 mm/year), pronounced dry season (6–8 months), high temperatures, and low annual and daily temperature ranges (Gautier et al. 2018); flowering Nov.–Feb. (Keraudren 1963a, 1966).

**Taxonomy.** The genus *Blastania* includes three species including, *B. cerasiformis* which is known from India and further west to Tropical Africa, *B. garcinii* from India and Sri Lanka, and *B. lucorum* endemic to Southwestern Madagascar (Renner and Pandey 2013). *Blastania lucorum* is sister to *B. cerasiformis* and splits from this one c. 7 ± 3 Ma (Schaefer et al. 2009; Lindner et al. 2017). Molecular studies, e.g., Schaefer et al. (2009), showed that *Z. lucorum* should be included in the genus *Ctenolepis*. Schaefer and Renner (2011) suggested the new combination *Ctenolepis lucorum* (Keraudren) H. Schaef. & S.S. Renner, but the genus name *Blastania* has priority (Lindner et al. 2017).

**IUCN.** We suggest Critically Endangered (CR) following IUCN criterion B1. The tiny population in the last moist semi-deciduous forest remnants is under massive pressure from grazing and forest fires.

***Cayaponia* Silva Manso, Enum. Subst. Braz.: 31 (1836).**

**Generic type.***Cayaponia diffusa* Silva Manso.

**Worldwide Distribution.** Central America, Mexico, Southern USA, West and Central Africa and Madagascar (Schaefer 2020).

**Cayaponia africana (Hook.f.) Exell var. madagascariensis Keraudren, Bull. Soc. Bot. France 107(3): 100. 1960.**

**Holotype.** Madagascar • Mahajanga, Ouest: bois humides, hauts Bemarivo, Boïna; Mar. 1907; *H. Perrier de la Bâthie 6776* P (P00135492!), our Fig. 18.

**Isotype.** P (P00135493!, P00475102!).

**GenBank information.** A single DNA sequence fragment available from Duchen and Renner (2010), HM015048.

**Distribution.** Endemic to Madagascar, where it has been found only in a small area in the West (Fig. 19).

Western Domain: Sofia region, upper Bemarivo, *H. Perrier de la Bâthie 6771, 6776*, *6779* (P); Betsiboka region, alluvial deposits of Betsiboka River, *H. Perrier de la Bâthie 1288* (P).

**Figures 14–19.**
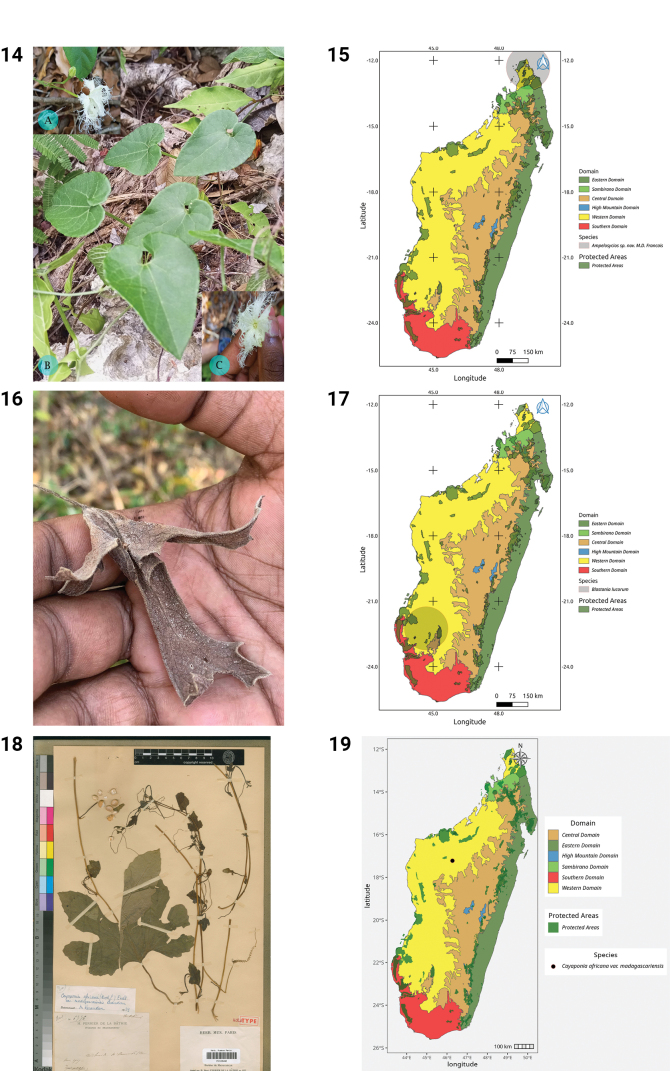
**14.***Ampelosycios* sp. nov. Montagne des Français. **A.** Male inflorescence; **B.** Habit; **C.** Male flower in full bloom (photos by MBA, Paysages Harmonieux d’Ambohitr’Antsingy, December 2023). **15.** Distribution of *Ampelosycios* sp. nov. Montagne des Français. **16.***Blastania lucorum*, dry leaf (photo by HS, Sakaraha-Vohibasia, October 2022). **17.** Distribution of *Blastania lucorum*. **18.**Cayaponia africana var. madagascariensis, Holotype, Boïna, *H. Perrier de la Bâthie 6776* (P). **19.** Distribution of Cayaponia africana var. madagascariensis.

**Occurrence in Protected Areas.** Not known from protected areas.

**Ecology and habitat.** Dry deciduous forest at c. 500 m asl., on sandy soil.

**Taxonomy.** The species *C. africana* is found in West Africa and in Madagascar, where it is represented by C. africana var. madagascariensis Keraudren (Keraudren 1968). The taxonomic status of this variety remains unclear due to the lack of material for phylogenetic or population-level studies (Duchen and Renner 2010).

**IUCN.** We suggest Data Deficient (DD), due to the limited number of occurrence data.

***Citrullus* Schrad. ex Eckl. & Zeyh., Enum. Pl. Afric. Austral.: 279 (1836), nom. cons.**

**Generic type.***Citrullus lanatus* (Thunb.) Matsum. & Nakai.

**Worldwide Distribution.** Eastern Mediterranean region, Africa, and Western Asia (Schaefer 2020).

***Citrullus mucosospermus* (Fursa) Fursa, Trudy Prikl. Bot. 81: 111 (1983)**

Fig. 20

**Holotype.** Ghana • 5 Aug. 1957, *N.P. Oltarshevskyi 3833* (WIR k-3742).

**Basionym.**Citrullus lanatus subsp. mucosospermus Fursa, *Bot. Zhurn*. (Moscow & Leningrad) 57: 38 (1972).

**Vernacular name**. Voazavo (Tanala).

**GenBank information.** DNA sequences of this taxon published by Chomicki and Renner (2015), and Shi et al. (2017)KY430686, NC033899, KY430687, KY430693, KX773720, KX773718, KP036543, KP058579, KM281404, and KP036531; sequences of Malagasy material (unpubl. data) will be made available.

**Distribution.** Native to Ghana, Guinea, Nigeria, Senegal, and Sudan (POWO 2023). In Madagascar, it is mainly found in the occidental part (Fig. 21), its indigenous status remains doubtful.

Occurs in the Western, Central, and Southern Domains: Zombitsy-Vohibasia NP, *H. Schaefer et al. 84, 85, 86*, *87* (DBEV, TUM); Ambiamena, *H. Schaefer et al. 92* (DBEV, TUM); Ambinany, *H. Schaefer et al. 98, 99* (DBEV, TUM).

**Occurrence in Protected Areas.** Ranobe PK32 New Protected Area, Ankarafantsika NP, and Zombitse-Vohibasia Protected Area.

Note. All specimens identified by Keraudren (1966) as *Citrullus colocynthis* (L.) Schrad. and revised by the authors in P belong, in fact, to *C. mucosospermus*.

**Ecology and habitat.** Annual plant of the seasonally dry tropical biome, where it grows in agricultural areas and on riverbanks. According to herbarium label information (*J.M. Hildebrandt 3435* (P)), *C. mucosospermus* is spread by wild boar, dogs and humans, who eat the fruit and swallow the seeds.

**Uses.** In West African countries, *Citrullus mucosospermus*, also known as ‘egusi’ watermelon, is cultivated mainly for its nutritious, oleaginous seeds, which can be used either to extract edible oil or to thicken soups (Achigan-Dako et al. 2008). This kind of usage is not known from Madagascar, where it is rather used as a source of water for local communities in the arid zones and to feed livestock. The flesh is low in sugar and tastes more like cucumber than melon.

**Taxonomy.** According to Guo et al. (2013) and Renner et al. (2017), *C. mucosospermus* is sister to *C. lanatus* and might better be treated as a subspecies of the latter.

**IUCN.** We suggest Least Concern (LC), based on IUCN criterion B2.

***Corallocarpus* Welw. ex Hook.f., G.Bentham & J.D.Hooker, Gen. Pl. 1: 831 (1867).**

**Generic type.***Corallocarpus welwitschii* (Naudin) Hook.f.

**Worldwide Distribution.** Africa, Arabia, India, Pakistan, and Madagascar (Schaefer 2020).

***Corallocarpus grevei* (Keraudren) Keraudren, Fl. Madagasc. 185: 72 (1966)**

Fig. 22

**Basionym.***Calyptrosicyos grevei* Keraudren, *Compt. Rend. Hebd. Séances Acad. Sci.* 248: 3593 (1959).

**Holotype.** Madagascar • Mahajunga, sable-Majunga, Ouest, bois, Jan. 1919, *H. Perrier de la Bâthie 12348* P (P00135484!).

**Isotypes.** P (P00135485!, P00135486!).

**GenBank information.** Has been sequenced by the authors (unpubl. data).

**Distribution.** Endemic to Madagascar where it has been found mainly in the South and in a few spots in the West and Center (Fig. 23).

Western Domain: Boeny region, Mahajanga, *H. Perrier de la Bâthie 12348* (P); Amboanio, *H. Perrier de la Bâthie 1401* (P); Ankarafantsika NP, *H. Schaefer et al. 41* (TUM, DBEV).

High Mountain Domain: Haute Matsiatra, Andringitra, *Jean De Dieu Ratoto 1379 RN* (P).

Southern Domain: Atsimo-Andrefana region, Toliara, *J. Bosser 13572* (P), *T. B. Croat 30927* (P), *M. Keraudren 738* (P); Andatabo, *R. Randrianaivo 334* (WAG); N Fiherenana River, *J. Léandri 3798* (P); on the road from Andranovory to Vatolatsaka, *L. Allorge 2264* (P); Anjamala, *M. Keraudren 677* (P); Mahafaly limestone plateau, near Ankilirano, *M. Keraudren 824* (P, BR), *849, 850, 866* (P); Zombitsy NP, *P. Morat 3798* (P), *H. Schaefer et al. 80*, *81* (DBEV, TUM); Antsepoka, *Ramon 468* (P, TAN); Betioky, *M. Keraudren 771* (P); along the road from Ampanihy to Ampotaka (Menarandra Downstream), *M. Keraudren 903* (P); Befamata, *H. Poisson 422* (P); Ambatry, *J. Bosser 13564* (P); Ampandrandava, *A. Seyrig 711*, 711 B (P), *Jardin Botanique Tananarive team 6453* (P); Ampanihy, *A.M. Homolle 1662* (P); Antanimora, *H. Humbert 28799bis* (P); Belalanda, Ranobe PK32, *Ranaivojaona 1667* (US), *M. Andrianjafy 1667* (P); Beomby, *J. Bosser 13557* (P), *M. Keraudren 794*, *797*, *817* (P); from Ejeda to Fofadrevo, *M. Keraudren 1437* (P); Fiherenana pass about 40 km from Miary, *M. Keraudren 768* (P); Androy region, Ambovombe, *R. Decary 8600* (P); Ambatomika, *M. Keraudren 1081* (P); Behara, *Luckow, M 4123* (WAG); Tranomaro New Protected Area, *H. Schaefer et al. 5, 6A, 6B* (DBEV, TUM); Tsihombe, *M. Peltier 2851* (P). Anosy region, Anarafaly, *J. Bosser 3760* (P), *M. Keraudren 1000* (P); on the road from Amboasary to Fort-Dauphin, kilometer point n°434, *M. Keraudren 1027* (P); Andohahela NP, *Randriambololona* 63 (MO), *H. Schaefer et al. 60, 64* (DBEV, TUM); Menabe region, Morondava, *Grevé 125* (P).

**Occurrence in Protected Areas.** Andringitra NP, Ranobe PK32 New Protected Area, Andohahela NP, Ankodida Harmonious Protected Landscape, Tsinjoriake Harmonious Protected Landscape, Ankarafantsika NP, and Zombitse-Vohibasia NP.

**Ecology and habitat**: Riverine and coastal forest on brown, red, or white sands, in dry spiny thicket from 10–700 m asl., characterised by low and irregular rainfall (< 600 mm/year), dry season of over 10 months, high to very high temperatures, significant annual temperature ranges, and high daily temperature ranges (Gautier et al. 2018); flowering throughout the year (Keraudren 1966).

**Taxonomy.** Unresolved.

**IUCN.** We suggest Least Concern (LC) based on IUCN criterion B2.

***Corallocarpus perrieri* (Keraudren) Keraudren, Fl. Madagasc. 185: 68 (1966)**

Fig. 24.

**Basionym.***Calyptrosicyos perrieri* Keraudren, *Compt. Rend. Hebd. Séances Acad. Sci.* 248: 3593 (1959).

**Holotype.** Madagascar • Toliara, Tsimanampetsotsa, Manampetsa, sables, Apr. 1933, *H. Perrier de la Bâthie 19118* P (P00462267!).

**Isotypes.** P (P00462268!, P00462269!).

**GenBank information.** Has been sequenced by the authors (unpubl. data).

**Distribution.** Endemic to Madagascar, where it occurs only in the South (Fig. 25).

Southern Domain: Atsimo-Andrefana region, Toliara, *M. Keraudren 722, 724* (P), *J. Bosser 10151* (P), *TB Croat 30828* (WAG, P), *L. Allorge 2794* (P), *Ramon 388* (MO, TAN), *M. Keraudren 601* (BR); Anakao, *Ramon 364* (MO, TAN); from Toliara to Manombo, *P. Morat 3514* (P); Manombo, *R. Decary 18696* (P); North of Toliara, *J. Bosser 13563* (P); Ankilibe, *M. Keraudren 601* (P); Ranobe PK32, *Andrianjafy 1675* (BR, P, US, MO), *M. Keraudren 682*; Toliara II, Tsivonoakely, *Ranaivojaona 187* (US); Belemboka, *J. Dequaire 27399* (P); Efoetsy, *M. Keraudren 883* (P); Itampolo, *M. Keraudren 890* (P); Morombe, *R. Decary 18744, 18745* (BR, P); on the roadside of the National Road n°10, *R.N. Ravelonanahary 3651* (P); from Toliara to Manombo, *P. Morat 3514* (P); Sarodrano, *P. Morat 2502* (P); Tokilisy, *M. Peltier 5490* (P); from Tulear to Morombe, *M. Keraudren 707* (P); Tsimanampetsotsa, *H. Perrier de la Bâthie 6790, 19118* (P), *M. Peltier 3148, 3153, 3154* (P), *H. Humbert 5373* (P); Ifotaka, Anarafaly, *Z.S. Rogers 484* (P).

**Occurrence in Protected Areas.** Ranobe PK32 New Protected Area, Tsimanampetsotsa NP, Tsinjoriake Harmonious Protected Landscape, Harmonious Protected Landscape of the Mangoky–Ihotry Wetland Complex.

**Ecology and habitat.** Dry spiny thicket, with *Adansonia rubrostipa* and *Delonix floribunda* from 5–170 m asl.; flowering throughout the year (Keraudren 1966).

**Taxonomy.** Unresolved.

**IUCN.** We suggest Least Concern (LC), based on IUCN criterion B2.

**Figures 20–25.**
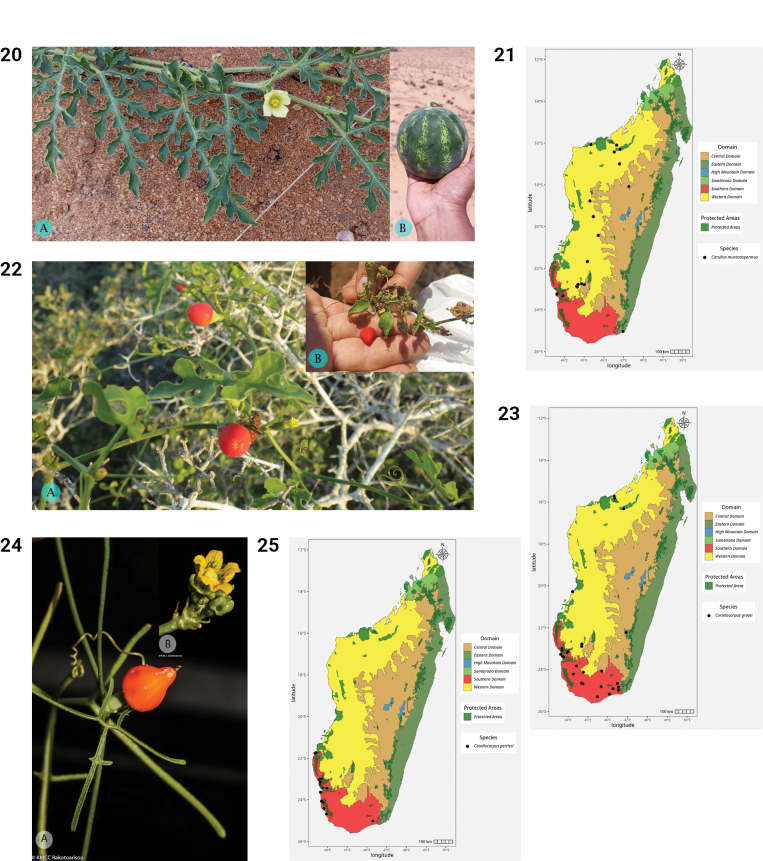
**20.***Citrullus mucosospermus*. **A.** Shoot with leaves and male flower; **B.** Fruit (photos by MBA, Zombitsy-Vohibasia, October 2022). **21.** Distribution of *Citrullus mucosospermus*. **22.***Corallocarpus grevei*. **A.** Habit and mature fruit; **B.** Young fruit (photos by L. Ramon, Toliara, Antsepoka, February 2017 (**A**) and by MBA, Andohahela NP, October 2022 (**B**)). **23.** Distribution of *Corallocarpus grevei*. **24.***Corallocarpus perrieri*. **A.** Shoot with mature fruit; **B.** Male flower (photos **A, B** by S.E. Rakotoarisoa, Toliara, Ampanihy, Ankazoabo, January 2019). **25.** Distribution of *Corallocarpus perrieri*.

***Corallocarpus poissonii* Cogn. A.L.P.P.de Candolle & A.C.P.de Candolle, Monogr. Phan. 3: 651 (1881)**

Fig. 26

**Syntypes.** Comores • Mayotte, Bords de la mer à Pammanzi, côte ouest parmi les broussailles; Feb. 1850; *L.H. Boivin. s.n.* P (P00135487!, P00135488!, P00135489!, P00135490!). Madagascar • Port Leven, *M. Vesco* P (P00475342!); sine loc., *Baron 6457* (K), n.s.

**GenBank information.** Malagasy material has been sequenced by the authors (unpubl. data).

**Distribution.** Endemic to Madagascar (where it is only known from the extreme North, Fig. 27) and Comoros, where the type specimens originated. The full distribution of *C. poissonii* is unclear due to confusion with *C. bainesii* (Hook.f.) A. Meeuse, which is a genetically distinct continental African species originally described from Botswana (Andriamiharisoa et al., in prep.).

Western Domain: Diana Region, Andranovondronina, Antsisikala, Ambatoara, Analamangidy, *R. Guittou 99* (P); Vovo Village, *T.G, Razafindrabaeza, et al. 1702* (K); Port Leven, *M. Vesco 1850* (P); Montagne des Français, *H. Schaefer & M.B. Andriamiharisoa 1A, 1B, 2*, 5, 6 (TUM, DBEV).

**Occurrence inside Protected Areas.** Protected Harmonious Landscape Ambohitr’Antsingy – Montagne des Francais and Protected Harmonious Landscape of Andrafiamena Andavakoera.

**Ecology and habitat.** Dry deciduous forest on limestone and littoral thicket on brown and red sandy soils, from sea level to 450 m asl.

**Taxonomy.** Unresolved.

**IUCN.** We suggest Endangered (EN) based on IUCN criterion B2.

***Cucumis* L., Sp. Pl. 1011. 1753.**

**Generic type.***Cucumis sativus* L.

**Worldwide Distribution.** Native to Africa, Asia, Australia, and Madagascar (Schaefer 2020).

***Cucumis sacleuxii* Paill. & Bois, Bull. Soc. Natl. Acclim. France 37: 371 (1890)**

Fig. 28

**Neotype.** Tanzania • Zanzibar, Massazine, cliff area, grassland or bush, 15^th^ Jul. 1961, *H. Faulkner 2865* BR (BR0000008887313!), designated by Kirkbride, Biosyst. Monogr. Cucumis 41 (1993).

**Synonyms.**Cucumis sativus var. usambarensis Zimm. *Cucurbitac*. 2: 179 (1922); *Oreosyce aspera* Cogn., *Pflanzenr.*, IV, 275 I: 268 (1916).

**GenBank information.** DNA sequences of the species (but not from Malagasy material!) published by Renner et al. (2007), Ghebretinsae et al. (2007), and Sebastian et al. (2010): EF595895, EF595945, DQ785838, DQ785852, DQ785880, EF093520, DQ785866, HM597099; Malagasy material has been sequenced by the authors (unpubl. data).

**Distribution.** Native to East Africa (POWO 2023) and possibly Madagascar, where it has often been confused with *C. sativus*, so probably more widespread than indicated in the map (Fig. 29).

Western Domain: Diana region, Joffreville, *H. Schaefer & M.B. Andriamiharisoa 40* (January 2024); Sofia region, Befandriana Nord, *Jardin Botanique d’Antananarivo team 5098* (P); Boeny region, Bevazaha, *N. Ramamonjisoa 1627* (P); Ankarafantsika forest, near Ampijoroa, *M. Keraudren 1255* (P); Ihorombe region, Isalo, *M. Keraudren 462* (P).

Central Domain: Sofia region, Betainkankana, Ankaizina, on the forest border, *J. Bosser 2633* (P).

Eastern Domain: Ilaka-Est, *J. Bosser 17024* (P); Fort-Dauphin, *R. Decary 9944* (P); Andohahela NP, *H. Schaefer et al. 46* (DBEV, TUM); outside Andohahela NP, *H. Schaefer et al. 58*, *72* (DBEV, TUM).

**Occurrence in Protected Areas**. Andohahela NP, Ankarafantsika NP, Montagne d’Ambre NP, and Montagne des Français Protected Harmonious Landscape.

**Ecology and habitat.** In seasonal zones with moderate annual rainfall, in clearings around human settlements, in wetlands, wasteland, and the forest understory, mainly on clay soils, from sea level to 700 m asl.

**Uses.** The fruits are edible but no information on uses in Madagascar is available.

**Taxonomy.***Cucumis sacleuxii* is sister to *C. rostratus* and *C. metuliferus* (Sebastian et al. 2010).

**IUCN.** We suggest Least Concern (LC) based on IUCN criterion B2.

***Cucumis subsericeus* Hook. f, Fl. Trop. Afr. 2: 545 (1871)**

Fig. 30

**Holotype.** Angola • Cuanza Norte, Pungo Andongo, *F.M.J. Welwitsch 838* (BM).

**Synonyms.***Oreosyce africana* Hook. F., Fl. Trop. Afr. 2: 545 (1871); *Cucumis oreosyce* H. Schaef., Blumea 51: 171 (2007) nom. superfl.; *Cucumis cecilii* N.E.Br., *Bull. Misc. Inform. Kew* 1906: 104 (1906); *Cucumis parvifolius* Cogn. ex Scott Elliot, *J. Linn. Soc.*, Bot. 29: 19 (1891); *Hymenosicyos subsericeus* (Hook.f.) Harms, *Notizbl. Bot. Gart.* Berlin-Dahlem 8: 487 (1923); *Oreosyce subsericea* (Hook.f.) A. Meeuse, *Bothalia* 8: 22 (1962).

**Vernacular name.** Voatangondolo (Merina, Betsileo).

**GenBank information.** DNA sequences of this species (but not from Malagasy material!) published by Ghebretinsae et al. (2007): EF595956, EF595957; by Kocyan et al. (2007): DQ535833, DQ536576; and by Sebastian et al. (2010): HM597090.

**Distribution**: Native throughout Tropical Africa from Bioko to Ethiopia, South Africa, and Madagascar (POWO 2023), where it is widespread mainly in the Centre and higher mountains (Fig. 31).

High Mountain Domain: Sava, Andapa, Ambohimirahavavy, *C. Rakotovao 2520* (MO, P); Beampoko Forest, *C. Randrianarivelo 380* (P); East peak of Ambohimirahavavy, *C. Rakotovao 2402* (P); forest edge, *H. Humbert 25663* (P); Tsaratanana massif, camp no. 2, peak, Mahavavy Valley, *P. Morat 2345, 2290* (P); Tsaratanana, *H. Perrier de la Bâthie 16175* (P); southern flank of Antsianongatalata, *H. Humbert 18396* (P); Ankaizinana, *R. Decary 1796, 1829*, *1993* (P); Region of Melaky, Ambohimirahavavy, *H. Andriamiarinoro 14* (P); upper Bemafo, *H. Humbert 25157* (P); rocks on the banks of Bemafo, *Buerki 91* (MO, P, TAN).

Sambirano Domain: Diana region, Ambilobe, Marivorahona, massif of Tsaratanana, *H. Humbert et al. 25792* (P).

Central Domain: Analamanga region, Antananarivo, *C. d’ Alleizette s.n.* (P); Ilafy, *R. Decary s.n.* (P), *Peltier 3822* (P); Ambohimanga, *Waterlot 56* (P); Vakinankaratra region, Ankaratra, *Scott Elliot G.F. 1947* (BR); Ankaratra, *Académie Malgache s.n.* (P); around Tritriva Lake, *H. Perrier de la Bâthie 3763*, *13069* (P); Ihorombe region, Fianarantsoa, Ivohibe, *R. Decary 5642* (P).

Eastern Domain: Analamanga region, Mandraka, *M. Keraudren-Aymonin 25401* (P); kilometer point n°23 of National Road from Antananarivo to Toamasina, *J. Bosser 13401* (P); Alaotra-Mangoro Region, Imerimandroso, *R. Decary 3957* (P); Zahamena, Andrangivalo massif southwest of Alaotra Lake, Natural Reserve no. 3 of Zahamena, Onibe watershed, *H. Humbert 17756* (P); wet undergrowth, Antsihanaka forest, *Jardin Botanique Tananarive team 2258* (P); Ihorombe region, Sakamalio, Sakamalio valley, *H. Humbert 13397* (P); Anosy region, Taolagnaro, Trafonaomby, Mountain, *B. Randriamampionona 712* (P); Andohahela massif, *H. Humbert 13620 bis* (P); Betroka, Kalambatritra Protected Area, *R. Razakamalala 2002* (P); Androy region, upper Mandrare Watershed, *H. Humbert 6522* (P).

**Figures 26–31.**
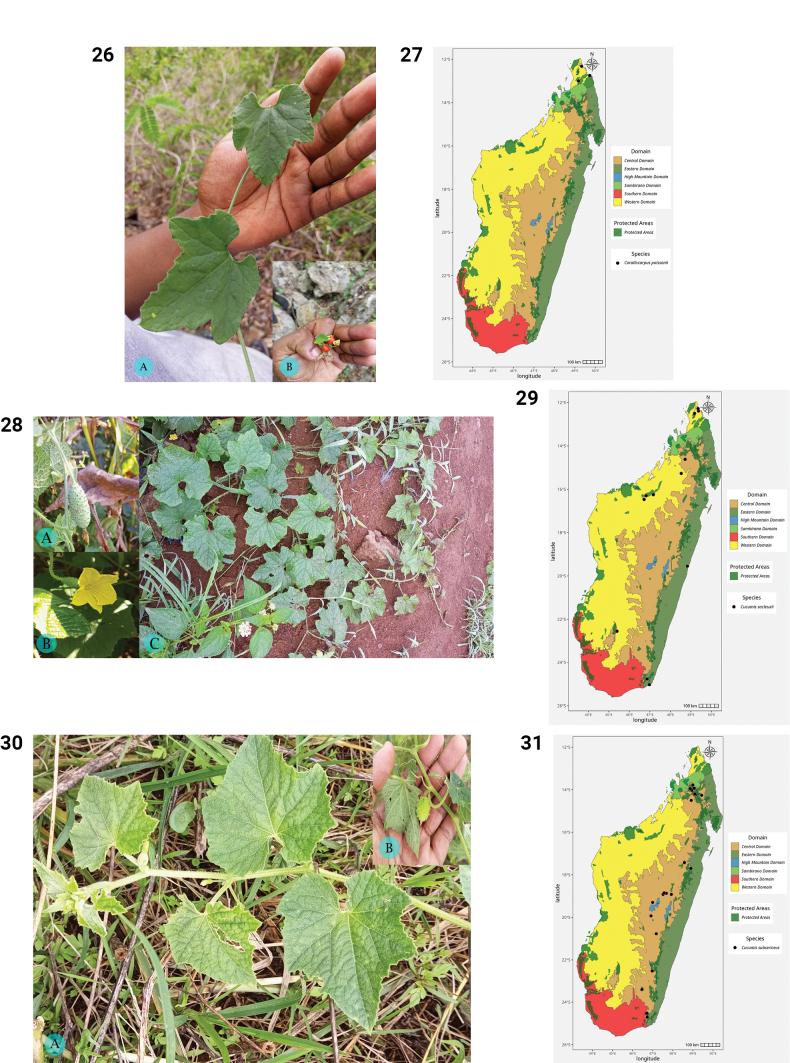
**26.***Corallocarpus poissonii*. **A.** Habit; **B.** Fruits (photos by MBA, Montagne des Français, December 2023). **27.** Distribution of *Corallocarpus poissonii*. **28.***Cucumis sacleuxii*. **A.** Young fruit; **B.** Female flower; **C.** Habit (photos by MBA, Andohahela NP (**A, B**), October 2022 and Montagne d’Ambre NP (**C**), December 2023). **29.** Distribution of *Cucumis sacleuxii*. **30.***Cucumis subsericeus*. **A.** Habit; **B.** Young fruit (photos by MBA, Ambatofitorahana, March 2023). **31.** Distribution of *Cucumis subsericeus*.

**Occurrence inside Protected Areas**: New Protected Area of Ranobe PK32, Andohahela NP, Protected Harmonious Landscape of Lac Alaotra, Protected Harmonious Landscape of the Mangoky Ihotry Wetland Complex, Natural Resource Reserve of the Marojejy-Anjanaharibe South-Tsaratanàna Corridor (Northern Part), Tsaratanàna Strict Nature Reserve, and Kalambatritra Special Reserve.

**Ecology and habitat.** Forest edges and clearings, burnt montane ericoïd thicket, wetlands, and grasslands, on gneissic laterite and rocky outcrop areas, climbing on trees or trailing along the ground, 1,000–2,600 m asl. The species’ habitat is characterised by high rainfall (>1,200 mm/year), no or only a brief dry season (<4 months), high air humidity (mist), an average temperature with low seasonal variation, and increasing daily temperature range and solar radiation with altitude (Gautier et al. 2018); flowering throughout the year (Keraudren 1966).

**Uses.** On the African mainland, *C. subsericeus* filtrate is injected to treat gonorrhea (Yineger and Yewhalaw 2007). No such use has been documented in Madagascar.

**Taxonomy.***Cucumis subsericeus* is sister species to *C. bryoniifolius* (Merxm.) Ghebret. & Thulin (Renner et al. 2007).

**IUCN.** We suggest Least Concern (LC) based on IUCN criterion B2.

***Cucumis* sp. nov. (aff. cinereus)**

Fig. 32

**GenBank information.** Has been sequenced by the authors (unpubl. data).

**Distribution.** Endemic to the South-western of Madagascar, where it is known only from a few collections from the Zombitsy area (Fig. 33).

Western Domain: Atsimo-Andrefana region, Sakaraha, Zombitsy NP, *M. Keraudren 480* (P), *J. Bosser 19394* (P); Plateau Mahafaly, western Betioky, *H. Humbert & R. Capuron 29458* (P).

**Occurrence in Protected Areas.** Zombitsy-Vohibasia NP.

**Ecology and habitat.** Moist semi-deciduous forest, on limestone soil (Keraudren 1966).

**Taxonomy.** Unresolved.

**IUCN.** We suggest Critically Endangered (CR) following IUCN criterion B1 and B2. Since the last collection dates to the 1960s, the species might have become extinct, but a detailed exploration of its former range is needed.

***Cucumis* sp. nov. (aff. hirsutus)**

Fig. 34

**GenBank information.** Has been sequenced by the authors (unpubl. data).

**Distribution**: Endemic to Madagascar, where only a few collections from the Centre and the North are known (Fig. 35).

High Mountain Domain: Haute Matsiatra region, Andringitra, *H. Perrier de la Bâthie 14403* (P); Between Ivato and la Mania, *H. Perrier de la Bâthie 12367* (P).

Central Domain: Diana region, Montagne d’Ambre, Les Rousettes, *M. Keraudren 1652* (P).

**Occurrence in Protected Areas.** Montagne d’Ambre and Andringitra NP.

**Ecology and habitat.** Montane ericoïd thicket, 1200–1800 m asl., characterised by important rainfall, no very pronounced dry season (potential for frost), and low annual temperature ranges, high daily temperature ranges and strong solar radiation (Gautier et al. 2018); flowering Feb.–March.

**Taxonomy.** Unresolved.

**IUCN.** We suggest Critically Endangered (CR) based on IUCN criterion B1 and B2. Since the last collection dates to the 1960s, the species might have become extinct, but a detailed exploration of its former range is needed.

***Cyclantheropsis* Harms, Bot. Jahrb. Syst. 23: 167. 1896.**

**Generic type.***Cyclantheropsis parviflora* (Cogn.) Harms.

**Worldwide Distribution.** East and South tropical Africa and Madagascar (Schaefer 2020).

***Cyclantheropsis madagascariensis* Keraudren, Fl. Madagasc. 185: 164 (1966)**

Fig. 36

**Holotype.** Madagascar • Anadabolava (moyen Mandrare), vestige de forêt sèche sur rocailles, 1 Feb. 1962, *J. Bosser 15723* P (P00135356!).

**Isotype.** P (P00135357!)

**GenBank information.** No DNA sequences available for this taxon.

**Distribution.** Endemic to Madagascar, where it was found in the West, North and South (Fig. 37).

Western Domain: Diana region, Antsiranana, *G. Rahajasoa et al.* (MO, P, WAG); Melaky, Tsingy de Bemaraha, *C.C.H. Jongkind 3512* (BR, WAG); Atsimo-Andrefana region, Anadabolava, *J. Bosser 15723* (P), *M. Keraudren 1089* (P); Atsimo-Andrefana region, Analavelona forest, *H. Humbert 19726* (P).

**Occurrence in Protected Areas.** Natural Monument of the Sacred Forest of Alandraza Analavelo, Tsingy de Bemaraha NP, and Ankarana Special Reserve.

**Ecology and habitat.** Dry deciduous forests on limestone formation (tsingy), c. 180 m asl.

**Taxonomy.** Unresolved.

**IUCN.** Based on IUCN criterion B1, this species would be classified as Least Concern (LC). However, since we failed to confirm the presence of the species during our expeditions, we suggest classification based on criterion B2 as Critically Endangered (CR).

***Gerrardanthus* Harv. ex Benth. & Hook.f., Gen. Pl. 1: 840 (1867)**

**Generic type.***Gerrardanthus macrorhizus* Harv. ex Benth. & Hook.f.

**Worldwide Distribution.** Tropical Africa and South Africa (Schaefer 2020), and Madagascar.

**Figures 32–37.**
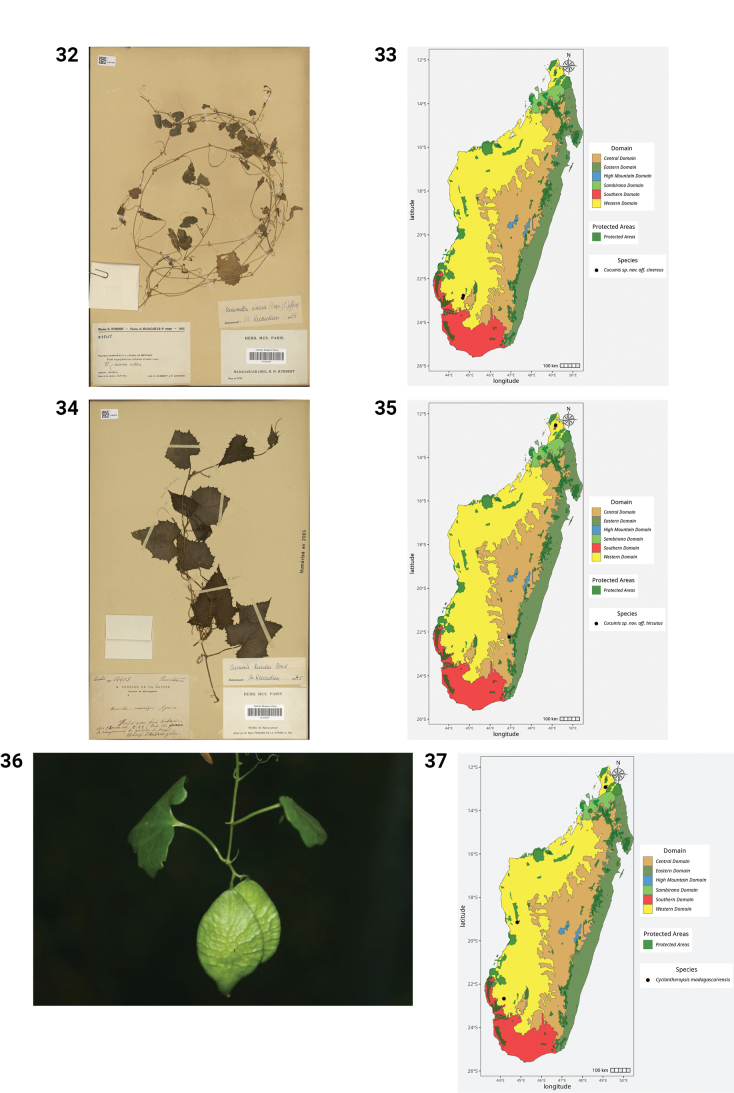
**32.***Cucumis* sp. nov. (aff. cinereus), Sakaraha, Zombitsy, *H. Humbert & R. Capuron 29458* (P). **33.** Distribution of *Cucumis* sp. nov. (aff. cinereus). **34.***Cucumis* sp. nov. (aff. hirsutus), Massif d’Andringitra, *H. Perrier de la Bâthie 14403* (P). **35.** Distribution of *Cucumis* sp. nov. (aff. hirsutus). **36.***Cyclantheropsis madagascariensis*, elliptic fruit (photo by F. Rakotoarivony, Sakaraha, Mahaboboka-Milenaky, December 2017). **37.** Distribution of *Cyclantheropsis madagascariensis*.

***Gerrardanthus grandiflorus* Gilg ex. Cogn. in H.G.A. Engler, Pflanzenr. IV, 275 I: 23 (1916)**

Fig. 38

**Syntypes.** Tanzania • Usambara: Urwald bei Derema, um 800–900 m ü.M., *Scheffler 31* (B, n.s.); Amani, 1000 m ü.M., *Braun 795*, *1129* (B, n.s.), *Grote 5031* (B, n.s.).

**GenBank information.** DNA sequences of this taxon (but not from Malagasy material!) published by Kocyan et al. (2007) and Schaefer and Renner (2010): GQ163343, DQ535805, DQ536668, GQ163220, DQ536768, DQ536624, GQ163097, GQ162967.

**Distribution.***Gerrardanthus grandiflorus* is known from Tanzania and Kenya (POWO 2023).

In Madagascar, the species has been collected only in one location in the West (Fig. 39).

Western Domain: Melaky region, Southern Beanka, Sarodrano, Andoloposa, *R.F. Bolliger et al. 68, 321 R.M. Hanitrarivo et al. 256* (G).

**Occurrence in Protected Areas.** Beanka Protected Harmonious Landscape.

**Ecology and habitat.** Dry deciduous forest with eroded limestone blocks, undergrowth with *Pandanus* sp., c. 200–400 m asl., characterised by annual rainfall between 400–1,400 mm/year, with a pronounced dry season (4–9 months), high temperatures and high daily temperature ranges (Gautier et al. 2018).

**Taxonomy.** Unresolved.

**IUCN.** We suggest Critically Endangered (CR) based on IUCN criterion B1 and B2.

***Kedrostis* Medik., Philos. Bot. 2: 69. 1791.**

**Generic type.***Kedrostis africana* (L.) Cogn.

**Worldwide Distribution.** Tropical and Subtropical Africa, Arabia, Asia (India, Sri Lanka, Western Malesia), and Madagascar (Schaefer 2020).

***Kedrostis cogniauxii* Keraudren, Bull. Soc. Bot. France 108: 241 (1961)**

Fig. 40

**Holotype.** Madagascar • Antsiranana, Forêt de Loucoubé, Nossi-bé, Mar. 1853, *L.H. Boivin s.n.* P (P00135476!).

**GenBank information.** No DNA sequences available.

**Distribution.** Endemic to Madagascar, where it has been collected in one place in the North (Fig. 41).

Sambirano Domain: Diana region, Forêt de Loucoubé, Nossi-bé, Mar. 1853, *L.H. Boivin s. n.* (P).

**Occurrence in Protected Areas.** Lokobe NP.

**Ecology and habitat**: Lowland evergreen moist forest, at c. 200 m asl., characterised by high rainfall (>1,200 mm/year), and no or brief dry season, (<4 months), high temperature, narrow seasonal and daily ranges (Gautier et al. 2018).

**Taxonomy.** Unresolved.

**IUCN.** We suggest Data Deficient (DD) due to the limited occurrence data of the species.

***Kedrostis dissecta* Keraudren, Bull. Soc. Bot. France 108: 241 (1961).**

**Holotype.** Madagascar • Ankarafantsika (7e Reserve), Sainte Marie, plateau, 150 m, *Service Forestier Madagascar 60* P (P00135477!), our Fig. 42.

**GenBank information.** No DNA sequences available.

**Distribution.** Endemic to Madagascar, where it is found in the North, East and West (Fig. 43).

Western Domain: SAVA region, Daraina, Solaniampilana-Moroadabo forest, *L. Gautier 4520* (P); Boeny region, Ankarafantsika, Reserve no. 7, Sainte Marie plateau, *Service Forestier Madagascar 60* (P).

Central Domain: Alaotra-Mangoro, Antokazo, Manakambahiny, *G. Cours 304* (P).

**Occurrence inside Protected Areas.** Ankarafantsika NP, Loky-Manambato Protected Harmonious Landscape.

**Ecology and habitat.** Dry deciduous forest, 100–1,100 m asl.

**Taxonomy.** Unresolved.

**IUCN.** Based on IUCN criterion B1 the species is classified as Critically Endangered (CR).

***Kedrostis elongata* Keraudren, Bull. Soc. Bot. France 108: 242 (1961)**

Fig. 44

**Holotype.** Madagascar • Forêt de la Mandraka, 1,200 m, 1 Jan. 1927, *H. Perrier de la Bâthie 16898* P (P00135478!).

**Isotypes.** P (P00135479!, P00135480!, P00792646!).

**Vernacular name.** Voatangondolo (Sakalava).

**GenBank information.** Has been sequenced by the authors (unpubl. data).

**Distribution.** Endemic to Madagascar, where it is widespread in the West, North, and Centre (Fig. 45).

Sambirano Domain: Nosy Be, *J.M. Hildebrandt 2960* (W).

Western Domain: Diana region, Andavakafanihy Mountain, Ankarana Special Reserve, *G. Cours 559*7 (P), *M. Keraudren 1731* (P), *H. Schaefer & M.B. Andriamiharisoa 21* (TUM, DBEV), *M. Bardot-Vaucoulo*n 1362 (P), *M. Bardot-Vaucoulon 1425, 1425 bis* (P, TAN, MO, K, G); Boeny region, Mahajanga, seashore, *H. Humbert 7166* (P); Ankarafantsika, *H. Perrier de la Bâthie 6775* (P), near the forest station of Ampijoroa, *M. Keraudren 1203* (P), Ankarafantsika NP, *H. Schaefer et al. 22, 23, 25, 26, 27, 29, 30, 31, 33, 35, 39, 40* (TUM, DBEV).

Central Domain: Analamanga region, Ankazobe, Manankazo, *M.J. Bosser 14481*, *R. Decary 17202* (P); in Mandraka forest, *H. Perrier de la Bâthie 16898* (P); Betsiboka region, in Ankaladiny; on Betsiboka riverbank, *H. Perrier de la Bâthie 1509* (P); in Firingalava, *H. Perrier de la Bâthie 588* (P).

Eastern Domain: East-Manakana township, *C. Rakotovao 11265* (P); East-Manakambahiny, near East-Manakampanihy village, *M. Keraudren 1164* (P).

Southern Domain: Atsimo-Andrefana, from Tuléar to Sakaraha, *M. Keraudren 744* (P); Menabe region, East of Mahabo, near Manamby on RN35, *Phillipson 6137* (MO).

**Occurrence inside Protected Areas.** Ankarana Special Reserve, Ankarafantsika NP, and Zahamena NP.

**Ecology and habitat.** Dry deciduous, and medium altitude moist evergreen forests, growing on riverbanks in shaded undergrowth or in clearings, at 100–1,200 m asl.; flowering throughout the year (Keraudren 1966).

**Figures 38–43.**
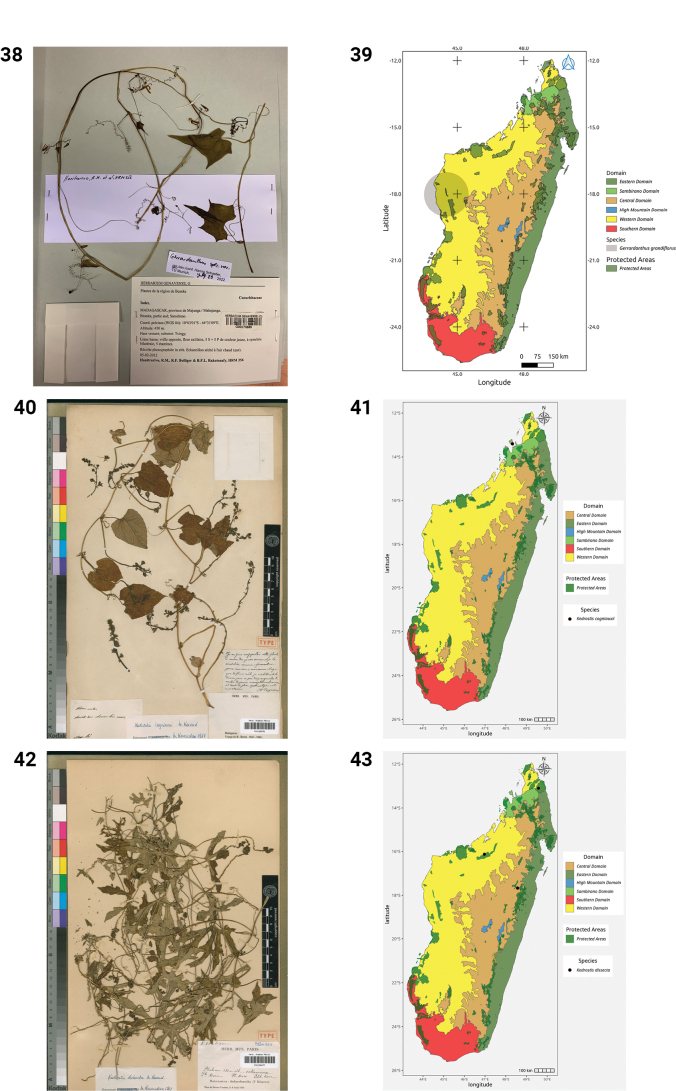
**38.***Gerrardanthus grandiflorus*, Beanka, *R.M. Hanitrarivo et al. 256* (G). **39.** Distribution of *Gerrardanthus grandiflorus*. **40.***Kedrostis cogniauxii*, Holotype, Nossi Be, *L.H. Boivin s.n.* (P). **41.** Distribution of *Kedrostis cogniauxii*. **42.***Kedrostis dissecta*, Holotype, Ankarafantsika, *Service Forestier Madagascar 60* (P). **43.** Distribution of *Kedrostis dissecta*.

**Taxonomy.** Unresolved.

**IUCN.** Based on IUCN criterion B1, the species would be classified as Endangered (EN). However, we suggest classifying it as Least Concern (LC) based on IUCN criterion B2.

***Kedrostis lanuginosa* Keraudren, Bull. Soc. Bot. France 108: 242 (1961).**

**Holotype.** Madagascar • Ste Marie, partie Sud de l’île, 1 Mar. 1847, *L.H. Boivin 1850* P (P00267763!), our Fig. 46.

**Isotype.** P (P00267761!, P00267762!).

**GenBank information.** No DNA sequences available.

**Distribution.** Endemic to Madagascar, where it has been found in the Northeast and West (Fig. 47).

Western Domain: SAVA region, Sambava, North-East coast, *H. Humbert 24409* (P); Sofia region, in Port-Bergé, Tsiningia, Amberoverobe, *R. Razakamalala et al. 1873* (P).

Eastern Domain: Analanjirofo region, isle of Sainte Marie, *L.H. Boivin 1850* (HOLOTYPE, P).

**Occurrence inside Protected Areas.** Not known from protected areas.

**Ecology and habitat.** Littoral, dry forest, and woodland on dunes, from close to sea level up to 50 m asl., on Quaternary sandy substrates along the coast, sheltered from salt spray (Gautier et al. 2018); flowering Nov.-Mar. (Keraudren 1966).

**Taxonomy.** Unresolved.

**IUCN.** We suggest Critically Endangered (CR) based on IUCN criterion B1.

***Kedrostis laxa* Keraudren, Bull. Soc. Bot. France 108: 242 (1961)**

Fig. 48

**Holotype.** Madagascar • Mahajanga, Namoroka (Ambongo), Dec. 1926, *H. Perrier de la Bâthie 17851* P (P00267757!).

**Isotype.** P (P00267758!, P00267759!).

**GenBank information.** Has been sequenced by the authors (unpubl. data).

**Distribution.** Endemic to Madagascar, where it is found in the North and West (Fig. 49).

Western Domain: Diana region, Ankarana Special Reserve, *J.N. Labat 2794* (P), *M. Bardot-Vaucoulon, 64, 753*, *766* (P), *Malcomber 1829* (MO), *H. Schaefer & M.B. Andriamiharisoa 12, 12A, 13, 35, 35B*, *35C* (TUM, DBEV); Melaky, Namoroka NP, *Bardot-Vaucoulon 1899* (P); Mandevy Tsingy formation, *C. Rakotovao 6046* (P); Tsingy of Namoroka, Ambatomay forest, *Ravelonarivo 4529* (MO, TAN), *R. Decary 15786* (P); Ambongo, *H. Perrier de la Bâthie 1619, 6764, 17851* (P).

iNaturalist observation: https://www.inaturalist.org/observations/68722991.

**Occurrence inside Protected Areas.** Ankarana Special Reserve and Namoroka NP.

**Ecology and habitat.** Moist semi-deciduous and dry deciduous forest, on limestone formations (tsingy), in light and low forest on dale, in mosaic vegetation with stunted trees and xerophytic plants, c. 100–200 m asl., characterised by moderate rainfall (400–1,400 mm/year), a pronounced dry season (4–9 months), high temperature and low annual and daily temperature range (Gautier et al. 2018); flowering Nov.–Dec.

**Taxonomy.** Unresolved.

**Figures 44–49.**
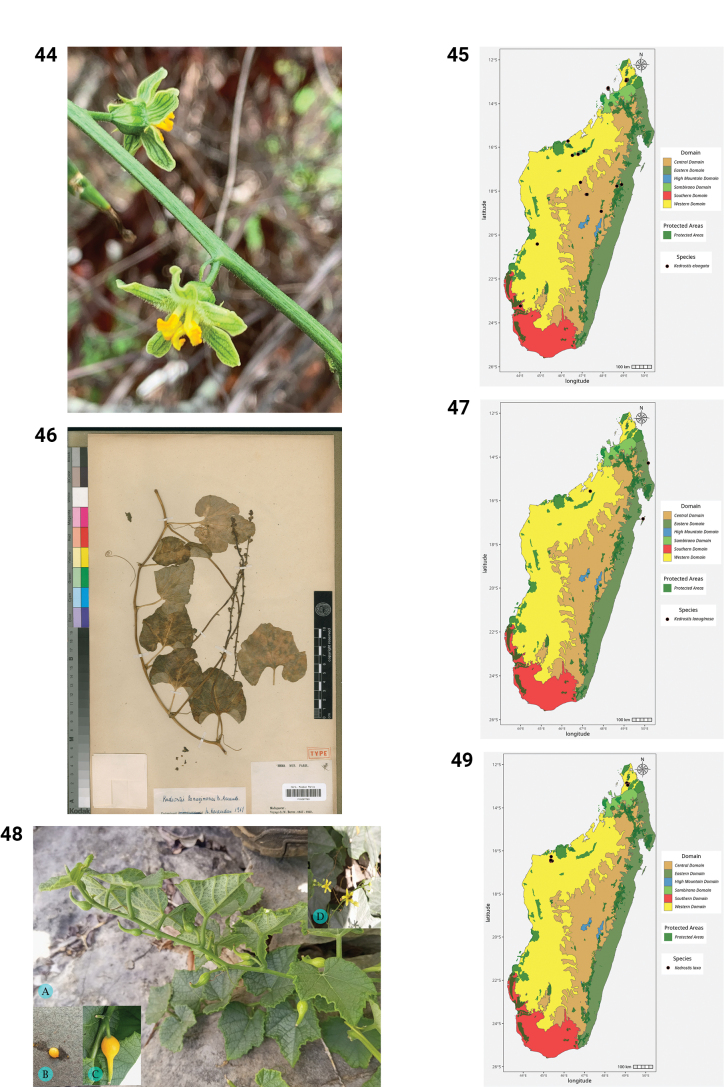
**44.***Kedrostis elongata*, male flower, photo by HS, Ankarafantsika NP, December 2022. **45.** Distribution of *Kedrostis elongata*. **46.***Kedrostis lanuginosa*, Holotype, isle of Sainte Marie, *L.H. Boivin 1850* (P). **47.** Distribution of *Kedrostis lanuginosa*. **48.***Kedrostis laxa*. **A.** Habit; **B.** Seed; **C.** Mature fruit; **D.** Male inflorescence (photos by MBA, Ankarana Special Reserve, December 2023). **49.** Distribution of *Kedrostis laxa*.

**IUCN.** Based on IUCN criterion B1 the species would be classified as Vulnerable (VU). However, based on our field observations we suggest assigning the status Endangered (EN), which better reflects its current condition.

***Kedrostis perrieri* Keraudren, Bull. Soc. Bot. France 108: 242 (1961).**

**Figures 50–55.**
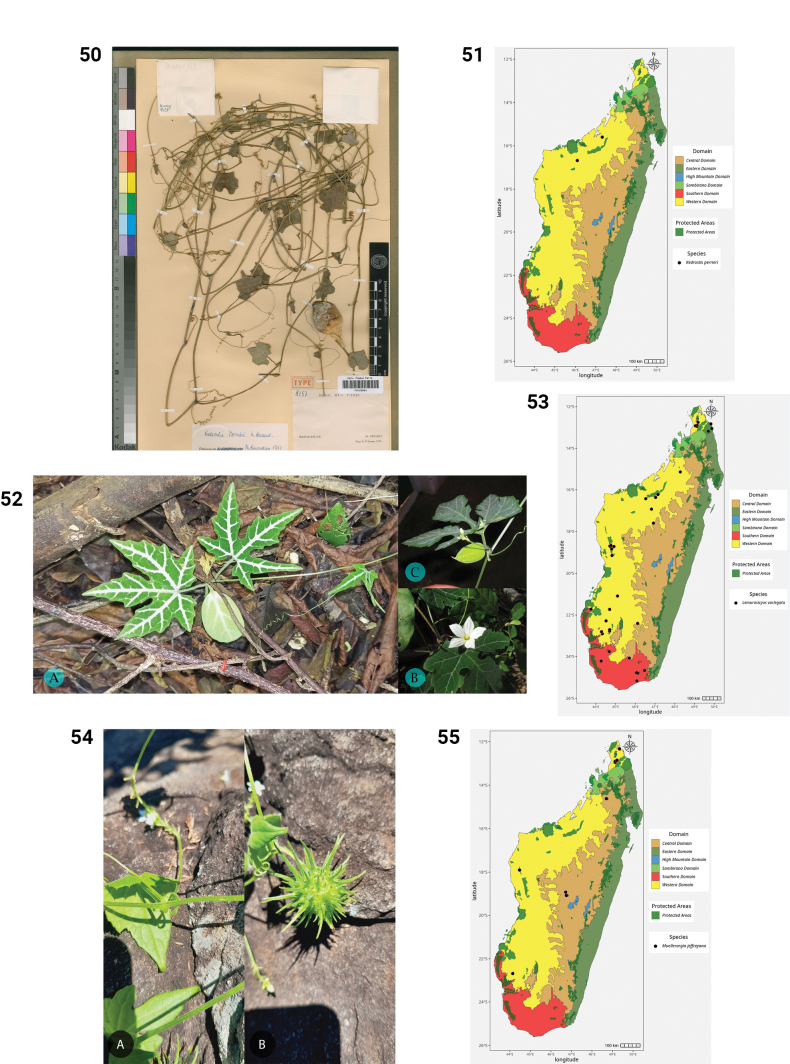
**50.***Kedrostis perrieri*, Holotype, Tsitampiky, *R. Decary 8157* (P). **51.** Distribution of *Kedrostis perrieri*. **52.***Lemurosicyos variegata*. **A.** Habit; **B.** Female flower; **C.** Young fruit (photos A by MBA, Ankarana Special Reserve, December 2023, **B** and **C** by F. Rakotoarivony, Sakaraha-Mahaboboka, February 2011 (fruit), and March 2013 (flower)). **53.** Distribution of *Lemurosicyos variegata*. **54.***Muellerargia jeffreyana*. **A.** Habit; **B.** Fruit (photos **A, B** by F. Rakotonasolo, Antananarivo, Itasy, February 2024). **55.** Distribution of *Muellerargia jeffreyana*.

**Holotype.** Madagascar • Mahajanga, Ouest: partie Nord-Ouest du domaine, Tsitampiky, 28 Sep. 1930, *R. Decary 8157* P (P00135481!), our Fig. 50.

**Isotypes.** P (P00135482!, P00135483!).

**GenBank information.** No DNA sequences available.

**Distribution**: Endemic to Madagascar where it is only known from the West (Fig. 51).

Western Domain: Boeny region, Mahavavy-Kinkony Complexe, Ambongo, *A. Pervillé 589* (P); Tsitampiky, *R. Decary 8157* (HOLOTYPE, P); Sofia region, Bongolava between Port Bergé and Antsijomitondraka, *H. Perrier de la Bâthie 6782* (P).

**Occurrence inside Protected Areas.** Harmonious Protected Landscape Mahavavy Kinkony Wetland Complex.

**Ecology and habitat.** Dry deciduous forest on sandy soil at c. 200 m asl.

**Taxonomy.** Unresolved.

**IUCN.** Based on IUCN criteria B1 and B2 we suggest classifying the species as Critically Endangered (CR) reflecting its highly restricted distribution and the few known occurrence records.

***Lemurosicyos* Keraudren, Bull. Soc. Bot. France 110: 405. 1963 (publ. 1964).**

**Generic type.***Lemurosicyos variegata* (Cogn.) Keraudren.

**Worldwide Distribution.** Madagascar endemic (Schaefer 2020).

***Lemurosicyos variegata* (Cogn.) Keraudren, Bull. Soc. Bot. France 110: 405 (1964)**

Fig. 52

**Basionym.***Luffa variegata* Cogn., *Abh. Naturwiss.* Vereins Bremen 7: 250 (1882).

**Holotype.** Madagascar • Nossy-Bé, *Rutenberg s.n.* (B, destroyed).

**Neotype.** Madagascar • Toliara, Ambatomika, le long du terrain d’aviation d’Ambatomika, route Amboasary à Tsivory, 8 Apr. 1960, *M. Keraudren 1080* P (P00475174!), designated by Keraudren (1963b: 405).

**GenBank information.** DNA sequences of this taxon are available on NCBI published by Kocyan et al. (2007) and Schaefer and Renner (2011): DQ491011, DQ491017, DQ501257, DQ501266, HQ201984.

**Distribution**: Endemic to Madagascar, where it is widespread across the country (Fig. 53).

Western Domain: Diana Region, Ankarana Special Reserve, *M. Keraudren 1688* (P), *M. Bardot-Vaucoulon 1518* (P), *H. Schaefer & M. B. Andriamiharisoa 11, 25* (TUM, DBEV); Nosy Be, Anjiabe, Analabe forest, *R. Rabevohitra et al. 4559* (P); Sava Region, Bekaraoka forest, *L. Gautier 4323* (P); Nosy Sata, Leven, Sata isle, *L. H. Boivin 2572* (P); Betsiboka Region, Morataitra, *H. Perrier de la Bâthie 519* (P); Maevatanana, *R. Decary 19213* (P); Firingalava, *H. Perrier de la Bâthie 519* (P); Melaky, Antsalova, on dry tsingy, *J.F. Villiers 4907* (P); Antsingy, Ambodiriana ford, *J. Léandri 2680* (P); Tsiandro, Antsingy, *J. Léandri 3012*, *3058* (P); Antsalova, *P. Morat 4858* (P); Tsingy of Bemaraha, *D.J. Du, J.-N. Labat & A.M. Couté 891* (P, WAG), *J. Léandri 1027*, *1058* (P), *C.C.H. Jongkind, J.L. Andriantiana & H.E. Razanatsoa 3233* (WAG); Tsingy of Marovato, *S.G. Razafimandimbison 65* (P); Upper Bemarivo, *H. Perrier de la Bâthie 6773* (P); Boeny Region, Tsaramandroso, *M. Peltier 5216* (P); Bevazaha, *RN 33, 1883* (P); Madirovalo dry forest, *H. Perrier de la Bâthie 6786* (P);

iNaturalist observation: https://www.inaturalist.org/observations/1967893).

Southern Domain: Androy region, in Ampandrandava, *A. Seyrig 615*, *615B* (P); Atsimo-Andrefana region, Zombitsy NP, Lambomakandro Forest, *M. Keraudren 1314* (P), *P. Morat 3795bis* (P); Mahaboboka, *M. Peltier 5823* (P), *Randrianarivony, F. Rakotoarivony & Rehary 275* (MO, P), *B. Tefy Andriamihajarivo & F. Rakotoarivony 1868* (P), *Andriamihajarivo 1868* (MO); Anjoho forest, *P. Morat 2489* (P); Mahafaly Plateau, *H. Humbert 29455*, *29508* (P), *B. Descoings 761* (P); Beomby, *J. Bosser 13558* (P), *M. Keraudren 792* (P), *H. Humbert 20288* (P); from Sakamena to Sakoa Valley, *H. Humbert 29432* (P); Tanandava, *J. Bosser 16089*, *16091, 16112* (MO, P); Ankazoabo, *P. Morat 2548, 2517* (P), *J. Bosser 17273, 17704* (P); Andriambe, *P. Morat 3810* (P); Beroroha, Androtsy forest, *R. Razakamalala 5946* (P); Androy region, Ifotaka, *J. Bosser 13562* (P); Bekiria, *M. Keraudren 1012* (BR, P); Ambovombe, clearing area, *R. Decary 8597* (P); Ambatomika, *M. Keraudren 1080* (BR), *M. Keraudren 1080* (P); Menabe Region, 20 km SW of Besely on the road to Tongobory, *M. A. Luckow 4194* (US, WAG); Ihorombe region, on the Ranotsara plain boundaries, *M. Keraudren 328* (P).

**Occurrences inside Protected Areas.** Ankarafantsika NP, Tsingy de Bemaraha NP, Zombitse-Vohibasia NP, Loky Manambato Harmonious Protected Landscape, Nord-Ifotaka Harmonious Protected Landscape, and Ankarana Special Reserve.

**Ecology and habitat.** Moist semi deciduous and dry deciduous forest, on riverbanks, forest edges, roadsides, clearings, and in savanna on red sand, limestone, and alluvial deposits, from 100–1,100 m asl.; flowering Dec.–Apr.

**Taxonomy.***Lemurosicyos variegata* is sister to *Solena heterophylla* and *Borneosicyos simplex* (Kocyan et al. 2007).

**IUCN.** Based on IUCN criterion B1, the species would qualify as Endangered (EN). However, based on B2, we suggest Least Concern (LC).

***Muellerargia* Cogn., Monogr. Phan. 3: 630. 1881.**

**Generic type.***Muellerargia timorensis* Cogn.

**Worldwide Distribution.** Northeastern Australia, New Guinea, and Madagascar (Schaefer 2020).

***Muellerargia jeffreyana* Keraudren, Adansonia, n.s., 5: 423 (1965)**

Fig. 54

**Holotype.** Madagascar • District de Diégo-Suarez, canton d’Anivorano Nord, forêt d’Antenampandrana, km 89,500 de la route d’Ambilobe, calcaires de l’Ankarana, 1 Feb. 1960, *G. Cours 5586* P (P00135452!).

**GenBank information.** DNA sequences published by Schaefer et al. (2009): EU436337, EU436361, EU436387, EU436411.

**Distribution.** Endemic to Madagascar, where it is found in the North, Centre, and West (Fig. 55).

Western Domain: Diana, Montagne des Francais, *M. Bardot-Vaucoulon 1648* (MO, P); Ankarana Special Reserve, *G. Cours 5586* (P), *H. Humbert 32537* (P), *M. Bardot-Vaucoulon 1690* (P); Sofia Region, Betainkankana, Ankaizina forest edge, *J. Bosser 2626* (P); Melaky Region, Beanka (Tsingy), *R. F. Bolliger 165* (P); Atsimo-Andrefana region, Mikoboka, Milenaky, Ambotakebo, *Randrianarivony 1029* (MO, TAN); undergrowth of Anjoho, *P. Morat 2559* (P).

Central Domain: Itasy Region, primary vegetation remnants, trachyte scree, Itasy Lake, *J. Bosser 19189* (P).

**Occurrence inside Protected Areas.** Natural Monument of the Sacred Forest of Alandraza Analavelo, Montagne des Français Protected Harmonious Landscape, Harmonious Protected Landscape of Beanka, and Ankarana Special Reserve.

**Ecology and habitat.** Tropical Forest on limestone hills and plateaus, dry, deciduous forest on clay soil on Eocene limestone, in canyons, at forest edges, remnants of primary forest on trachyte scree, c. 100–1,300 m asl.; flowering Feb.–May (Keraudren 1966).

**Taxonomy.***Muellerargia jeffreyana* is sister to *M. timorensis* from Australia and New Guinea (Schaefer et al. 2009; Telford et al. 2011).

**IUCN.** Based on IUCN criterion B1 we suggest classifying the species as Endangered (EN).

***Peponium* Engl., H.G.A. Engler & K.A.E. Prantl, Nat. Pflanzenfam., Nachtr. 1: 318 (1897).**

**Generic type.***Peponium mackenii* (Naud.) Engl.

**Worldwide Distribution.** Tropical and Southern Africa, Madagascar, and Seychelles (Schaefer 2020).

***Peponium betsiliense* Keraudren, Notul. Syst. (Paris) 16: 148 (1960)**

Fig. 56

**Holotype.** Madagascar • Fianarantsoa, environs d’Ambatofitorahana (Betsileo), au km 300 de la route Tananarive-Fianarantsoa, restes de forêt ombrophile sur argiles latéritiques, 1575 m, Mar. 1955, *H. Humbert 30210* P (P00135362!).

**Isotype.** P (P00135363!, P00135364!).

**GenBank information**. Has been sequenced by the authors (unpubl. data).

**Distribution.** Endemic to Madagascar where it is mainly found in the Southeast (Fig. 57).

Central Domain: Amoron’i Mania region, Ambatofitorahana, *J. Bosser 14903* (MO, P), *M. Keraudren 269, 1167, 1554* (P), *H. Humbert 30210* (P).

Western Domain: Diana region, Sahafary forest, between Diego and Anivorano, *M. Keraudren 1678* (P); Atsimo-Andrefana region, Zombitsy NP, *J. Bosser 19373, 19894, M. Keraudren 457* (P).

**Occurrence in Protected Areas.** Zombitse-Vohibasia NP.

**Ecology and habitat.** Medium altitude moist evergreen and dry deciduous forest on lateritic clay, characterised by high rainfall (>1,200 mm/year), no or brief dry season (< 4 months), and high air humidity (Gautier et al. 2018), flowering Jan.–Apr. (Keraudren 1966).

**Taxonomy.** Unresolved.

**IUCN.** Based on IUCN criterion B1 the species is classified as Critically Endangered (CR).

***Peponium boivinii* (Cogn.) Engl., Nat. Pflanzenfam., Nachtr. 1: 318 (1897)**

Fig. 58

**Basionym.***Peponia boivinii* Cogn. *Monogr. Phan.* 3: 408 (1881).

**Holotype.** Madagascar • Bords du ruisseau d’Andradroite, Nossi-be; Mar. 1851; *L.H. Boivin s.n.* P (P00135366!).

**GenBank information.** No DNA sequences available.

**Distribution.** Endemic to Madagascar where it is found only in the extreme North (Fig. 59).

Western Domain: Sava Region, Daraina, Solaniampilana-Maroadabo forest, *L. Gautier 4519* (G).

Sambirano Domain: Diana region, Nosy-Be, on the streamside of Andradroite, *L.H. Boivin s.n.* (P), Djabalabe, *L.H. Boivin s n.* (P).

**Occurrence inside Protected Areas.** Loky Manambato Harmonious Protected Landscape.

**Ecology and habitat.** Littoral thicket and forest by the sea on sandy soil up to 100 m asl.

**Taxonomy.** Unresolved.

**IUCN.** Based on IUCN criterion B1 the species is classified as Critically Endangered (CR).

***Peponium grandidieri* Keraudren, Notul. Syst. (Paris) 16: 148 (1960)**

Fig. 60

**Holotype.** Madagascar • Mouroundava [Morondava], Mar. 1869, *A. Grandidier s.n*. P (P00135368!).

**Syntype.** P (P00135369!).

**GenBank information.** Has been sequenced by the authors (unpubl. data).

**Distribution.** Endemic to Madagascar, where it was found mainly in the Southwest (Fig. 61).

Western Domain: Boeny region, Ankarafantsika, near Ampijoroa forest station, *M. Keraudren 1203* (P); Atsimo-Andrefana region, Sakaraha, Lambomakandro forest, *J. Bosser 15757* (P), *M. Keraudren 1313* (P); Zombitsy, *P. Morat 3799* (P); Tsaramasao 20 km South of Sakaraha, *J. Bosser 19906* (MO), *P. Morat 3501* (P); undergrowth of Anjohobe, *P. Morat 2600* (P); Menabe, Morondava, *A. Grandidier s.n.* (P).

Sambirano Domain: Diana region, Nosy-Komba, *J. Bosser 14758* (P), *M. Keraudren 1601* (P).

Southern Domain: Anosy region, Ankodida New Protected Area, *H. Schaefer et al. 23A,B* (DBEV, TUM).

**Occurrence inside Protected Areas.** Ankarafantsika NP, Zombitse-Vohibasia NP, and Ankodida Protected Harmonious Landscape.

**Ecology and habitat.** Undergrowth of dry deciduous forests and in remnants of primary forest, c. 200 m asl.

**Taxonomy.** Unresolved.

**IUCN.** We suggest Least Concern (LC) based on IUCN criterion B2.

**Figures 56–61.**
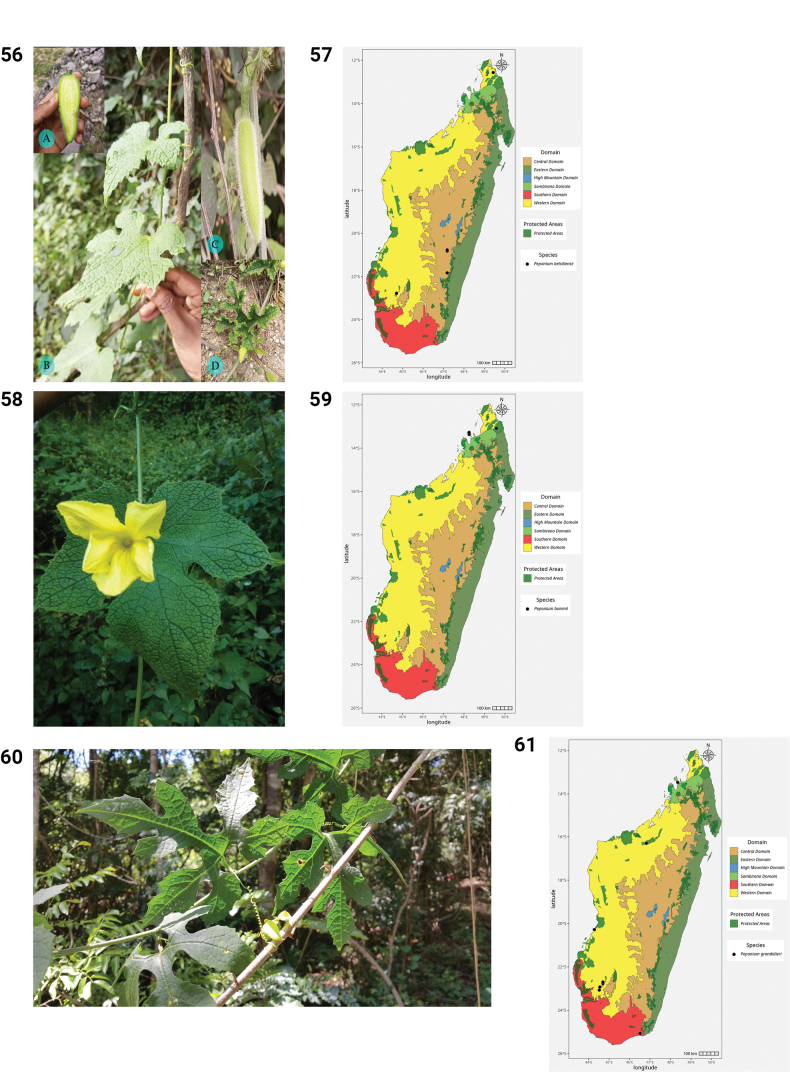
**56.***Peponium betsiliense*. **A.** Fruit, longitudinal section; **B.** Habit; **C.** Young pubescent fruit; **D.** Young leaf with inflorescence (photos by MBA, Ambatofitorahana, November 2023). 57. Distribution of *Peponium betsiliense*. **58.***Peponium boivinii*, plant with female flower (photo by F. Ratianarivo, Tsaratanana Nature Reserve, May 2023). **59.** Distribution of *Peponium boivinii*. **60.***Peponium grandidieri*, habit (photo by S. Cable, Ankarafantsika NP, February 2018). **61.** Distribution of *Peponium grandidieri*.

***Peponium hirtellum* Keraudren, Notul. Syst. (Paris) 16: 147 (1960).**

Fig. 62

Peponium hirtellum Keraudren var. hirtellum, Notul. Syst. (Paris) 16: 147 (1960)

**Holotype.** Madagascar • Toliara, environs de Fort Dauphin, près de Bevilany, 250 m, 14 Sep. 1928, *H. Humbert & C.F. Swingle 5697bis* P (P00135370!).

**Isotypes.** P (P00135371!, P00135372!).

**GenBank information.** No DNA sequences available.

**Peponium hirtellum var. longiracemosum Keraudren Fl. Madagasc. 185: 135 (1966).**

**Holotype.** Madagascar • Toliara, Nord-Ouest de Fort Dauphin, Haute Mananara, près du poste forestier d’Imonty (RN) et du village d’Ambatohabo, dans les restes de végétation sèche sur rochers, avec *Alluaudia*, Pachypodes et *Aloe*, Feb. 1962, *M. Keraudren 1518* P (P00135373!), our Fig. 64.

**Isotypes.** P (P00135374!, P00135375!, P00465122!), MO (MO102597723!).

**Distribution.** Endemic to Madagascar where both varieties of the species have been found only in the Southeast (Figs 63, 65).

Southern Domain: Atsimo-Andrefana region, on the road to Manombo in the North of Toliara, *P. Morat 3841* (P); Anosy region, on the road from Amboasary to Fort Dauphin, the kilometric point no. 434, *J. Bosser 15832* (P), *M. Keraudren 1023, 1480* (P), *M. Keraudren-Aymonin 25052* (P); near Bevilany, *H. Humbert 5697bis* (P); Androy region, between Lavanono and Marovato, *Croat 31574* (MO).

Eastern Domain: Anosy Region, on the mountain between Andohahela and Elakelake, *H. Humbert 13845* (P); Upper Manampanihy Valley, between the Saindro pass and Eminiminy, *H. Humbert 14024* (P); near Imonty Forestry station, a few kilometers from Antotohabo, *M. Keraudren 1518* (MO, P).

**Occurrence inside Protected Areas.**

Peponium hirtellum var. hirtellum: Ankodida Harmonious Protected Landscape.

Peponium hirtellum var. longiracemosum: Andohahela NP.

**Ecology and habitat.**

Peponium hirtellum var. hirtellum grows in dry spiny thicket on sandy soils at the edge of the forest, c. 250 m asl.; flowering Sept.–Feb. (Keraudren 1966).

Peponium hirtellum var. longiracemosum grows in montane ericoid thicket with *Alluaudia*, *Pachypodium*, and *Aloe*, in transition of dry spiny thicket and lowland evergreen moist forest, and in medium altitude moist evergreen forest on lateritic gneiss, c. 600–1,200 m asl.

**Taxonomy.** Unresolved.

**IUCN.**Peponium hirtellum var. hirtellum: We suggest Critically Endangered (CR) based on IUCN criterion B1 for a species with a very restricted area of occurrences.

Peponium hirtellum var. longiracemosum: We suggest Critically Endangered (CR) based on IUCN criterion B1 for a species with a very restricted area of occurrences.

***Peponium humbertii* Keraudren, Notul. Syst. (Paris) 16: 142 (1960).**

**Holotype.** Madagascar • Massif de Marivorahona, au Sud-Ouest de Manambato (Haute Mahavavy du Nord, district d’Ambilobe), vallons supérieurs, parties un peu claires, 2,100 m, 1 Mar. 1951, *H. Humbert & R. Capuron 25808* P (P00135376!), our Fig. 66.

**Isotypes.** P (P00135377!, P00135378!, P00465144!), MO (MO102332705!).

**GenBank information.** Has been sequenced by the authors (unpubl. data).

**Distribution.** Endemic to Madagascar where it was found mainly in the North (Fig. 67).

High Mountain Domain: Diana region, Marivorahona Massif, *H. Humbert 25808* (P, MO).

Western Domain: Sofia region, Bealanana, northeastern Mangidirano, *C. Rakotovao 2361* (P, MO).

Central Domain: Andasibe, Mantadia NP, *H. Schaefer et al. 11*, *21* (TUM, DBEV).

**Occurrence inside Protected Areas.** Natural Resources Reserve of the Marojejy-Anjanaharibe Sud-Tsaratanàna Corridor, northern part.

**Ecology and habitat.** Medium altitude moist evergreen forest, and montane ericoid thicket, climbing on bamboo and *Pandanus*, on roadsides, lake shores, ridge forests, and clearings on gneiss or sandstone, c. 800–2,200 m asl.; flowering in March (Keraudren 1966).

**Taxonomy.** Unresolved.

**IUCN.** We suggest Critically Endangered (CR) based on IUCN criterion B1 for a species with a very restricted area of occurrences.

***Peponium laceratum* Keraudren, Notul. Syst. (Paris) 16: 146 (1960).**

**Holotype.** Madagascar • Alaotra, Bord d’un chemin en forêt d’Antsihanaka, 1 Nov. 1936, *Jardin Botanique Tananarive 2254* P (P00135379!), our Fig. 68.

**GenBank information.** No DNA sequences available.

**Distribution**: Endemic to Madagascar, where it is known only from the Alaotra region and Nosy Mangabe (Fig. 69).

Central Domain: Alaotra Mangoro region, Alaotra, Antsihanaka forest, *Jardin Botanique Tananarive 2254* (P); Ambatosoa region, Maroantsetra, Nosy Mangabe, *M. Keraudren-Aymonin 24512* (P).

**Occurrence inside Protected Areas.** Nosy Mangabe NP.

**Ecology and habitat.** Lowland and medium altitude moist evergreen forest; flowering in March.

**Taxonomy.** Unresolved.

**IUCN**. Due to the small number of collections known, we suggest Data Deficient (DD).

***Peponium perrieri* Keraudren, Notul. Syst. (Paris) 16: 144 (1960).**

Fig. 70

Peponium perrieri Keraudren var. perrieri, Notul. Syst. (Paris) 16: 144 (1960)

**Holotype.** Madagascar • Ouest: haut Bemarivo, sables humides et boisées au bord des rivières, 200 m, Sep. 1907, *H. Perrier de la Bâthie 6770* P (P00135380!).

**Isotype.** P (P00135381!, P00135382!, P00135383!, P00135384!, P00465534!).

**Paratype.** Madagascar • Antananarivo, Manerinerina, Centre: bois des pentes occidentales, à Manerinerina, sur le Tampoketsa, entre l’Ikopa et la Betsiboka, 1,500 m, 1 Dec. 1924, *H. Perrier de la Bâthie 16750* P (P00135386!).

**Figures 62–67.**
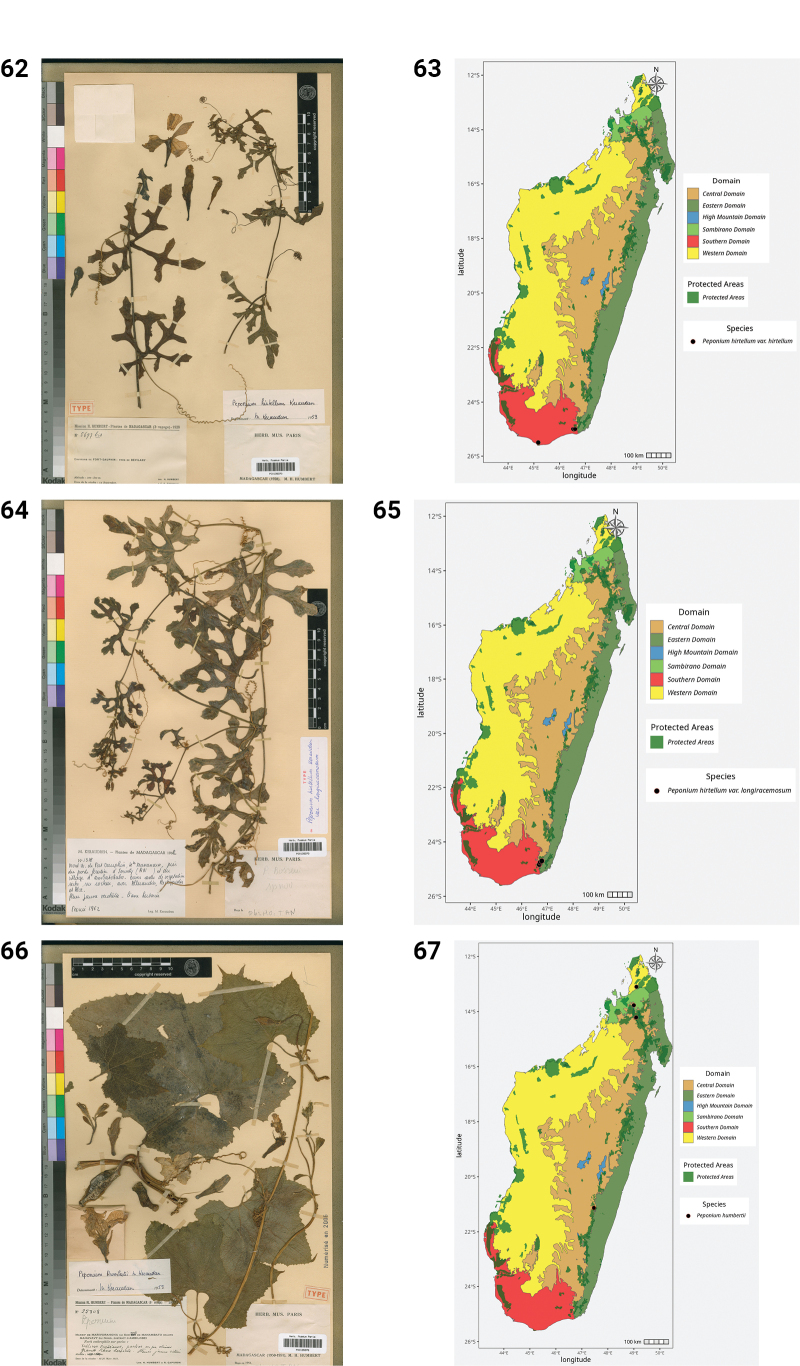
**62.**Peponium hirtellum var. hirtellum, Holotype, Bevilany, *H. Humbert & C.F. Swingle 5697bis* (P). **63.** Distribution of Peponium hirtellum var. hirtellum. **64.**Peponium hirtellum var. longiracemosum, Holotype, Ambatohabo, *M. Keraudren 1518* (P). **65.** Distribution of Peponium hirtellum var. longiracemosum. **66.***Peponium humbertii*, Holotype, Manambato, *H. Humbert & R. Capuron 25808* (P). **67.** Distribution of *Peponium humbertii*.

**Vernacular name.** Takotakokely (Merina).

**GenBank information.** No DNA sequences available.

**Peponium perrieri var. glabrescens Keraudren**

**Holotype.** Madagascar • Centre: bois des pentes occidentales, Tampoketsa d’Antongodrahoza, entre la Betsiboka et la Mahazamba, 1,300 m, 1 Sep. 1922, *H. Perrier de la Bâthie 14841* P (P00135387!), our Fig. 71.

**Isotype.** P (P00135388!).

**Distribution.** Endemic to Madagascar, where it is found mainly in the Centre and West (Figs 72, 73).

Peponium perrieri var. perrieri

Western Domain: Diana region, 45 km southern Diégo, Sahafary forest, *M. Keraudren 1678* (P). Betsiboka region, between Betsiboka and Mahajamba, Antongondrahoza, *H. Perrier de la Bâthie 14841* (P).

Central Domain: Bongolava region, Belobaka, *J. Bosser 17661 (P*); Analamanga, Ankazobe, *J. Bosser 15976* (P); Manerinerina, *H. Perrier de la Bâthie 16750* (P); Alaotra-Mangoro region, Andasibe, Analamazaotra forest, *M. Keraudren-Aymonin 25373 bis* (P); Andranobe forest, road from Ambatondrazaka to Andriamena, *J. Bosser 19838* (P).

Peponium perrieri var. glabrescens

Western Domain: Sofia, region, Upper Bemarivo, riverbank, *H. Perrier de la Bâthie 6770* (P).

**Occurrence inside Protected Areas.** Not known from protected areas.

**Ecology and habitat.**Peponium perrieri var. perrieri has been found in medium altitude moist evergreen forest, on riverbanks and at the forest edge, c. 200–1,600 m asl.; flowering Sept.–Mar. (Keraudren 1966).

Peponium perrieri var. glabrescens has been collected in medium altitude moist evergreen forest on a riverbank; flowering in July (Keraudren 1966).

**Taxonomy.** Unresolved.

**IUCN.**Peponium perrieri var. perrieri: Critically Endangered (CR), based on IUCN criterion B1 for a species with a very restricted area of occurrences.

Peponium perrieri var. glabrescens: Since there is only a single collection known, we suggest Data Deficient (DD).

***Peponium poissonii* Keraudren, Notul. Syst. (Paris) 16: 146 (1960)**

Fig. 74

**Holotype.** Madagascar • Toliara, Ambovombe, forêt claire, 3 Feb. 1931, *R. Decary 8473* P (P00135389!).

**Isotypes.** P (P00135390!, P00135391!).

**GenBank information.** No DNA sequences available.

**Distribution.** Endemic to Madagascar where it has been found only in the South and Southwest (Fig. 75).

Western Domain: Atsimo-Andrefana region, Toliara, *H. Poisson 387* (P); National Road from Isalo-Sakaraha, Zombitsy, *L. Allorge 2098* (P).

Southern Domain: Anosy region, Taolagnaro Prefecture, Fort-Dauphin, Magnambaro Township, Petriky Forest, *R. Gereau 3346* (US); Androy region, Ambovombe, clear forest, *R. Decary 8473* (P).

**Figures 68–73.**
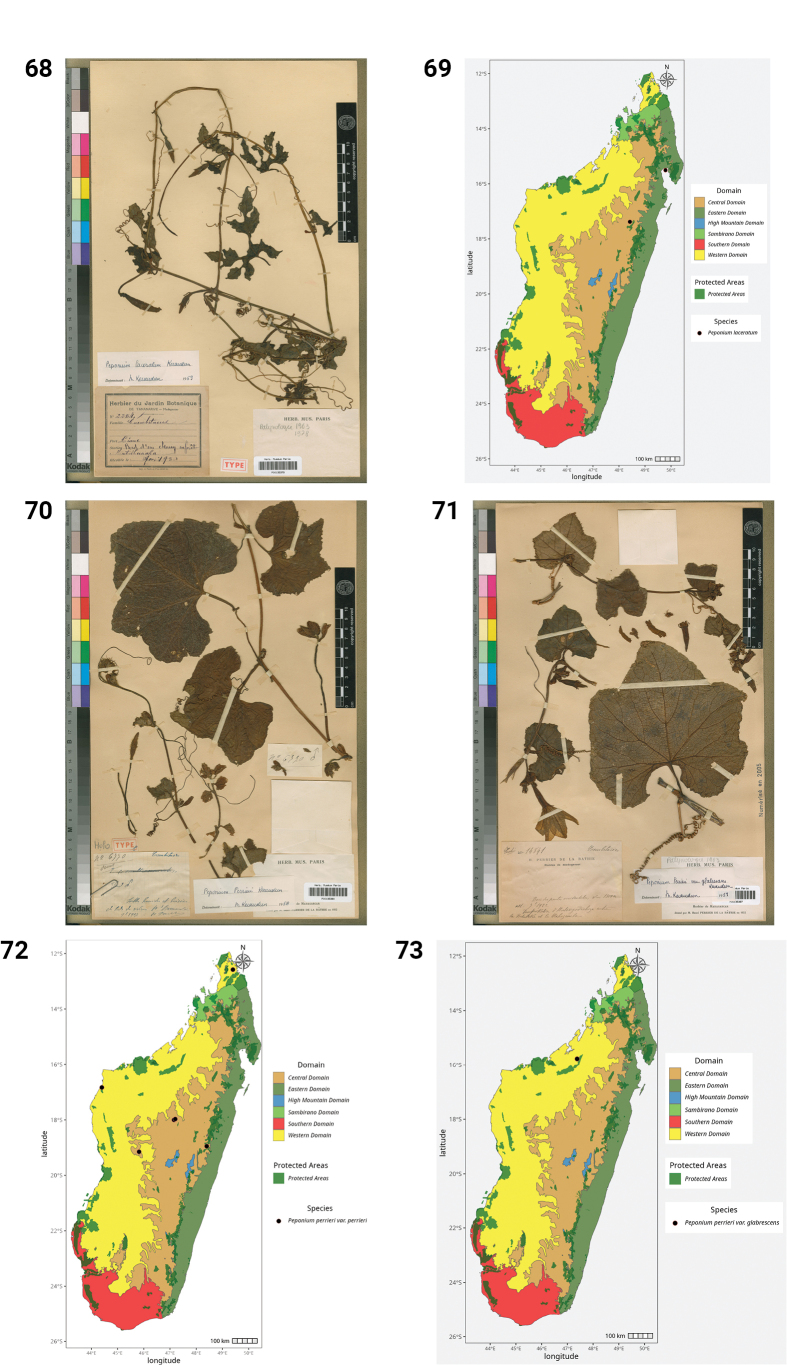
**68.***Peponium laceratum*, Holotype, Antsihanaka forest, *Jardin Botanique Tananarive 2254* (P). **69.** Distribution of *Peponium laceratum*. **70.**Peponium perrieri var. perrieri, Holotype, Bemarivo, *H. Perrier de la Bâthie 6770* (P). **71.**Peponium perrieri var. glabrescens, Holotype, Tampoketsa d’Antongodrahoza, *H. Perrier de la Bâthie 14841* (P). **72.** Distribution of Peponium perrieri var. perrieri. **73.** Distribution of Peponium perrieri var. glabrescens.

**Occurrence inside Protected Areas.** Ranobe PK 32 New Protected Area and Zombitse-Vohibasia NP.

**Ecology and habitat.** Dry deciduous forest and dry spiny thicket, mainly degraded stands on red sand, up to 200 m asl.; flowering in Feb. (Keraudren 1966).

**Taxonomy.** Unresolved.

**IUCN.** Critically Endangered (CR) based on IUCN criterion B1 for a species with a very restricted area of occurrences.

***Peponium racemosum* Keraudren, Notul. Syst. (Paris) 16: 144 (1960).**

**Holotype.** Madagascar • Mahajanga, Firingalava, Ouest: bois de Firingalava, rive droite de l’Ikopa, entre Maevatanana et Andriba, 1 May 1898, *H. Perrier de la Bâthie 618* P (P00135393!), our Fig. 76.

**Isotype.** P (P00135394!, P00135395!).

**GenBank information.** No DNA sequences available.

**Distribution.** Endemic to Madagascar where it has been collected in a few places scattered throughout the country (Fig. 77).

Western Domain: Betsiboka Region, Firingalava, *H. Perrier de la Bâthie 618* (P); Atsimo-Andrefana region, Mahaboboka, Analavelona forest, *Randrianarivony 795* (MO).

Central Domain: Sava region, Antsiranana. Andapa, Ankarongameloka forest, *P. Antilahimena 4694* (P); Andapa, Andranomololo sacred place, *C. Rakotovao 3384* (P).

**Occurrence inside Protected Areas.** Natural Monument of the Sacred Forest of Alandraza Analavelo, and Natural Resource Reserve of the Marojejy-Anjanaharibe Sud-Tsaratanàna Corridor, Northern part.

**Ecology and habitat.** Medium altitude moist evergreen forest, on riverbanks; flowering in March (Keraudren 1966).

**Taxonomy.** Unresolved.

**IUCN.** Critically Endangered (CR) based on IUCN criterion B1 for a species with a very restricted area of occurrences.

***Peponium seyrigii* Keraudren, Notul. Syst. (Paris) 16: 147 (1960)**

Fig. 78

**Holotype.** Madagascar • Ampandrandava, crête Est, endroit très ombragé mais sec, 1100 m, 1 Jan. 1943, *A. Seyrig 456* P (P00135398!).

**Isotype.** P (P00135397!).

**Peponium seyrigii var. linearilobum Keraudren.**

**Holotype.** Madagascar • Analamarina, Forêt d’Analamarina, vallée de l’Hazoroa au Sud de Sakaraha, Forêt tropophile sur sol silicieux gréso-sableux, 300 m, 1 Dec. 1946, *H. Humbert 19613* P (P00135399!).

**GenBank information.** No DNA sequences available.

**Distribution.** Endemic to Madagascar where both taxa are known only from the type collections made in the South and Southwest (Figs 79, 80).

Peponium seyrigii var. seyrigii

Southern Domain: Atsimo-Andrefana region, Ampandrandava *A. Seyrig 456* (P).

Peponium seyrigii var. linearilobum

Atsimo-Andrefana region, Analamarina, Analamarina forest, in Hazoroa Valley, on Taheza Tributary, of Onilahy Basin, Southern Sakaraha, *H. Humbert 19613* (P).

**Figures 74–80.**
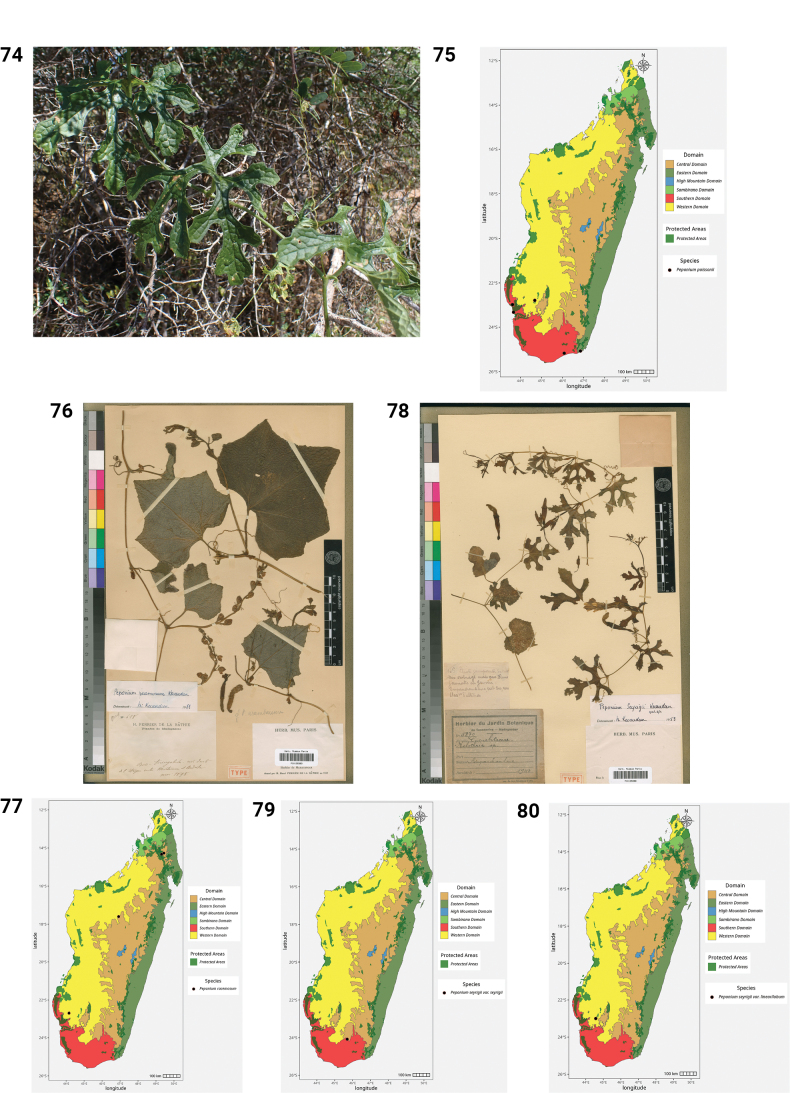
**74.***Peponium poissonii*, habit (photo by L. Ramon, Toliara, April 2024). **75.** Distribution of *Peponium poissonii*. **76.***Peponium racemosum*, Holotype, Firingalava, *H. Perrier de la Bâthie 618* (P). **77.** Distribution of *Peponium racemosum*. **78.**Peponium seyrigii var. seyrigii, Holotype, Ampandrandava, *A. Seyrig 456* (P). **79.** Distribution of Peponium seyrigii var. seyrigii. **80.** Distribution of Peponium seyrigii var. linearilobum.

**Occurrence inside Protected Areas.** Not known from protected areas.

**Ecology and habitat.**Peponium seyrigii var. seyrigii has been described from a shady and dry locality, c. 1,100 m asl.; flowering in Jan. (Keraudren 1966).

Peponium seyrigii var. linearilobum has been described from tropophilous forest on siliceous sandstone soil, c. 300 m asl.; flowering in Dec. (Keraudren 1966).

**Taxonomy.** The two varieties differ in leaf shape (P. seyrigii var. seyrigii with leaf lobes 5 to 6 mm wide at the base vs. P. seyrigii var. linearlobum with leaf lobes no longer than 2 mm wide at the base, (Keraudren 1966) but the taxonomic value of this character is unclear.

**IUCN.** Since both taxa are known from the type collections only and have not been seen for decades, we suggest Data Deficient (DD). They might have become extinct.

***Raphidiocystis* Hook.f., Benth. & Hook., Gen. 1: 828. 1867.**

**Generic type.***Raphidiocystis mannii* Hook.f.

**Worldwide Distribution.** Tropical Africa and Madagascar (Schaefer 2020).

***Raphidiocystis brachypoda* Baker, J. Bot. 20: 113 (1882)**

Fig. 81

**Holotype.** Madagascar, forêt du pays Betsileo, *R. Baron 144* (K).

**Synonyms.***Raphidiocystis sakalavensis* Baker, *J. Linn. Soc., Bot.* 25: 318 (1890).

**GenBank information.** Has been sequenced by the authors (unpubl. data).

**Distribution.** Endemic to Madagascar where it is mainly found in the East (Fig. 82).

High Mountain Domain: SAVA region, Integral Natural Reserve of Marojejy, *F. Rasoavimbahoaka 167* (P); Haute Matsiatra region, Andringitra NP, *Réserves Naturelles Madagascar & Rakotoson 5856 RN* (P).

Western Domain: Sofia region, Befandriana-Matsaborivato, Ampotaka, forest, *Jardin Botanique Tananarive 5612* (P).

Central Domain: Diana region, Montagne d’Ambre NP, *M. Keraudren-Aymonin 25590* (P), *L. Gautier 5179* (P, WAG), *M. Keraudren 1650* (P), *H. Schaefer & M.B. Andriamiharisoa 46*, *46B* (TUM, DBEV), near Diego Viewpoint, *H. Schaefer & M.B. Andriamiharisoa 47* (TUM, DBEV); Analamanga region, Antanamalaza, *Jardin Botanique Tananarive 3546* (P); Mandraka, *C. d’ Alleizette 1064* (P), *M. Keraudren 1132* (P), *M. Keraudren-Aymonin 25402* (P); from Antananarivo to Andasibe, *M. Keraudren-Aymonin 25323* (P), *C. d’ Alleizette 324* (P); Alaotra-Mangoro region, lowerstream Mangoro basin, *H. Perrier de la Bâthie 18279* (P); Moramanga, *P. Morat 4669* (P), *J. Bosser 16622* (P); Ambatovy forest, *P. Antilahimena 6626* (MO, P, TAN); Andasibe NP, *H. Schaefer et al. 2* (TUM, DBEV); Mantadia NP, *H. Schaefer et al. 7,14* (TUM, DBEV); Analamazaotra forest, *M. Keraudren-Aymonin & G.G. Aymonin 25345* (BR), *H.J. Lam 5288* (L), *P.B. Phillipson 2112* (P), *H. Perrier de la Bâthie 6762* (P), *M. Keraudren 1751* (P), *R. Benoist 1173* (P); Vatovavy region, Ranomafana NP, *F. Almeda, 8086* (CAS); Talatakely, *F. Almeda, 8062, 9170* (CAS), *H. Schaefer et al. 102, 108, 109, 119* (DBEV, TUM); Haute Matsiatra region, Ankafina, *A. Rakotozafy 226* (P); Andrambovato, *H. Humbert 28487bis* (P); Anosy region, Integral Natural Reserve of Andohahela, *Rakotomalaza 483* (MO), *H. Humbert 13964* (P).

Eastern Domain: Zahamena NP, *L.M. Randrianjanaka & P. Zafy 209* (BR, P).

**Occurrence inside Protected Areas.** Andringitra NP, Andohahela NP, Mantadia NP, Marojejy NP, Montagne d’Ambre NP, Loky Manambato Harmonious Protected Landscape, Ranomafana NP, Zahamena NP, Ambositra-Vondrozo Forest Corridor Harmonious Protected Landscape, Mangabe-Ranomena-Sahasarotra Natural Resource Reserve, Maromizaha Natural Resource Reserve, and Ankeniheny-Zahamena Corridor Natural Resource Reserve.

**Ecology and habitat.** Undergrowth of medium altitude moist evergreen forest and wet valleys, lake shores, forest edges, on montane ridges, and roadsides, mainly on lateritic soil on gneiss, c. 400–1,300 m asl.; flowering Jul.–Sept. (Keraudren 1966).

**Taxonomy.** Unresolved.

**IUCN.** Based on IUCN criterion B2 we suggest the category Least Concern (LC) for a widely distributed species, occurring in several protected areas.

***Seyrigia* Keraudren, Bull. Soc. Bot. France 107: 299. 1961.**

**Generic type.***Seyrigia gracilis* Keraudren.

**Worldwide Distribution.** Madagascar endemic (Schaefer 2020).

***Seyrigia bosseri* Keraudren, Bull. Soc. Bot. France 109: 101 (1962)**

Fig. 83

**Holotype.** Madagascar • Nord-Ouest de Fort Dauphin, près du poste forestier d’Imonty (R. Nat.) et Ambatohabo (Haut Mananara), dans les restes de végétation xérophytique à Didieracées, *Aloe, Pachypodium*, 17 Feb. 1962, *M. Keraudren 1515* P (P00135495!).

**GenBank information.** Has been sequenced by the authors (unpubl. data).

**Distribution**: Endemic to Madagascar, where it was reported only in the Southeast (Fig. 84).

Central Domain: Anosy region, Andohahela NP, Imonty forestry, Ambatohabo (Upper Mananara), *M. Keraudren 1515* (P), *J. Bosser 15810* (P), *P.J. Rakotomalaza 606* (P).

Southern Domain: Anosy region, Bevoho Northern Amboasary, *Andriamihajarivo 1181* (MO, P, TAN); Androy region, surroundings of Ambovombe, *P. Boiteau 3105* (P).

**Occurrence inside Protected Areas.** Andohahela NP.

**Ecology and habitat.** Dry deciduous forest, and Didiereaceae dry spiny thicket, remnants of xerophilous forests with *Didierea*, *Aloe*, and *Pachypodium* on sandy soils, c. 50–500 m asl.

**Taxonomy.** Unresolved.

**IUCN.** Based on IUCN criterion B1 we suggest Critically Endangered (CR) due to the threat to its habitat by timber extraction and charcoal production.

***Seyrigia gracilis* Keraudren, Bull. Soc. Bot. France 107: 299 (1961)**

Fig. 85

**Holotype.** Madagascar • Toliara, Sud-Ouest, environs de Tuléar, Bush à Famato et Didierea, sables, 1 Aug. 1919, *H. Perrier de la Bâthie 12832* P (P00135498!).

**Isotypes.** P00135499!, P00135500!

**Vernacular name.** Bambola (Masikoro).

**GenBank information.** Has been sequenced by the authors (unpubl. data).

**Distribution.** Endemic to Madagascar, where it was recorded only in the South (Fig. 86).

Western Domain: Atsimo-Andrefana region, Vatolatsaka, *L. Allorge 2127* (P).

Southern Domain: Atsimo-Andrefana region, Androka, Linta Delta, *H. Humbert 5472* (P), *M. Keraudren 894* (P); surroundings of Toliara, *H. Perrier de la Bâthie 6746, 12832* (P), *P.R. Montagnac 70* (P), *B. Descoings 2318* (P), *P.B. Phillipson 4128* (BR), *P. Morat 3027* (P); *M. Keraudren 547, 683* (P), *H. Humbert 2537* (P); Sarodrano, *M. Keraudren 624* (P); Ankilibe, *M. Keraudren 604* (P); Ankililoaka, *H. Humbert 20039* (P); Itampolo, *M. Keraudren 877* (P); Beza Mahafaly Reserve, *P.B. Phillipson 1831* (P); Onilahy Valley, *H. Humbert 2608* (P); Belalanda, *M. Peltier 2549* (P); Fiherenana estuary, *J. Bosser 10597* (P); Ambohimahavelona, *RML 593* (P); Belemboka, *J. Dequaire 27349* (P); Androy region, Antanimora, *H. Humbert 28799* (P); Ambanisariky, *F.R. Fosberg 52496* (US); Saint Augustin, *J. Léandri 3704* (P), *R. Decary 14115* (P); from Ambovombe to Tsihombe, *M. Keraudren 991* (P); Ambovombe, *R. Decary 2638, 3758, 8505, 9179* (P), *J. Bosser 10321, 13578* (P); lake Anongy, *R. Decary 9261* (P); Lefonjavy, *M. Keraudren 974* (P); *G. Aymonin 24830* (P); Menarandra, *J. Léandri 4127* (BR); from Beloha to Lavanono, *T.B. Croat 21529* (P); Behara, *M. Decorse s.n.* (P), *H. Humbert 5657* (P); Ifotaka, *J. Bosser 4052* (P); lower stream Mandrare, *M. Keraudren 1022* (P); Manombo South, *R. Decary 16232* (P); Anarafaly, *M. Keraudren 1019* (P); Berenty, *G. Aymonin 24833* (P), *R. Ranaivojaona et al. 225* (P), *L. Allorge & C. Bourgeois 866* (P), *C. Bourgeois 45* (P); Northern Menarandra, *J. Léandri 4127* (P); from Ampanihy to Ampotaka, *M. Keraudren 912* (P); Tranomaro New Protected Area, *H. Schaefer et al. 18, 19* (DBEV, TUM); Anosy region, Imonty track in Amboasary, middle Mandrare river, *M. Keraudren 1546* (P); Bevilany, *R. Decary 10951* (P).

INaturalist observation: https://www.inaturalist.org/observations/100714500).

**Occurrence inside Protected Areas.** Tranomaro New Protected Area.

**Ecology and habitat.** Dry spiny thicket with *Euphorbia* spp., *Plucheria*, *Alluaudia procera*, and *A. ascendens*, and dry deciduous forest with *Adansonia rubrostipa* and *Delonix floribunda* on calcareous, gneissic, white, and red sand soils or alluvial depositsfrom sea level up to c. 200 m asl., flowering Nov.–Dec.

**Taxonomy.** Unresolved.

**IUCN.** We suggest the category Least Concern (LC) based on IUCN criterion B2 for a species with a wide distribution.

***Seyrigia humbertii* Keraudren, Bull. Soc. Bot. France 107: 299 (1961)**

Fig. 87

**Holotype.** Madagascar • Toliara, Gorges du Fiherenana entre Beantsy et Anjamala, Forêt tropophile et bush xérophile sur rocailles calcaires, 165 m, 1 Jan. 1947, *H. Humbert 19947* P (P00135502!).

**Figures 81–86.**
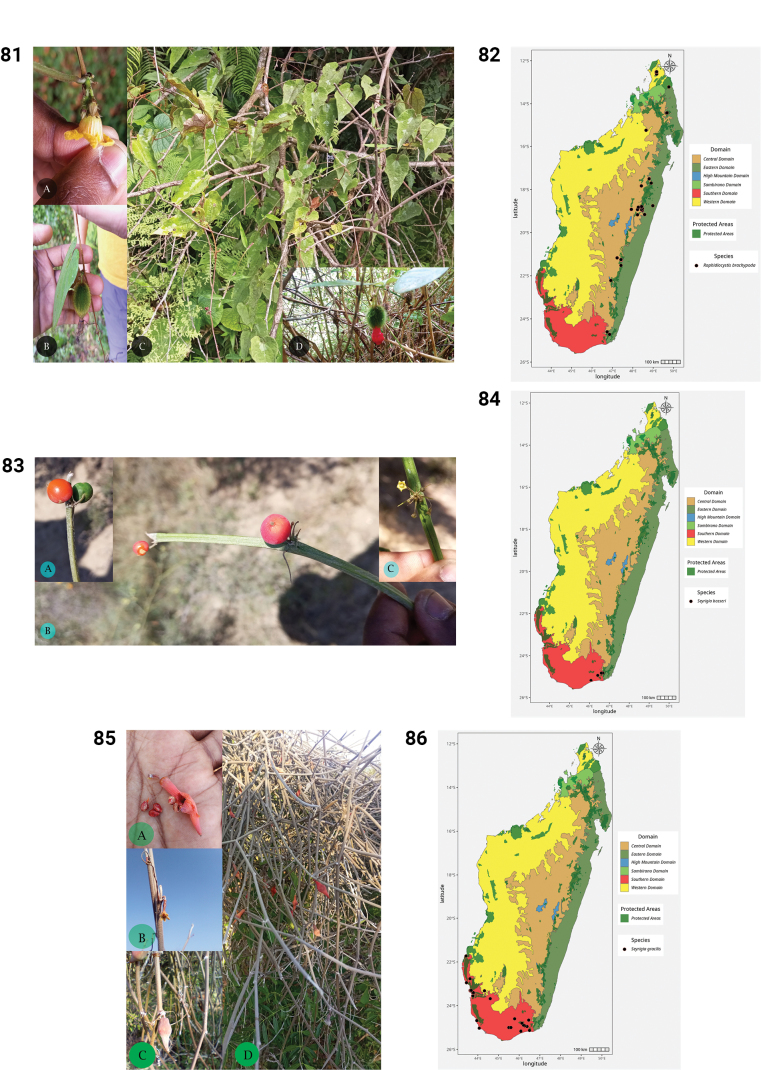
**81.***Raphidiocystis brachypoda*. **A.** Female flower; **B.** Mature fruit; **C.** Habit; **D.** Dehiscent fruit with seeds in red pulp (photos by MBA, Ranomafana NP, October 2022 (**A–C**), and Andasibe NP, December 2021 (**D**)). **82.** Distribution of *Raphidiocystis brachypoda*. **83.***Seyrigia bosseri*. **A.** Mature and immature fruit; **B.** Shoot with ripe fruit; **C.** Male inflorescence (photos by MBA, Andohahela NP, October 2022). **84.** Distribution of *Seyrigia bosseri*. **85.***Seyrigia gracilis*. **A.** Fruit with seeds; **B.** Female flower; **C.** Fruit; **D.** Habit (photos by MBA, Tranomaro New Protected Area, December 2024). **86.** Distribution of *Seyrigia gracilis*.

**Isotype.** P (P00135503!).

**GenBank information.** DNA sequences published by Ayre et al. (2003) and Zhang et al. (2006): AY379781, AY968421, AY968443, AY968457, AY968510, AY968526.

**Distribution.** Endemic to Madagascar, where it is known only from the Southwest (Fig. 88).

Southern Domain: Atsimo-Andrefana region, Ankaloha-IOT, *Ramon 484* (MO); Belalanda, *Ramon 583* (MO); Gorges of the Fiherenana river, *H. Humbert 19947* (P); Fiherenana valley, *W. Rauh 7377* (P), *Humbert 5148* (P), *J. Bosser 13569* (P), *M. Keraudren 766* (P); Miary, *M. Keraudren 640, 671* (P).

**Occurrence inside Protected Areas.** Ranobe New Protected Area.

**Ecology and habitat.** Dry spiny thicket on limestone rocks, from sea level up to c. 200 m asl.

**Taxonomy.***Seyrigia humbertii* forms a clade with *Trochomeriopsis diversifolia* (Kocyan et al. 2007).

**IUCN.** We suggest the category Endangered (EN) based on IUCN criterion B2 for a species with a very restricted distribution.

***Seyrigia marnieri* Keraudren, Bull. Soc. Bot. France 114: 446 (1968).**

**Holotype.** Madagascar • Saint Jean Cap Ferrat, jardin botanique “Les Cèdres”, *M. Keraudren 5* P (P00135504!).

**GenBank information.** Has been sequenced by the authors (unpubl. data).

**Pictures.** colour photographs in Rauh (1995, p. 195, Figs 698–696, 701–702).

**Distribution.** Endemic to Madagascar, only known from the Southwest (Fig. 89).

Southern Domain: Atsimo-Andrefana region, on limestone near Betioky (Rauh 1995); bord du Lac Hanomay, près de lklioky, fourrés xérophiles sur calcaires, *Bosser 191816* (P); 20 km de Sakaraha, route de Ankazoabo, *J. Bosser 19971* (P).

**Occurrence inside Protected Areas.** Not known from protected areas.

**Ecology and habitat.** Dry spiny thicket on limestone.

**Taxonomy.** Unresolved.

**IUCN.** We suggest the category Data Deficient (DD) since the species is poorly known and has not been seen for decades. It might have become extinct.

***Seyrigia multiflora* Keraudren, Bull. Soc. Bot. France 107: 299 (1961)**

Fig. 90

**Holotype.** Madagascar • Bas mandrare, piste d’Amboasary a Ambatomika, dans bush dégradé le long du terrain d’aviation d’Ambatomika, 1 Apr. 1960, *M. Keraudren 1083* P (P00135506!).

**Isotypes.** K (K000242621!), P (P00135507!, P00135508!).

**GenBank information.** Has been sequenced by the authors (unpubl. data).

**Distribution.** Endemic to Madagascar, where it is only found in the South (Fig. 91).

Southern Domain: Atsimo-Andrefana region, Beza Mahafaly Reserve, *P.B. Phillipson 1651* (P); Around Tsimanampetsotsa lake, *J. Léandri 3995* (P); from Ampanihy to Ejeda, *L. Allorge 2319* (P); Bevoalava, *M. Keraudren 1467* (P); from Ampanihy to Ampotaka, *M. Keraudren 917* (P); from Androka to Ampanihy, *M. Keraudren 897* (P), *J. Bosser 13560* (P); Anadabolava, *M. Keraudren 1547* (P), *H. Humbert 12553* (P); Betioky, *H. Humbert 29436* (P); Sakoa, *R. Decary 15953* (P); Anosy region, Ambatomika, *M. Keraudren 1083* (K, P); Isomony, *H. Humbert 12978* (P); Vohipolaka, *H. Humbert 11619* (P); Tranomaro New Protected Area, *H. Schaefer et al. 17* (DBEV, TUM), Tanambao Morafeno, *H. Schaefer et al. 2A,B, 8, 12A,B* (DBEV, TUM); Androy Region, Sihanadahy, from Beloha to Ambovombe, *M. Keraudren 941* (P).

Central Domain: Androy region, East ridge, between Bekily to Tsivory, rocks, half-shade, *A. Seyrig 344B* (P).

**Occurrence inside Protected Areas.** Tranomaro New Protected Area, Andohahela NP, Tsimanampesotse NP, and Bezà-Mahafaly Special Reserve.

**Ecology and habitat.** Dry spiny thicket and dry deciduous and transitional forest with *Euphorbia* and *Adansonia fony* in sandy, calcareous, and rocky soils with gneiss, c. 50–1,100 m asl.; flowering Oct.-Mar. (Keraudren 1966).

**Taxonomy.** Unresolved.

**IUCN.** We suggest the category Least Concern (LC) based on IUCN criterion B2 for a species with a wide distribution.

***Seyrigia napifera* Rauh, Succ. & Xeroph. Pl. Madagascar 2: 193 (1998).**

**Holotype.** Madagascar • 60 km west of Tolanaro, Sept. 1987, *Rauh 68582* (HEID!).

**GenBank information.** No DNA sequences are available for this taxon.

**Pictures.** colour photographs in Rauh (1995, p. 195, figs 703-705).

**Distribution.** Endemic of Madagascar where it is known only from the Southeast (Fig. 92).

Southern Domain: near Tolagnaro (Fort Dauphin) in the Southeast, *Rauh 68582* (HEID).

Central Domain: Anosy region, Integral Natural Reserve, plot no. 2, on the track to Ambatohabo, bush xerophytic, *M. Randriambololona et al. 82* (BR, WAG, P).

**Occurrence inside Protected Areas.** Andohahela NP.

**Ecology and habitat.** In *Alluaudia* bush on sandy soil.

**Taxonomy.** Perhaps closely related to *S. humbertii* with which it shares densely white hairy shoots (Rauh 1995).

**IUCN.** Critically Endangered (CR) according to Rauh (1995).

***Trochomeriopsis* Cogn, Monogr. Phan. 3: 661. 1881.**

**Generic type.***Trochomeriopsis diversifolia* Cogn.

**Worldwide Distribution.** Madagascar endemic (Schaefer 2020).

***Trochomeriopsis diversifolia* Cogn., Monogr. Phan. 3: 661 (1881)**

Fig. 93

**Vernacular name.** Voatangonala (South).

**Holotype.** Madagascar • Port Leven, 1849, *L.H. Boivin 2573* P (P00135491!).

**GenBank information.** DNA sequences published by Kocyan et al. (2007): DQ535859, DQ536607, DQ536746, DQ536878.

**Distribution.** Endemic to Madagascar where it is widespread in the lower regions of the West and South (Fig. 94).

**Figures 87–92.**
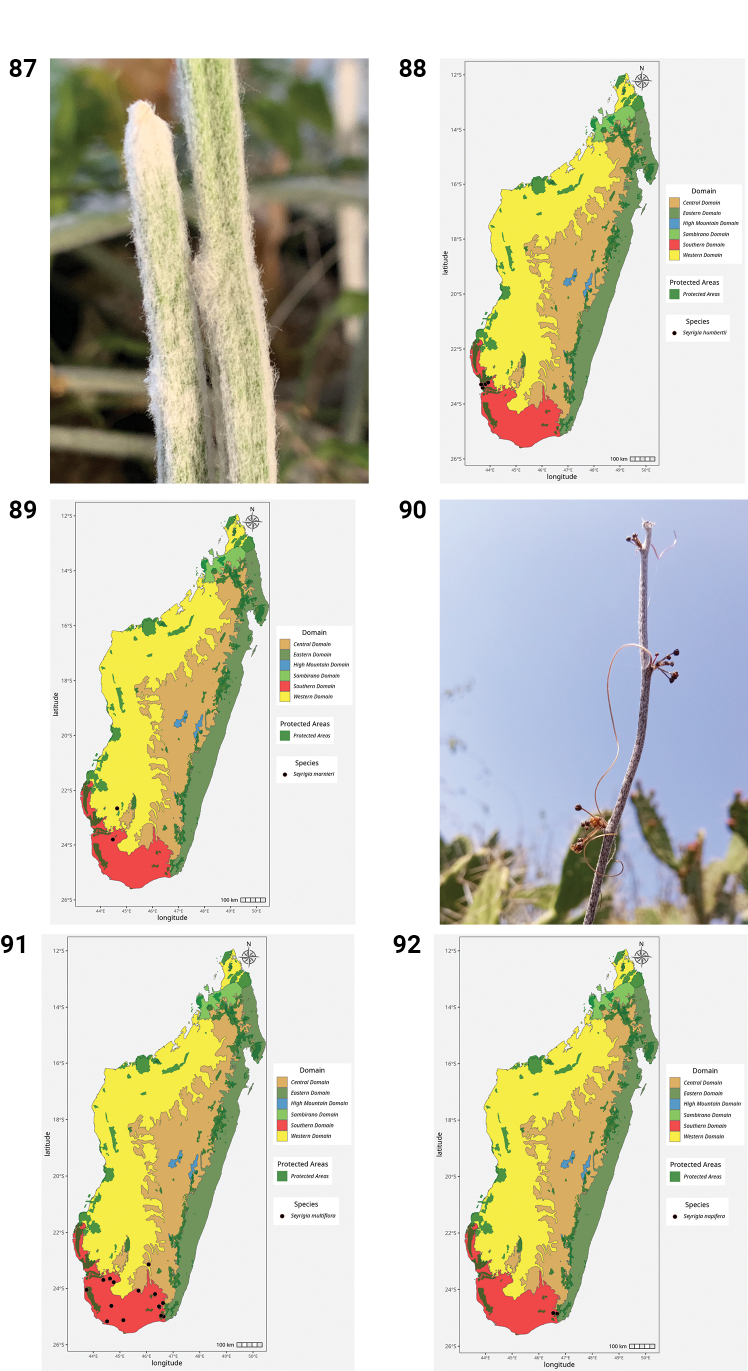
**87.***Seyrigia humbertii*, shoot covered in whitish pubescence (photo by HS, cultivated in Freising, Germany, September 2024). **88.** Distribution of *Seyrigia humbertii*. **89.** Distribution of *Seyrigia marnieri*. **90.***Seyrigia multiflora*, shoot with male inflorescence (photo by MBA, Andohahela NP, October 2022). **91.** Distribution of *Seyrigia multiflora*. **92.** Distribution of *Seyrigia napifera*.

Western Domain: Diana region, Vovo village, *D. K. Harder et al. 1699* (P); Montagne des Français, *A.M. Homolle 319* (P), *M. Bardot-Vaucoulon 1627* (P), *H. Perrier de la Bâthie 16199* (P), *H. Schaefer & M.B. Andriamiharisoa 3* (TUM, DBEV); Mangakoa, *G. Aymonin 25614* (P); Ramena, *J.B. Leopold 14* (P); Analamera, *H. Humbert 19120* (P); Ambodivahibe, Ampasindava, *Ratovoson 985* (CNARP, TAN, MO, P), *R. Ramananjanahary 310* (P); Ankarana Special Reserve, *C.C.H. Jongkind & S. Rapanarivo 980* (BR, P, WAG, US), *H. Humbert 19075 bis* (P), *M. Bardot Vaucoulon 45, 444, 474, 714* (P), *H.Schaefer & M.B. Andriamiharisoa 10, 14,14B* (TUM, DBEV), *L. Allorge 2758*, *2759* (P); Antsisikala, *J.B. Leopold 80* (P); Orangea, *G. Aymonin 25485*, *25474* (P); Beaomby, *J. Bosser 20190* (P); Irodo, *C. Hongwa 243243* (P); Sava Region, Port Leven, *J. Vesco s.n.* (P), *L.H. Boivin 2573* (P).

Sambirano Domain: Diana region, Integral Natural Reserve (RNI) no. 6 Lokobe, *C.R. Birkinshaw 181* (P); Nosy-Be, *L.H. Boivin s. n.* (P); Boeny Region, near the sea, *M. Keraudren 1710* (K, P); Ampijoroa, *M. Keraudren 1250, 1251* (P); Ankarafantsika NP, *H. Schaefer et al. 34, 44* (TUM, DBEV), *H. Perrier de la Bâthie 6736, 13835*, *14691, 15869* (P), *A.H. Gentry 62119* (P); Maevarano, *H. Perrier de la Bâthie 12101* (P); around Marovoay, *H. Perrier de la Bâthie 17961* (P); Betsiboka region, Antsiafabositra, *N.M. Andrianjafy 1051* (P); Mojeramanga, *H. Perrier de la Bâthie 6732* (P); Vilanandro, *H. Perrier de la Bâthie 6735* (P); Sofia region, Mampikony, *R. Decary 14427* (P).

INaturalist observations: https://www.inaturalist.org/observations/8292193 and https://www.inaturalist.org/observations/66955435.

Central Domain: Ramena, Ankorikakely, *F. Ratovoson 981* (P).

Southern Domain: Atsimo-Andrafana region, along Onilahy River, *M. Keraudren 727* (P); Anadabolava, *M. Keraudren 1085, 1535* (P); Anakao, *L. Allorge 2277* (P); Andoharano, *P. Montagnac 47* (P); Bevovo, *J. Léandri 4040* (P); surroundings of Toliara, *M. Keraudren 546* (P); Eastern Mahabo, *G. Aymonin 25563*, *25563*bis (P); Fiherenana, *M. Keraudren 767* (P); Miary, *M. Keraudren 639* (BR, P); Itambono Corridor, *P.B. Phillipson & S. Rabesihanaka 3137* (BR, MO, P, US, WAG); Lambomakandro, *M. Keraudren 1310, 1317* (P); Tsefanoka, *A. Randrianasolo & R. Ranaivojaona R. 1465* (BR, P); Ankilimalinike, *P.B. Phillipson 5946* (MO, P); Mahafaly Plateau, *M. Keraudren 874* (P); Belalanda, Ranobe, *M. Andrianjafy 1831* (P); Eastern Andrevo, *R. Ranaivojaona et al. 1447* (BR, P); Tongobory, *T.B. Croat 31186* (P); Tsivonoa N., *M. Peltier 1373* (P), *J. Bosser 10126* (P); Onilahy Valley, *J. Bosser 13566* (P); Fiherenana Valley, *J. Bosser 13579* (P); Androy region, Ampandrandava, *A. Seyrig 712 B* (P); Betioky, *H. Humbert 29428* (P); Masiaboay, Beara, *R. Randrianaivo 1025* (P); between Tranoroa and Beloha, *J. Bosser 13576* (P); southern Tranoroa, *M. Keraudren 924* (P); Ejeda, *Service Forestier Madagascar 373 r16* (P); Itampolo, *M. Keraudren 876* (P); 218 km to Toliara, *L. Allorge 2167* (P); Basibasy, Ihotry, *H.N. Manjakahery & J. Razanantsoa 193* (P); Anosy Region, Ampihamy, *H. Humbert 11498* (P); Ifotaka, *J. Bosser 13582* (P); Lower stream Mandrare River, *M. Keraudren 1007* (P); Tranomaro New Protected Area, *H. Schaefer et al. 3A,B* (DBEV, TUM). Menabe region, Ankilizato, *G. Aymonin 25849* (P); from Morondava to Belo, *G. Aymonin 25884* (P).

iNaturalist observations: https://www.inaturalist.org/observations/12948635, https://www.inaturalist.org/observations/14070295, https://www.inaturalist.org/observations/70548360.

**Occurrence inside Protected Areas.** Ambohitr’Antsingy-Montagne des Français Harmonious Protected Landscape, Amoron’i Onilahy Harmonious Protected Landscape, Analamerana Special Reserve, Andrafiamena Andavakoera Harmonious Protected Landscape, Ankarafantsika NP, Ankarana Special Reserve, Mangoky Ihotry Wetlands Complex Harmonious Protected Landscape, Menabe Antimena Harmonious Protected Landscape, Nord-Ifotaka Harmonious Protected Landscape, and Ranobe PK32 New Protected Area.

**Ecology and habitat.** Open dry deciduous forest, dry spiny thickets, moist semi-deciduous, gallery forest, woodland and dunes by the sea on red, white, or brown sands, limestone, alluvial deposits or clay soil and outcropping boulders, from sea level up to c. 700 m asl., flowering Nov.-Jan.

**Uses.** Not known, but leaves eaten by goats in the dry South.

**Taxonomy.** According to molecular data, *T. diversifolia* forms a clade with *Seyrigia humbertii* (Kocyan et al. 2007).

**IUCN.** We suggest the category Least Concern (LC) based on IUCN criterion B2 for a species with a wide distribution.

***Xerosicyos* Humbert, Compt. Rend. Hebd. Séances Acad. Sci. 208: 220. 1939.**

**Generic type.***Xerosicyos danguyi* Humbert.

**Worldwide Distribution.** Madagascar endemic (Schaefer 2020).

***Xerosicyos danguyi* Humbert, Compt. Rend. Hebd. Séances Acad. Sci. 208: 221 (1939)**

Fig. 95

**Holotype.** Madagascar • Toliara, Belalanda, environs de Tuléar-Delta du Fiherenana, lieux sablonneux, dunes, également sur les plateaux calcaires rocailleux, 6 m, 14 Sep. 1924, *H. Humbert & H. Perrier de la Bâthie 2440* P (P00135509!).

**Isotypes.** K (K000272553!, K000272554!)

**GenBank information.** DNA sequences published e.g., by Zhang et al. (2006): AJ235648, AY968388, AY968423, AY968459, AY968479, AY968512.

**Distribution.** Endemic to Madagascar where it is mostly found in the South (Fig. 96).

Western Domain: Ihorombe region, along the road to Ivohibe, *L. Bernardi 11200* (P); Ihosy, *M. Keraudren 335* (P); Analavoky, *J. Léandri 345* (BR), *3443* (P), *3484* (P).

Eastern Domain: Anosy region, Ankodida New Protected Area, Mahavelo Forest, *H. Schaefer et al. 25A,B* (DBEV, TUM).

Southern Domain: Atsimo-Andrefana region, Toliara, *P. Morat 1476* (P), *A.M. Homolle 1587* (P), *R. Decary 16159* (P), *AC d’Alleizette s.n.* (L); Onilahy river, *H. Perrier de la Bâthie 12753* (P); Natural Reserve of Tsimanampetsotsa, *H. Humbert 5289* (P, US), *M. Keraudren 1426* (P); Belalanda, *H. Humbert 2420* (K, P), *H. Manjakahery 111* (MO, P, TAN); Fiherenana Valley, *H. Humbert 5156 ter* (P), *J. Léandri 3696* (P); Belemboka, *J. Dequaire 27341* (P); Songeritelo, *B. Koechlin s.n.* (P); Bevato, *B. Koechlin s.n.* (P); Mangoky, *H. Perrier de la Bâthie 4381* (P); Morombe, Ihosy, *J. & M. Peltier 2662* (P); Betioky, near Analafaly, *L. Sussman 331* (P), *Rapanarivo 214* (MO, BR, P, US, WAG); Ambatry, *M. Peltier 2554* (P); between Anakao and Betioky, *R. Decary 16084* (P); Tongobory, *J. Léandri 3740* (P); Mahaleotse, *S.E. Rakotoarisoa 303* (P); 40 km from Toliara, *M. Keraudren 545* (P); La Table, *M. Keraudren 568* (P); Andranovory, *M. Peltier 2519* (P); on the roadside to Ifaty, *P. Morat 7897* (P); Ambohimahavelona, *Rabarivola M. L. 594* (P); on the road to Sarodrano, *F. Chauvet 345* (BR, P), *G. Grandidier s.n.* (P), *M. Keraudren-Aymonin 24704* (P); St. Augustin Township, *G. Cours 3144* (P); Ampanihy, *H. Humbert & C. Swingle 5551* (K, P, US); from Ampanihy to Beloha, *M. Keraudren 926* (P); Itampolo, *J. Léandri 4104* (P), *P.B. Phillipson et al. 3731* (P); Anosiary, limestone plateau, *J. Peltier 2492* (P); Ambovombe, *J. Bosser 10616* (P); Imanombo-Androy, *M. Decorse s.n.* (P); Ambatomika, *M. Keraudren 1082* (P), Ambatomika, *B. Koechlin s.n.* (P); Behara, *Réserves Naturelles Madagascar-Rakotoson 6608 RN* (P); Tanandava, *M. Keraudren 734* (P); Tranomaro, *H. Schaefer et al. 14, 15* (DBEV, TUM); Anosy region, Andohahela NP, *H. Schaefer et al. 68* (DBEV, TUM), *M.B.A. Randriambololona 82* (WAG); Andehomana, *H. Schaefer et al. 76* (DBEV, TUM); between Ambovombe to Tsihombe, *M. Keraudren 993* (P); Northern Ambovombe on the road to Antanimora, *M. Keraudren-Aymonin 25058* (P), *R. Decary 2731* (P); Ambararata, *R. Decary 2988, 2988bis* (P); Behara, *R. Decary 3234* (P); Antanimora, *R. Decary 9200* (P); road to Ifotaka, *M. Keraudren 1473* (P), Fort Dauphin, Vinanibe dry forest, *N. Dumetz 1301* (P); Fort Dauphin, Antanimora, *R. Decary 4627* (P); Andavaka, Andrahomana, *C. Alluaud 12* (P).

iNaturalist observations: https://www.inaturalist.org/observations/8869856; https://www.inaturalist.org/observations/1968017; https://www.inaturalist.org/observations/2742739; https://www.inaturalist.org/observations/12949398; https://www.inaturalist.org/observations/64028998; https://www.inaturalist.org/observations/105662184; https://www.inaturalist.org/observations/72461191; https://www.inaturalist.org/observations/136852990; https://www.inaturalist.org/observations/141155576; https://www.inaturalist.org/observations/142572038; https://www.inaturalist.org/observations/143142452; https://www.inaturalist.org/observations/143557311; https://www.inaturalist.org/observations/102665883; https://www.inaturalist.org/observations/105481841; https://www.inaturalist.org/observations/30103565; https://www.inaturalist.org/observations/74353724; https://www.inaturalist.org/observations/11069140; https://www.inaturalist.org/observations/66240542; https://www.inaturalist.org/observations/36689701.

**Occurrence inside Protected Areas.** Amoron’i Onilahy Harmonious Protected Landscape, Ankodida Harmonious Protected Landscape, Andohahela NP, Bezà-Mahafaly Special Reserve, Cap Sainte Marie Special Reserve, Ranobe PK32 New Protected Area, Tsimanampesotse NP, and Tsinjoriake Harmonious Protected Landscape, Ankodida Harmonious Protected Landscape, and Tranomaro New Protected Area.

**Ecology and habitat.** Dry deciduous, transitional forest and dry spiny forest, where it grows with *Euphorbia, Grewei grevei, Pachypodium geayi*, and Fabaceae on rocky outcrops, limestone plateaus and hillsides from sea level up to 800 m asl.

**Uses.** Widely grown as an ornamental plant (“silver dollar plant”) and easily reproduced from cuttings.

**Taxonomy.** According to Schaefer et al. (2009)*X. danguyi* is sister to *X. perrieri*.

**IUCN.** We suggest the category Least Concern (LC) based on IUCN criterion B2 for a species with a wide distribution.

***Xerosicyos decaryi* Guillaumin & Keraudren, Notul. Syst. (Paris) 16: 127 (1960)**

Fig. 97

**Holotype.** Madagascar • Mahajanga, Namoroka, District de Soalala, Namoroka, reserve n°8, 17 Sep. 1940, *R. Decary 15791* P (P00135510!).

**Isotype.** P (P00135511!).

**GenBank information.** DNA sequence published by Schaefer and Renner (2011): HQ202007.

**Distribution.** Endemic to Madagascar where it is restricted to the South and Southwest (Fig. 98).

Western Domain: Melaky region, Bemaraha Tsingy, *H. Perrier de la Bâthie 2228* (P); Bekopaka, *P. Morat 81*, *777* (P); Boeny Region, Namoroka, *R. Decary 15791* (P); Atsimo-Andrefana region, Zombitsy, *M. Keraudren 460* (P), *L. Allorge 2109* (P), *H. Schaefer et al. 79*, *93* (DBEV, TUM); Ihorombe region, Ihosy District, from Ambalavao to Ihosy, *M. Keraudren 1091* (P).

Southern Domain: Atsimo-Andrefana region, Toliara, *M. Keraudren 526* (P); Anjamala, *M. Keraudren 760, 1353* (P); Beza Mahafaly Reserve, *P.B. Phillipson 2531*, *3686* (P); Androy region, Behompy, Mihary, Behompy, *G. Cours 3140* (P); Anosy region, Bekiria, *M. Keraudren 1021* (P); Andohahela NP, *H. Schaefer et al. 65* (DBEV, TUM).

iNaturalist observation: https://www.inaturalist.org/observations/38956168.

**Occurrence inside Protected Areas.** Andohahela NP, Namoroka NP, Nord-Ifotaka Harmonious Protected Landscape, Ranobe PK32 New Protected Area, Tsimanampesotse NP, and Zombitse-Vohibasia NP.

**Ecology and habitat.** Dry deciduous forest on both sandy soil and rocky woodland on Jurassic limestone from sea level up to c. 800 m asl.

**Taxonomy.** Unresolved.

**IUCN.** We suggest the category Least Concern (LC) based on IUCN criterion B2 for a species with a wide distribution.

***Xerosicyos hirtellus* (Humbert) H. Schaef. & S.S. Renner, Taxon 60: 135 (2011)**

Fig. 99

**Basionym.***Zygosicyos hirtellus* Humbert, *Bull. Soc. Bot.* France 111: 170 (1945).

**Holotype.** Madagascar • Anadabolava, Vallée moyenne du Mandrare près d’Anadabolava, forêt sèche; 225 m, 1 Dec. 1933, *H. Humbert 12439bis* P (P00135518!).

**Isotype.** P (P00135518!).

**GenBank information.** Has been sequenced by the authors (unpubl. data).

**Distribution.** Endemic to Madagascar where it is known only from the type material from the Mandrare valley (Fig. 100).

Southern Domain: Androy region, Anadabolava, Middle Mandrare Valley near Anadabolava, dry forest, *H. Humbert 12439bis* (P).

**Occurrence inside Protected Areas.** Not known from protected areas.

**Ecology and habitat.** Dry deciduous forest, c. 200 m asl.

**Taxonomy.** Morphological very close to *X. tripartitus* and perhaps just a variety of that species.

**IUCN.** We suggest Critically Endangered (CR) based on criterion B1. The species is likely to face threats from illegal trade.

**Figures 93–98.**
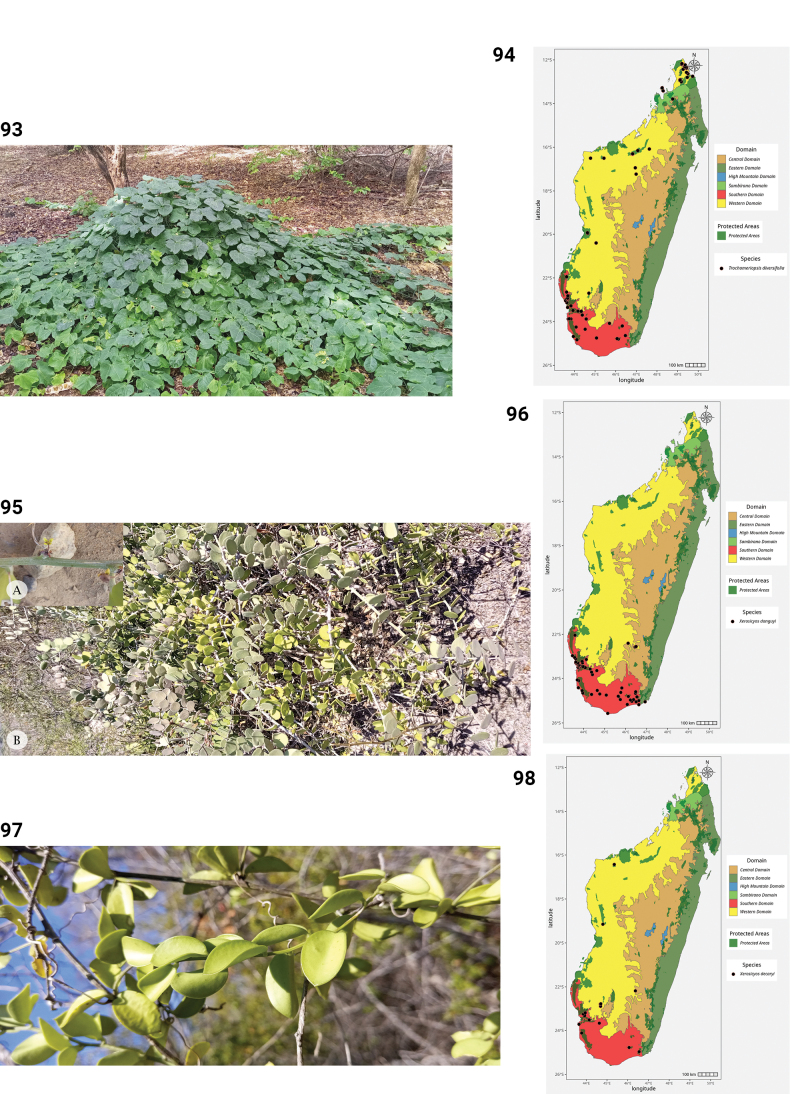
**93.***Trochomeriopsis diversifolia*, habit (photo by MBA, Ankarana Special Reserve, December 2023). **94.** Distribution of *Trochomeriopsis diversifolia*. **95.***Xerosicyos danguyi*. **A.** Flower; **B.** Habit (photos by MBA, Andohahela NP, October 2022). **96.** Distribution of *Xerosicyos danguyi*. **97.***Xerosicyos decaryi*, habit (photo by MBA, Zombitsy-Vohibasia NP, October 2022). **98.** Distribution of *Xerosicyos decaryi*.

***Xerosicyos perrieri* Humbert, Compt. Rend. Hebd. Séances Acad. Sci. 208: 221 (1939)**

Fig. 101

**Holotype.** Madagascar • Toliara, Ampanihy, Sud-Ouest: Environs d’Ampanihy, Bush à Euphorbes et Didierea, 1 Jul. 1932, *H. Perrier de la Bâthie 18659* P (P00135512!).

**Isotypes.** P (P00135514!), K (K000272576!).

**Syntypes.** Madagascar • ♀, Toliara, Benenitra, Ouest: Environs de Benenitra (Onilahy), Broussailles à xérophytes, 600 m, 1 Jul. 1919, *H. Perrier de la Bâthie 2707* P (P00135516!); Mahajanga, Ouest: bois sablonneux près de Maintirano, *H. Perrier de la Bâthie 1994* P (P00135517!).

**GenBank information.** DNA sequences of the species have been published by Schaefer et al. (2009): EU436344, EU436372, and EU436421.

**Distribution**: Endemic to Madagascar, where it is restricted to the South (Fig. 102).

Western Domain: Ihorombe region, between Ihosy and Ivohibe, Tsimirimbo, *J. Léandri 3467* (P); Ihosy, *B. Descoings 3664* (P); Zazafotsy, *M. Keraudren 319*, *1264* (P); Melaky region, near Maintirano, *H. Perrier de la Bâthie 1994* (P).

Southern Domain: Atsimo-Andrefana region, 40 km from Toliara, *M. Keraudren 518* (P); Ampanihy, *H. Perrier de la Bâthie 4380, 18659* (P, K); Bealoka, *L. Allorge 691, 692* (P); Benenitra, *H. Perrier de la Bâthie 12707* (P); Beroroha, *H. Humbert 11286* (P); Beza Mahafaly Reserve, *P.B. Phillipson 2531, 3686* (BR, WAG), *M. Keraudren 899* (P); Sakoa Valley, *R. Decary 15947* (P); Fiherenana, Miary, *M. Keraudren 649* (P), *B. Descoings 1034* (P); Zombitsy, *J. Léandri 3624* (P), *H. Schaefer et al. 94, 95* (DBEV, TUM); Sakaraha, *H. Schaefer et al. 78* (DBEV, TUM); Androy region, Berenty *C.M. Hladik 1255* (P), *P.B. Phillipson 2316* (BR, WAG), *M. Keraudren-Aymonin & G.G. Aymonin 24837, 24838* (BR, P); Ambovombe, *J. Bosser 10384* (P), *R. Decary 2890* (P), *M. Keraudren 972* (BR, P); Ampandrandava, *A. Seyrig 356* (P); Antanimora, *R. Decary 4613* (K); Anosy region, Fort Dauphin, *R. Decary 4266* (P); Imangiry, *R. Decary 8938* (P); Imanombo, *M. Decorse s.n.* (P); Tranomaro New Protected Area, *H. Schaefer et al. 10, 16A,B* (DBEV, TUM); Amboasary, *X. Aubriot 56* (P); Bekiria, Ifotaka, *M. Keraudren 1020* (P); between Amboasary and Fort-Dauphin, *M. Keraudren-Aymonin 24859* (P); Imonty, *J. Léandri 4201, 4564* (P); Menabe Region, Mahabo, *J. Dequaire 27145* (P), *J. Bosser s.n.* (P).

iNaturalist observations: https://www.inaturalist.org/observations/1339915, https://www.inaturalist.org/observations/12949026.

**Occurrence inside Protected Areas.** Mangoky Ihotry Wetlands Complex Harmonious Protected Landscape, Nord-Ifotaka Harmonious Protected Landscape, and Zombitse-Vohibasia NP.

**Ecology and habitat.** Moist semi-deciduous and dry deciduous forest, and dry spiny thicket, and coppice associated with *Euphorbia*, *Alluaudia procera*, and *Baudouinia rouxevillei* on sand and sandstone, gneissic rocks, and alluvial deposits from sea level up to c. 800 m asl.; flowering in July (Keraudren 1966).

**Taxonomy.** According to Schaefer et al. (2009), *X. perrieri* is sister to *X. danguyi*.

**IUCN.** We suggest the category Least Concern (LC) based on IUCN criterion B2.

***Xerosicyos pubescens* Keraudren, Bull. Soc. Bot. France 111: 182 (1965)**

Fig. 103

**Basionym.***Zygosicyos pubescens* (Keraudren) G.D. Rowley, *Cact. Succ. J.* (Los Angeles) 74: 273 (2002 publ. 2003).

**Vernacular name.** Betoboky (Antandroy).

**Holotype.** Madagascar • Jardin Botanique de Tananarive: rocaille malgache de Tsimbazaza, origine: Fort Dauphin, peut-être Imonty, May 1960, *M. Keraudren 1168* P (P00057057!).

**GenBank information.** DNA sequences published by Schaefer et al. (2009): DQ535873, DQ536750, DQ535775, DQ536611, DQ535882.

**Distribution.** Endemic to Madagascar, where it has been found only in a small area of the Southeast (Rauh 1996) in five small populations (Fig. 104).

Southern Domain: Tolagnaro, *M. Teissier 193* (P); Andrahomana, *W. Rauh s.n.* (P); Ankodida New Protected Area, on gneissic rocks, with *Moringa drouhardii, Commiphora* spp., and *Alluadia procera, H. Schaefer et al. 21A,B, 22, 24* (DBEV, TUM).

**Occurrence inside Protected Areas.** Ankodida Harmonious Protected Landscape.

**Ecology and habitat.** In rocky and slightly shaded areas of dry spiny thickets and dry deciduous forests with *Alluaudia procera* and *A. ascendens* and *Euphorbia* sp., growing on syenitis, c. 50–150 m asl. (Rauh 1996); flowers and fruits Nov.-Mar.

**Uses.** The species is grown as an ornamental caudex plant in the Northern Hemisphere and wild-collected material of dubious origin is sold online. *Xerosicyos pubescens* was included in CITES Appendix II (https://cites.org/sites/default/files/eng/app/2015/E-Appendices-2015-02-05.pdf), following a proposal by the Madagascar CITES Management Authority on March 25, 2010 (CoP15 Prop. 26) under the synonym name *Zygosicyos pubescens*.

**Taxonomy.** According to Schaefer et al. (2009), *X. pubescens* is sister to *X. tripartitus*.

**IUCN.** Critically Endangered (CR) since the species grows at elevations up to 150 m asl. and is highly threatened by illegal collecting, slash-and-burn practices, charcoal and fuel wood extraction, overgrazing, the conversion of forest to agricultural fields, mica mining inside and around the protected areas where it grows (pers. obs., December 2024).

***Xerosicyos tripartitus* (Humbert) H. Schaef. & S.S. Renner, Taxon 60: 135 (2011)**

Fig. 105

**Basionym.***Zygosicyos tripartitus* Humbert, *Bull. Soc. Bot.* France 111: 168 (1945).

**Syntypes.** Madagascar • Toliara, Anadabolava, Vallée moyenne du Mandrare près d’Anadabolava, 225 m, 1 Dec. 1933, *H. Humbert 12586bis* P (P00135520!, P00135521!); Bassin supérieur du Mandrare (Sud-Est): du col de Vavara à la vallée de la Manambolo, 750 m, Nov. 1928, *H. Humbert 6746* P (P00135524!).

**Vernacular name.** Betoboky (Antandroy).

**GenBank information.** DNA sequences published by Kocyan et al. (2007) and Schaefer and Renner (2011): DQ535732, DQ536615, DQ536755, DQ536886, HQ202010.

**Figures 99–104.**
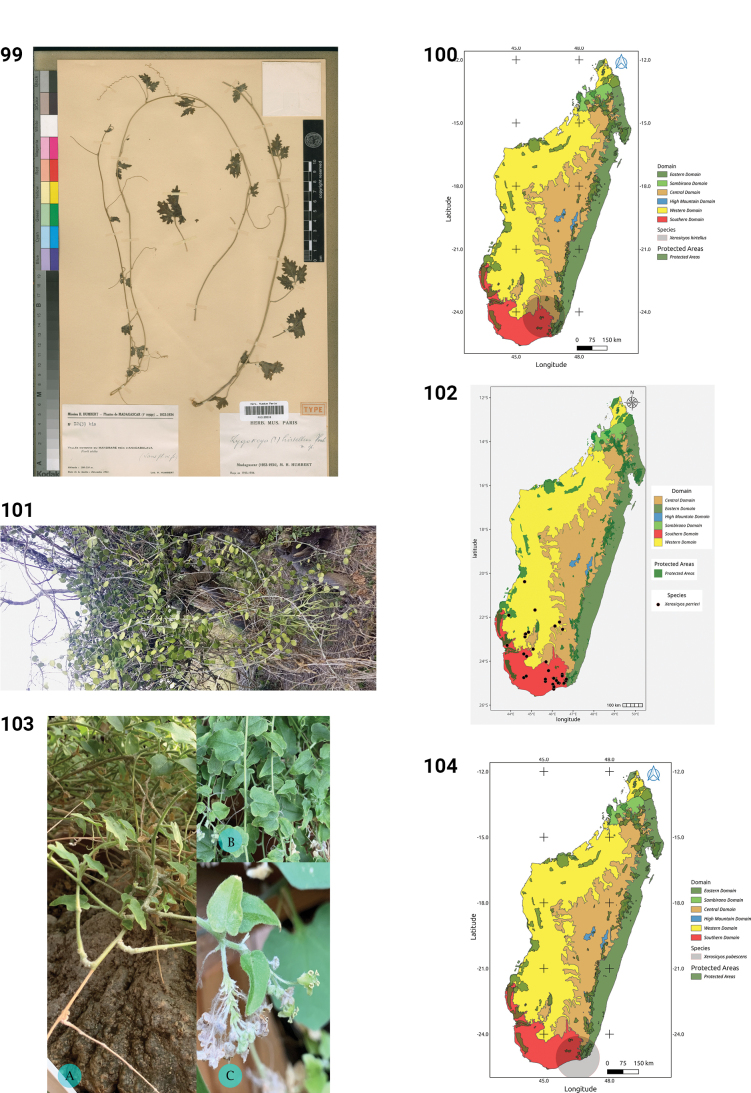
**99.***Xerosicyos hirtellus*, Holotype, Anadabolava, *H. Humbert 12439bis* (P). **100.** Distribution of *Xerosicyos hirtellus*. **101.***Xerosicyos perrieri*, habit (photo by MBA, Zombitsy-Vohibasia NP, October 2022). **102.** Distribution of *Xerosicyos perrieri*. **103.***Xerosicyos pubescens*. **A.** Tuber; **B.** Habit; **C.** Male inflorescence (photos by HS, cultivated in Freising, Germany, September 2024). **104.** Distribution of *Xerosicyos pubescens*.

**Distribution.** Endemic to Madagascar, where it is present in two provinces in the Southeast (Fig. 106).

Southern Domain: Anosy region, Imonty, Mananara Basin, Mandrare Tributary, dry forest, *J. Bosser 15844* (MO), 17047 (P); Middle Mandrare Valley, near Anadabolava, dry forest, *H. Humbert 12586bis* (P); Upper Mandrare Basin (South-East), from Vavara col to Manambolo Valley, on gneiss rocks, *H. Humbert 6746* (P), *J. Bosser 15843, 15844* (P); North-Western Fort-Dauphin, Upper Mananara, near Imonty (Natural Reserve) forestry office, on xerophilous vegetation remnants with *Alluaudia, Pachypodium* and *Aloe, M. Keraudren 1517* (BR, P); Tranomaro New Protected Area, *H. Schaefer et al. 1A,B, 13A,B* (DBEV, TUM).

**Occurrence inside Protected Areas.** Tranomaro New Protected Area.

**Ecology and habitat.** Dry deciduous forest, on gneissic rocks, remnants of xerophytic *Alluaudia, Pachypodium*, and *Aloe*, c. 300 m asl.; flowering in Dec.

**Uses.** The species is grown as an ornamental caudex plant in the Northern Hemisphere, and wild-collected material of dubious origin is sold online. *Xerosicyos tripartitus* was included in CITES Appendix II (https://cites.org/sites/default/files/eng/app/2015/E-Appendices-2015-02-05.pdf), following a proposal by the Madagascar CITES Management Authority on March 25, 2010 (CoP15 Prop. 27) under the synonym name *Zygosicyos tripartitus*.

**Taxonomy.** According to Schaefer et al. (2009), *X. tripartitus* is sister to *X. pubescens*.

**IUCN.** Endangered (EN) since the species was found at elevations of c. 300 m asl. in a very restricted area, making it highly threatened mainly due to illegal collecting, slash-and-burn practices, charcoal and fuel wood extraction, over-grazing, the conversion of the forest to agriculture field, and the presence of mining (mica) in or near the protected areas where it occurs.

***Xerosicyos* sp. nov. North**

Fig. 107

**GenBank information.** Has been sequenced by the authors (unpubl. data).

**Distribution.** Endemic to Madagascar where it is only known from the North (Fig. 108).

Herbarium specimens of this taxon were examined virtually through the GBIF collection by M.B. Andriamiharisoa and physically by H. Schaefer at the Muséum National d’Histoire Naturelle (P) in August 2022.

Western Domain: Antsiranana, District Vohémar, Commune de Daraina, *M. Callmander & A. Be 278* (P); Ampisikinana, Tsaratanana, forêt Ampondrabe, *R. Guittou et al. 191* (P, CNARP, TAN, MO).

**Occurrence inside Protected Areas.** Loky Manambato Harmonious Protected Landscape.

**Ecology and habitat.** Dry deciduous forest, c. 200 m; flowering in Oct.

**Taxonomy.** Unresolved.

**IUCN.** We suggest Critically Endangered (CR) based on criterion B1 and B2.

***Zehneria* Endl., Prodr. Fl. Norfolk. 69. 1833.**

**Generic type.***Zehneria baueriana* Endl.

**Worldwide Distribution.** Old world Tropics and Subtropics (Schaefer 2020).

***Zehneria emirnensis* (Baker) Keraudren, Adansonia, n.s., 4: 333 (1964)**

Fig. 109

**Basionym.***Melothria emirnensis* Baker, *J. Linn. Soc., Bot.* 21: 346 (1884).

**Syntypes.** Madagascar • *Baron 390*, *397*, *2812* (K); *Parker s.n.* (K).

**Synonyms.***Pilogyne emirnensis* (Baker) W.J. de Wilde & Duyfjes, *Blumea* 55: 294 (2010); *Melothria viridis* Zimm., *Cucurbitac.* 2: 181 (1922); *Trochomeria madagascariensis* Baker, *J. Bot.* 20: 113 (1882); *Zehneria madagascariensis* (Baker) C. Jeffrey, *Kew Bull.* 19: 223 (1965), nom. illeg.; *Zehneria viridis* (Zimm.) C. Jeffrey, *Kew Bull.* 15: 371 (1961, publ. 1962).

**GenBank information.** DNA sequences published by Dwivedi et al. (2018): KY523268, KY523327, KY523328, KY523361, KY523429.

**Distribution.***Zehneria emirnensis* is native to Eastern Tanzania and Comoros as well as the more humid parts of Madagascar, from Montagne d’Ambre in the North to the Anosy Mountains in the South (Fig. 110).

High Mountain Domain: Diana region, Tsaratanana Massif, *H. Humbert 18512, 18513* (P), *H. Perrier de la Bâthie 16422* (P); Marivorahona Massif South-Western Manambato, *H. Humbert 25702* (P); Haute Matsiatra region, Andringitra massif, *H. Perrier de la Bâthie 14402* (P); National Road no. 5, Sendrisoa Township, Ambalavao District, *Réserves Naturelles Madagascar-Henri: surveillant 5552 RN* (P); *M. Peltier 2387* (P).

Sambirano Domain: Sambirano Massif, gneiss rocks, shaded, *H. Perrier de la Bâthie 6784* (P).

Central Domain: Analamanga region, Antananarivo, *J.P. Goudot 23* (BR); Ilafy, *C. d’ Alleizette 231*, *802* (P); Manjakatompo primary forest, *M. Keraudren 1265* (P); Manandona, *G. Scott-Elliot 2012* (P); Ankafobe forest, *A. Lehavana 254* (P); Angavokely, *J.M. Hildebrandt 3687* (US); Andrangoloaka, *J.M. Hildebrandt 3688, 3826* (BR, P); Mandraka, *R. Benoist s.n.* (P), *A. Rakotozafy 564* (P), *J. Bosser 5015, 13818* (P), *Jard. Bot. Tananarive 48-1* (P), *M. Keraudren 1131, 25405, 25405bis* (P); Bongolava Region, NW of Ambohitsaratelo-Bebao, *L.J. Dorr 3580* (BR), *L.C. Barnett 354* (P); Ambohibiby Montane, *J. Bosser 19570* (P); Central-West Imerina, *J.M. Hildebrandt 3826* (BR); Fianarantsoa, Haute Matsiatra, Andrambovato, undergrowth of rainforest, *J. Bosser 18279* (P); Melaky, NW of Ambohitsaratelo-Bebao, *L.J. Dorr 3580* (US, WAG); *A. Rakotozafy & R. Rajemisa 354* (WAG); Sofia region, Bealalana, Mangidirano, *L.J. Razafitsalama 350*, *358* (P); Ankaizinana, *R. Decary 1895* (P); Anosy region, Betroka, Kalambatritra Protected Area, *R. Razakamalala 1983* (P).

Eastern Domain: Atsimo-Antsinanana region, Farafangana, Ivongo, *C.|Rakotovao 8522* (P); Vatovavy region, Ranomafana NP, *H. Schaefer et al. 128* (DBEV, TUM), *F. Almeda 8005* (CAS), *D.T. Franklin 9096* (CAS), *G.E. Schatz 1717,1718* (P, WAG), *P.B. Phillipson 2212* (P, US), *G. Schatz 3175, 3177* (MO), *J. Renoult ma07307* (P), *H. Schaefer et al. 105*, *106, 107, 116, 117*, *118* (DBEV, TUM); Haute Matsiatra region, Ivohibe, Northern slopes of Ivohibe peak, *R. Decary 5695* (P); Amoron’i Mania region, Ambatofinandrahana, *H. Perrier de la Bâthie 6767* (P), *M. Keraudren 246* (P), *R. Decary 13286* (P); towards Fenoarivo, *M. Keraudren 191* (P), *H. Perrier de la Bâthie 6768* (P); Southern Ambositra, *S. Elliot 2012* (BR); Haute Matsiatra region, Ialatsara, *R. Decary 17450* (P); Diana region, Montagne d’Ambre NP, *H. Schaefer & M.B. Andriamiharisoa 48* (TUM, DBEV), *O. Andrianantoanina & R. Bezara 141* (P, BR, WAG); Alaotra Mangoro region, Lakato, *A. Randrianasolo 1166* (BR), *R. Decary 18327* (P); Ankeniheny, *Razanatsima 450* (MO, TAN); Sandrangato, *B. Descoings 58* (P); Ankaroka, *G. Cours 217* (P); Rahobevava, *G. Cours 4304* (P); Rahoberava, *Herb. Inst. sc. Madagascar 4304* (P); Alaotra Lake, *Jard. Bot. Tananarive 4048* (P), *M. Keraudren 1166* (P).

**Figures 105–110.**
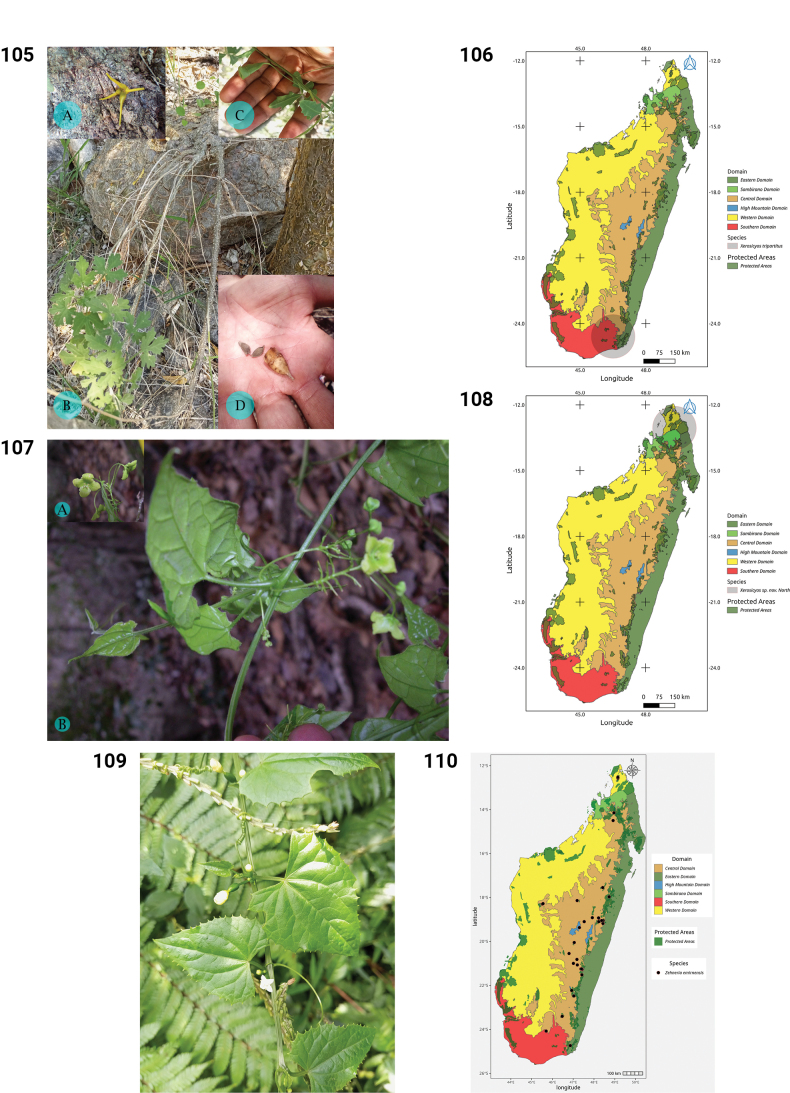
**105.***Xerosicyos tripartitus*. **A.** Male flower; **B.** Habit; **C.** Fruit; **D.** Seeds (photos by MBA, Tranomaro New Protected Area, December 2024). **106.** Distribution of *Xerosicyos tripartitus*. **107.***Xerosicyos* sp. nov. North. **A.** Male inflorescence; **B.** Habit (photos by M. Callmander, Vohémar, October 2004). **108.** Distribution of *Xerosicyos* sp. nov. North. **109.***Zehneria emirnensis*, female plant (photo by MBA, Ranomafana NP, October 2023). **110.** Distribution of *Zehneria emirnensis*.

Southern Domain: Androy Region, Ampandrandava, *A. Seyrig 575* (P); Ampandrandava, *Jard. Bot. Tananarive 5871* (P); Anosy Region, Andohahela Mountain and Elakelaka, *H. Humbert 14122* (P), *H. Schaefer et al. 56, 57* (DBEV, TUM); Amboahangy, *H. Humbert 6843* (P).

**Occurrence inside Protected Areas.** Andohahela NP, Andringitra NP, Montagne d’Ambre NP, Ranomafana NP, Harmonious Protected Landscape of the Ambositra-Vondrozo Forest Corridor, Harmonious Protected Landscape of Lac Alaotra, Natural Resource Reserve of the Ankeniheny-Zahamena Corridor, Tsaratanàna Strict Nature Reserve, and Kalambatritra Special Reserve.

**Ecology and habitat.** Medium-altitude moist evergreen forest, montane ericoid thicket in herbaceous undergrowth, on roadsides, forest edges, and degraded weedy areas on torrent rocks, lateritic soils, and cipolin rockeries, c. 200–2,150 m asl.; flowering Dec.-Feb., fruits Feb.-Mar.

**Taxonomy.** According to Dwivedi et al. (2018), *Z. emirnensis* is sister to *Z. perrieri*.

**IUCN.** Based on IUCN criterion B2, we suggest Least Concern (LC) for a species with wide distribution.

***Zehneria madagascariensis* Keraudren, Adansonia, n.s., 4: 335 (1964)**

Fig. 111

**Holotype.** Madagascar • environs d’Antsirabe, laves d’Iantsifatra, 1650, 25 Dec. 1928, *H. Humbert 7149* (P00135429!).

**Isotypes.** P (P00135430!, P00135431!, P00135432!).

**GenBank information.** Has been sequenced by the authors (unpubl. data).

**Distribution.** Endemic to Madagascar where it has been collected only in the higher regions of the Centre (Fig. 112).

High Mountain Domain: Haute Matsiatra region, Sendrisoa, riverbank, *Réserves Naturelles Madagascar-Razafindrakoto 3082 RN* (P).

Central Domain: Vakinankaratra region, Antsirabe, Iantsifitra lavas, *H. Humbert 7149* (P).

**Occurrence inside Protected Areas.** Andringitra NP.

**Ecology and habitat.** Basaltic rocks, volcanic soils, 1,600–1,700 m asl.; flowering throughout the year (Keraudren 1966), fruits found in June.

**Taxonomy.** Unresolved.

**IUCN.** We suggest classifying as Critically Endangered (CR) based on criterion B1 and B2.

***Zehneria martinez-crovettoi* Keraudren, Adansonia, n.s., 4: 335 (1964)**

Fig. 113

**Holotype.** Madagascar • Toliara, environs d’Ampandrandava (entre Bekily et Tsivory), lit du Torrent de Pisopiso 800 m d’altitude, Nov. 1942, *A. Seyrig 260* P (P00135437!).

**Isotype.** P (P00135438!, P00135439!, P00135440!).

**GenBank information.** Has been sequenced by the authors (unpubl. data).

**Distribution.** Endemic to Madagascar where it has been reported only from the Southern and Western Domain (Fig. 114).

Western Domain: Ihorombe region, Sakamalio Valley, rocks, gneiss, xerophilous bush, *H. Humbert 13312* (P).

Southern Domain: Androy region, Ampandrandava, between Bekily and Tsivory, *A. Seyrig 260*, *260b* (P).

**Occurrence inside Protected Areas.** Not known from protected areas.

**Ecology and habitat.** Dry spiny thicket in riverbed, on sands and rocks, c. 650 m asl., flowering throughout the year (Keraudren 1966), fruits Feb.-Mar.

**Taxonomy.** Unresolved.

**IUCN.** Due to the lack of spatial occurrence data, we suggest Data Deficient (DD).

***Zehneria peneyana* (Naudin) Schweinf. & Asch., Beitr. Fl. Aethiop.: 268 (1867)**

Fig. 115

**Basionym.***Pilogyne peneyana* Naudin, *Ann. Sci. Nat., Bot.* sér. 5, 5: 38 (1866).

**Synonyms.***Melothria peneyana* (Naudin) Cogn., *Monogr. Phan.* 3: 592 (1881).

**Holotype.** Sudan • Nibblanc, 29 Dec. 1840, *Peney s.n.* (P, BR0000008889089!).

**GenBank information.** DNA sequence published by Ranjan et al. (2022): MZ409453.

**Distribution.***Zehneria peneyana* is native to Sudan, South-Eastern Kenya to North-Eastern Tanzania. In Madagascar, it has been recorded in the West (Fig. 116).

Western Boeny region, West Bekalila, Marovoay District, on rice field slope, *J. Bosser 8416* (P); Sofia region, Trabonjy, *J.M. Hildebrandt 3448* (P); Menabe Region, Mahabo farm, marsh edge grass, *J. Dequaire 27166* (P).

**Occurrence inside Protected Areas.** Not known from protected areas.

**Ecology and habitat.** Rice paddies and marsh edges, c. 60–200 m asl.; flowering Sept.–May, fruits in Sept. (Keraudren 1966).

**Taxonomy.** According to Ranjan et al. (2022), *Z. peneyana* is phylogenetically close to *Z. pallidinervia*.

**IUCN.** Due to the lack of spatial occurrence data, we suggest Data Deficien*t* (DD).

***Zehneria perrieri* Keraudren, Adansonia, n.s., 4: 337 (1964)**

Fig. 117

**Holotype.** Madagascar • Analamazaotra, *H.Perrier de la Bâthie 6789* P (P00135441!).

Holotype of Zehneria perrieri var. parvula Keraudren: Madagascar • Ifandana, Farafangana, *R. Decary 5167* P (P00135442!).

Holotype of Zehneria perrieri var. tsaratananensis Keraudren: Madagascar • Mont Tsaratanana, altitude 1,700–2,000 m, 1923, *H. Perrier de la Bâthie 15494* P (P00135445).

**GenBank information.** DNA sequences published by Dwivedi et al. (2018): KY523270, KY523339, KY523378, KY523444.

**Distribution.** Endemic to Madagascar where Z. perrieri var. perrieri is present in the Central, and Western Domain (Fig. 118).

Central Domain: Diana region, Montagne d’Ambre, rainforest, *J. Bosser 20338* (P), *S.M. Trigui 277* (P), *H. Schaefer & M.B. Andriamiharisoa 41*, *41B* (DBEV, TUM); Analamanga region, Angavokely, *J. & M. Peltier 1903* (P); *M. Keraudren 25178*, *25223* (P); 30 km East of Antananarivo, *Service Forestier Madagascar 96 SF* (P); around Antananarivo, *M. Keraudren 30* (P); Ravine cut by the new road, *J. Léandri 3243* (P), *J. Bosser 13624, 13625, 15302* (P); Tsinjoarivo, Onive waterfall, *M. Keraudren 1560* (P); Amoron’i Mania region, Ambatofitorahana, *M. Keraudren 246* (P); Ambatomboay, *C. Rakotovao 9355* (P); Haute Matsiatra region, Mahazony, Ambalavao, *C. Rakotovao 9191bis* (P); Tsitondroina township, *Herb. Jard. Bot. Tananarive 4802* (P); Vatovavy region, Ranomafana NP, *H. Schaefer et al. 113, 120, 122, 124* (DBEV, TUM); Ihorombe region, Begogo, *N.M. Andrianjafy 766* (P); Ivohibe, *G. Cremers 1515* (P); Alaotra-Mangoro region, Forest northern Sihanaka, *Herb. Jard. Bot. Tananarive 2936* (P); Mangoro, *H. Perrier de la Bâthie 18072* (P); Analamazaotra, *H. Perrier de la Bâthie 6789* (P), *M. Keraudren 1746* (P); Anoribe, *J. Bosser 16462* (P); Ambatosoratra, *C. Rakotovao 11028* (P).

**Figures 111–116.**
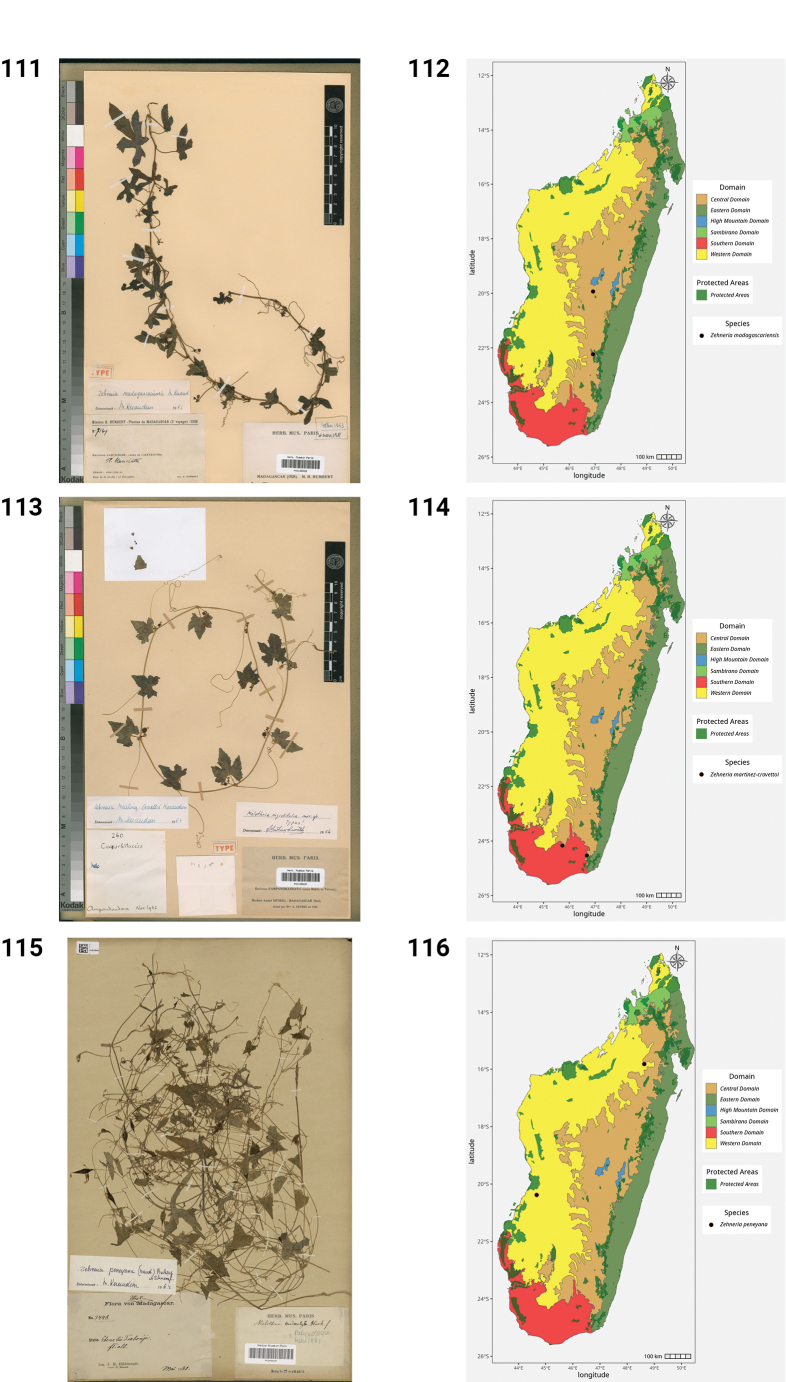
**111.***Zehneria madagascariensis*, Holotype, Antsirabe, *H. Humbert 7149* (P). **112.** Distribution of *Zehneria madagascariensis*. **113.***Zehneria martinez-crovettoi*, Holotype, Ampandrandava, *A. Seyrig 260* (P). **114.** Distribution of *Zehneria martinez-crovettoi*. **115.***Zehneria peneyana*, Trabonjy, *J.M. Hildebrandt 3448* (P). **116.** Distribution of *Zehneria peneyana*.

Western Domain: Anosy region, Andohahela Massif (South-East), rainforest on the gneissic ridge, *H. Humbert 13620* (P); humid forest, Andohahela NP, *H. Schaefer et al. 53, 55* (DBEV, TUM).

Zehneria perrieri var. parvula is only known from the type collected at Farafangana: Eastern Domain, Atsimo-Antsinana region, Farafangana, Ifandana, forest edge, *R. Decary 5167* (P).

Zehneria perrieri var. tsaratananensis was described from the High Mountain Domain, Diana region, in herbaceous undergrowth of Mount Tsaratanana, c. 2,000 m asl., *H. Perrier de la Bâthie 15494, 16421* (P).

**Occurrence inside Protected Areas.**Zehneria perrieri var. perrieri: Montagne d’Ambre NP, Ranomafana NP, Andohahela NP. – Zehneria perrieri var. parvula: Not known from protected areas. – Zehneria perrieri var. tsaratananensis: Tsaratanana Integral Natural Reserve.

**Ecology and habitat.**Zehneria perrieri var. perrieri grows in undergrowth of medium altitude moist evergreen forest, near waterfalls, in forest remnants, and primary vegetation, preferably on gneiss, c. 1,000–1,800 m asl. Flowering throughout the year (Keraudren 1966), fruits found in May.

Zehneria perrieri var. parvula was found at a forest edge; fruits in Sept. (Keraudren 1966).

Zehneria perrieri var. tsaratananensis was found in the undergrowth of forest bordering montane grassland, c. 1,700–2,000 m asl.; fruits found in April (Keraudren 1966)

**Taxonomy.** According to Dwivedi et al. (2018)*Z. perrieri* is sister to *Z. emirnensis*.

**IUCN.**Zehneria perrieri var. perrieri: Based on IUCN criterion B2, we suggest Least Concern (LC) for a species with wide distribution.

*Zehneria perrieri var. parvula* and Z. perrieri var. tsaratananensis: We suggest Data Deficient (DD), as there are insufficient occurrence records to enable a proper assessment of the taxa, which have not been seen for decades and might have become extinct already.

***Zehneria polycarpa* (Cogn.) Keraudren, Adansonia, n.s., 4: 333 (1964)**

Fig. 119

**Basionym.***Melothria polycarpa* Cogn., *J. Linn. Soc., Bot.* 29: 20 (1891).

**Holotype.** Madagascar • Fort-Dauphin, *G.F. Scott Elliot 2316* K (K000313362!).

**Isotype.** P (P00135446!).

**GenBank information.** DNA sequences published by Dwivedi et al. (2018): KY523276, KY523342, KY523381, KY523439.

**Distribution.** Endemic to Madagascar where it is found mainly in the North and East (Fig. 120).

Western Domain: Montagne des Francais, *M. Keraudren 1640* (P); Ankarana, *M. Keraudren 1692* (P), *H. Schaefer & M.B. Andriamiharisoa 23, 23B* (DBEV, TUM); Manongarivo Massif, *H. Perrier de la Bâthie 6785* (P); Ihorombe region, Zazafotsy, *G. Cremers 581* (P).

Sambirano Domain: Ambanja, *Réserves Naturelles Madagascar 7435 RN* (P), Nosy Komba, *L.H. Boivin 2132 bis* (P); Nosy-Be, *L.H. Boivin s.n.* (P).

Central Domain: Diana region, Montagne d’Ambre NP, *O. Andrianantoanina 986* (P), *J. Bosser 20320* (P); Analamaitso forest, Anivorano North, *M. Keraudren 1713* (P); Sava region, Daraina, Antsahabe forest, *A. Rakotondrafara 344* (P); Anhabe forest, *L. Nusbaumer 1276* (P); Alaotra Mangoro region, Andasibe NP, *H. Schaefer et al. 3, 4, 17, 18, 19* (TUM, DBEV); Mantadia NP, *H. Schaefer et al. 8, 10* (TUM, DBEV).

Eastern Domain: Vatovavy region, Ranomafana NP, *Schatz G.E. 1718* (BR), *H. Schaefer et al. 103, 104, 114, 115* (DBEV, TUM); Anosy region, Andohahela NP, *H. Schaefer et al. 49, 51, 52*; Amboahangy, Fort-Dauphin, *G.F. Scott Elliot 2542* (BR, K), *M. Keraudren 1030, 1490* (P), *P. Morat 1336* (P), *M. Decorse s.n.* (P); St Louis Peak, *H. Humbert 5909* (P), *J. Bosser 13559* (P), *G.F. Scott-Elliot 2316* (BR, K, P), *N. Dumetz 559* (WAG); Mandrare Valley, *C d’ Alleizette, s.n.* (L).

Southern Domain: Androy region, Upper basin of Mandrare, *H. Humbert 6843*, *6590* (P); Manambolo Valley, *H. Humbert 13291* (P).

**Occurrence inside Protected Areas.** Andohahela NP, Analamazaotra NP, Mantadia NP, Montagne d’Ambre NP, Ranomafana NP, Ambohitr’Antsingy-Montagne des Français Harmonious Protected Landscape, Loky Manambato Harmonious Protected Landscape, and Ankarana Special Reserve.

**Ecology and habitat.** Medium altitude moist evergreen to moist semi-deciduous forest, on roadside and riverbanks, in cliff, and dunes, growing in lateritic gneiss, limestone soils, and sands, c. 10–1,200 m asl.; flowering Oct.-Mar., fruits Oct.-July.

**Taxonomy.**Ranjan et al. (2022) place *Z. polycarpa* close to *Z. parvifolia*.

**IUCN.** Based on IUCN criterion B2 we suggest Least Concern (LC) for a species with wide distribution.

***Zehneria rutenbergiana* (Cogn.) Keraudren, Adansonia, n.s., 4: 333 (1964)**

Fig. 121

**Basionym.***Melothria rutenbergiana* Cogn., *Abh. Naturwiss. Vereins Bremen* 7: 251 (1882).

**Vernacular name.** Siramboalavo (Sihanaka).

**Holotype.** Madagascar • Ambatondrazaka, Lac Alaotra, *Rutenberg s.n.* (B; destroyed).

**Neotype.** Madagascar • Central, s.d., *R. Baron 2348* K (K000391257!), designated by Keraudren (1966), *Cucurbitacees, Fl. Madagasc.* 185: 47.

**GenBank information.** DNA sequence published by Ranjan et al. (2022): MZ409454.

**Distribution.** Endemic to Madagascar, where it has been found only in the Alaotra region (Fig. 122).

Central Domain: Alaotra-Mangoro region, Ambatondrazaka, Ambatosoratra, Andreba Gara, Alaotra Lake, *Rakotoarivelo 842* (MO); Ambatondrazaka, Andaingo Gara, Bembary, *Randriambololomamonjy 466* (MO, TAN); Ambohinajanhary, *J. Bosser 8179* (P).

**Figures 117–122.**
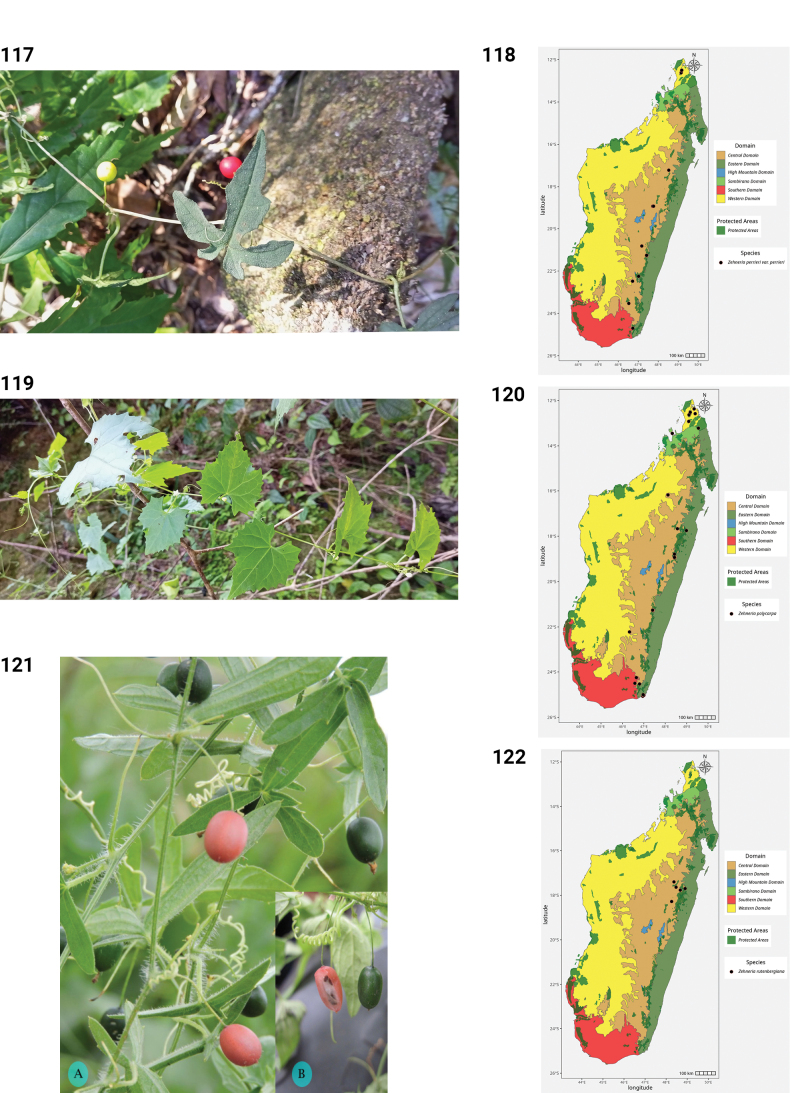
**117.**Zehneria perrieri var. perrieri, plant with green (immature) and red (mature) fruits (photo by MBA, Ranomafana NP, October 2022). **118.** Distribution of Zehneria perrieri var. perrieri. **119.***Zehneria polycarpa*, habit (photo by MBA, Andohahela NP, September 2022). **120.** Distribution of *Zehneria polycarpa*. **121.***Zehneria rutenbergiana*. **A.** Habit; **B.** Longitudinal section of ripe fruit (photos by N. Manjato, Andreba Gara, January 2017). **122.** Distribution of *Zehneria rutenbergiana*.

Eastern Domain: Zahamena, Manaka-East Township, Ambatondrazaka District, *C. Rakotovao 11277*, *11821* (P).

**Occurrence inside Protected Areas.** Lac Alaotra Harmonious Protected Landscape and Zahamena NP.

**Ecology and habitat.** Marshes and bog and island habitats associated with *Cyperus latifolius* and *Psidium guajava*, c. 800 m asl.; fruits in June.

**Taxonomy.** According to Dwivedi et al. (2018), *Z. rutenbergiana* is sister to *Z. capillacea*.

**IUCN.** We suggest Endangered (EN) based on IUCN criterion B2.

***Zehneria tridactyla* (Hook.f.) R.Fern. & A.Fern., Mem. Junta Invest. Ultramar, 2 Ser. 34: 118 (1962)**

Fig. 123

**Basionym.***Melothria tridactyla* Hook.f., *Fl. Trop. Afr.* 2: 562 (1871).

**Lectotype.** Mozambique • Shupanga, climbing on trees and among grass; Apr. 1862, *J. Kirk, s.n*. K (K000313374!), designated by Bräuchler et al. (2016), Phytotaxa 284(2): 144.

**Syntypes.** Angola • *F.M.J. Welwitsch 826* (BM, G00458275!, K); Congo • *R. Burton s.n.* K (K000313436!); Mozambique • *J. Kirk s.n.* K (K000313373!).

**GenBank information.** DNA sequences published by Dwivedi et al. (2018): KY523415, KY523414, KY523318, KY523466, KY523467, KY523350, KY523317.

**Distribution.** Native to Southwest India, Sri Lanka, and Madagascar, where it is scattered throughout the country (Fig. 124).

Western Domain: Diana region, Lohariandava, Andovoranto, *R. Viguier 621* (P); Ankarana, *H. Humbert 25531* (P), *M. Keraudren 1690* (P); Boeny region, Marovoay, *K. Afzelius s.n.* (P); Ambongo-Boïna, *H. Perrier de la Bâthie 6772* (P); Melaky, Tsingy de Bemaraha, *C.C.H. Jongkind* (WAG); Bongolava region, Babay-ville Sakay, *M.W. Armand 129* (P); Kianjasoa, *J. Bosser 783* (P), *Jard. Bot. Tananarive 171* (P); Kinkony, *R. Decary 7752* (P).

Sambirano Domain: Diana region, Nosy-Be, *A. Pervillé s.n.* (P), *L.H. Boivin 2132* (P).

Central Domain: SAVA region, Marojejy massif, *H. Humbert 22142bis* (P); Betsiboka region, Maevatanana, *H. Perrier de la Bâthie 1074* (P). Sofia region, Ankaramy, Maromandia (Ankaramy), *R. Decary 1318* (P); Alaotra-Mangoro region, Ivondro river, *J.P. Goudot 1833* (BR); Atsimo-Andrefana region, Sakaraha, Zombitsy, *T. Mitchell & H. Schaefer* (TUM).

Eastern Domain: Fenoarivo-Antsiranana, Sainte Marie, *L.H. Boivin s.n.* (P). Alaotra-Mangoro, Ambodimanga, *G. Cours 476* (P); Ambatosoa region, Mananara, *R. Decary 55* (P).

**Occurrence inside Protected Areas.** Tsingy de Bemaraha NP, Mahavavy Kinkony Wetlands Complex Harmonious Protected Landscape, and Zombitse-Vohibasia NP.

**Ecology and habitat.** Marshes and bogs, rice fields, forest edges, and in montane ravines, c. 250–800 m asl.; flowering Sept.–May, fruits June–Aug.

**Taxonomy.***Zehneria tridactyla* is sister to *Z. giletii*, and *Z. capillacea* (Dwivedi et al. 2018).

**IUCN.** We suggest Least Concern (LC) based on IUCN criterion B2.

**Figures 123, 124.**
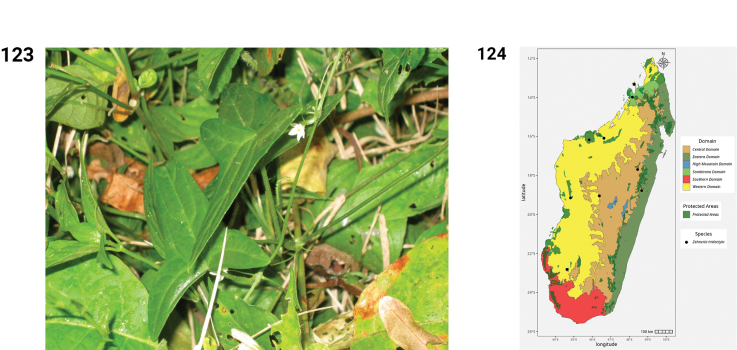
**123.***Zehneria tridactyla*, habit (photo by HS, Zombitsy-Vohibasia NP, March 2013). **124.** Distribution of *Zehneria tridactyla*.

### ﻿﻿Naturalised species

Six species of Cucurbitaceae are classified as naturalised species (Table 2) which have been introduced on purpose or by accident in the recent past and established permanent populations in natural habitats.

***Coccinia* Wight & Arn., Prodr. Fl. Ind. Orient. 1: 347 (1834).**

**Generic type.***Coccinia indica* Wight & Arn.

**Worldwide Distribution.** Africa and 1 species introduced in tropical regions worldwide (Schaefer 2020).

***Coccinia grandis* (L.) Voigt, Hort. Suburb. Calcutt. 59 (1845)**

Fig. 125

**Basionym.***Bryonia grandis* L., *Mant. Pl.* 126 (1767).

**Lectotype.** India • *Linnaeus s.n.* (LINN No. 1153.2), designated by Jeffrey (1967), Fl. Trop. E. Afr., Cucurbitac.: 68.

**GenBank information.** Sequence data available on GenBank from Holstein and Renner (2011); Malagasy material has been sequenced by the authors (unpubl. data).

**Distribution.** Native to Tropical Africa, the Western Arabian Peninsula, Tropical and perhaps subtropical Asia. Introduced in Madagascar, where it is found cultivated and escaped in the South (Fig. 126).

Southern Domain: Atsimo-Andrefana region, Andranomaitso, near Zombitsy NP, *H. Schaefer et al. 83* (DBEV, TUM).

Easter Domain: Anosy region, Fort Dauphin, city, *H. Schaefer et al. 59* (DBEV, TUM); Cultivated, climbing fence, Nahampoana Village, *H. Schaefer et al. 27* (January 2025).

**Occurrence inside Protected Areas.** Not known from protected areas.

**Ecology and habitat.** Disturbed ground near settlements, on sandy soil, c. 50 m asl.; flowers and fruits Oct.-Dec.

**Uses.** Young leaves, shoots, and green fruits are used as vegetables in Asia (HS, pers. obs.). No such use has been documented from Madagascar.

**Taxonomy.** According to Holstein and Renner (2011), *C. grandis* is sister species to *C. ogadensis*.

***Cucumis* L., Sp. Pl. 1011. 1753.**

Fig. 127

*Cucumis anguria* L., Sp. Pl. 2:1011 (1753)

**Type.** Jamaica (cult. in Sweden) • *Linnaeus s.n.* (Lectotype. LINN 1152.6), designated by Staples, in RA Howard, Fl. Less. Antill., Dicot. 3: 482 (1989).

**GenBank information.** DNA sequences published by Endl et al. (2018): KY434397, KY458066, KY434332, HM597065, KY434505.

**Distribution.** Native to the African continent (Angola, Botswana, KwaZulu-Natal, Malawi, Mozambique, Namibia, Northern Provinces, Swaziland, Tanzania, Zambia, Zaire, Zimbabwe), introduced and invasive in many (sub)tropical regions of the world (POWO 2023).

In Madagascar, *C. anguria* occurs only near settlements in the extreme South (Fig. 128), where it has been misidentified as *C. africanus* in the past (e.g., *Keraudren 1001* (P)), a species which probably does not (or no longer) exist in the country.

Southern Domain: Androy region, Ambovombe, *R. Decary 9540* (P); Anarafaly, *J. Bosser 13568, M. Keraudren 1001* (P); Androka, *J. Bosser 301* (P); Tranomaro New Protected Area, *H. Schaefer et al. 7* (DBEV, TUM); Atsimo-Andrefana region, Ranobe Forest, Ankilimalinike, *P.B. Phillipson 5927* (P); Anosy region, dry forest, Andohahela NP, *H. Schaefer et al. 66, 69* (DBEV, TUM).

**Occurrence inside Protected Areas.** Andohahela NP, Ankodida Harmonious Protected Landscape.

**Ecology and habitat.** Disturbed ground in dry spiny thicket and dry deciduous clearings, in savanna with scattered bushes on sandy soils, c. 50–100 m asl.

**Uses.** In South America and the West Indies, the fruits are grown as vegetables (Baird and Thieret 1988) but no such use has been documented in Madagascar.

**Taxonomy.***Cucumis anguria* is sister to the clade including *C. pustulatus, C. prophetarum, C. dipsaceus, C. ficifolius, C. baladensis*, and *C. rigidus* (Sebastian et al. 2010).

***Cucumis dipsaceus* Ehrenb. ex Spach, Hist. Nat. Vég. 6: 211 (1838)**

Fig. 129

**Holotype.** Saudi Arabia • *G. Ehrenberg s.n.* (B, destroyed).

**Figures 125–128.**
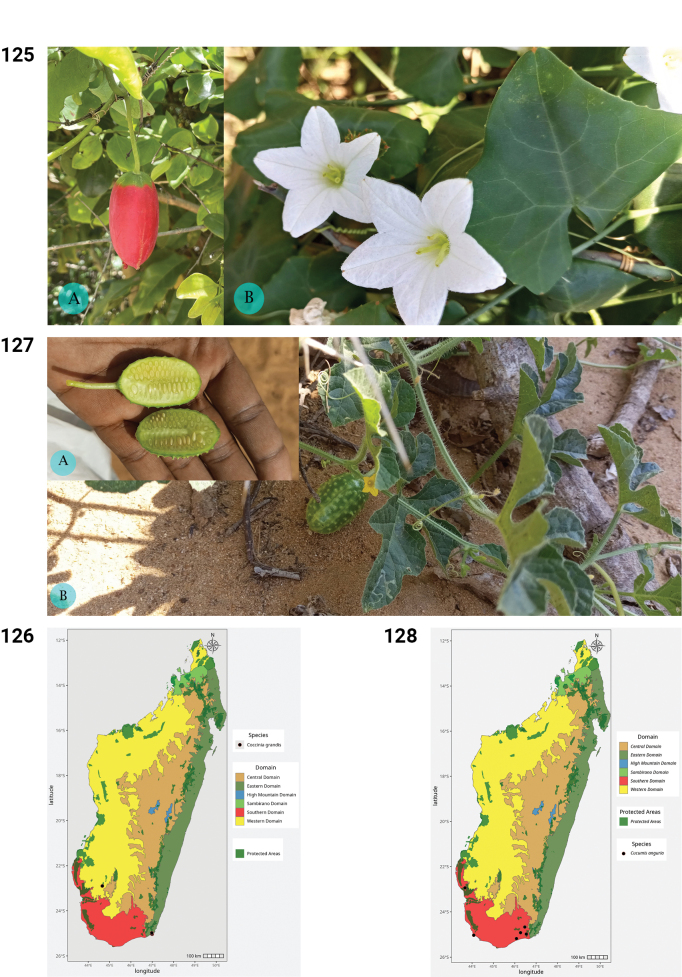
**125.***Coccinia grandis*. **A.** Mature fruit; **B.** Flower (photos by S. Philizot, Toliara, February 2024 (**A**) and by MBA, Fort Dauphin, October 2022 (**B**)). **126.** Distribution of *Coccinia grandis*. **127.***Cucumis anguria*. **A.** Habit; **B.** Longitudinal section of the fruit (photos by MBA, Andohahela NP, October 2022). **128.** Distribution of *Cucumis anguria*.

**Neotype.** Saudi Arabia • Wadi Kamme east of al-Qunfidha, Feb. 1825, *G. Ehrenberg & Hemprich s.n.* (MPU), designated by Kirkbride, Biosyst. Monogr. Cucumis 45 (1993).

**Synonyms.***Momordica dasycarpa* Hochst. ex A.Rich., *Tent. Fl. Abyss.* 1: 291 (1848).

**GenBank information.** DNA sequences for this taxon (but not from Malagasy material) published by Ghebretinsae et al. (2007), Renner et al. (2007), Endl et al. (2018): AF006800, AF006801, AF013310, DQ785828, DQ785844, EF595872, EF595922, EF093513, MZ489698, MG993578, MG993587.

**Distribution.** Introduced to Madagascar and invasive in the South (Fig. 130).

Southern Domain: Tranomaro Village, *H. Schaefer et al. 4, 9A, 9B* (DBEV, TUM); Anosy region, Bevilany village, roadside, Andohahela NP, *H. Schaefer et al. 67* (DBEV, TUM).

iNaturalist observations: https://www.inaturalist.org/observations/25662008; https://www.inaturalist.org/observations/70549193.

**Occurrence inside Protected Areas.** Andohahela NP, Amoron’i Onilahy Harmonious Protected Landscape.

**Ecology and habitat.** Disturbed areas of dry spiny thicket, often climbing on roadsides and in xerophytic shrub, c. 100 m asl.; flowers and fruits Oct.–Feb.

**Uses.** In Peru, the fresh fruits of *C. dipsaceus* are used for dandruff, adding shine and beauty to hair, hair loss prevention, and to stop babies from breastfeeding (Bussmann and Sharon 2016). No such use has been documented in Madagascar.

**Taxonomy.***Cucumis dispsaceus* is sister species to *C. ficifolius* (Sebastian et al. 2010).

***Cucumis melo* L., Sp. Pl.: 1011 (1753)**

Fig. 131

**Holotype.** cult. at Uppsala (Sweden), *Linnaeus s.n.* (Herb. LINN No. 1152/8), designated by Meeuse, Bothalia 8 (1962): 61.

**Synonyms.**https://powo.science.kew.org/taxon/292238-1.

**Vernacular name**. Voatango (Merina).

**GenBank information.** DNA sequences of the species published e.g., by Endl. et al. (2018): KY434411, KY434412, KY458087, KY458086, KY434470, KY434472, KY434471, KY434354, KY434355.

**Distribution.** Cultivated in Madagascar for its fruit and occasionally found in the wild (Fig. 132).

Western Domain: Diana region, Manongarivo Special Reserve, *L. Gautier 2972* (MO); Ankarana, *H. Schaefer & M.B. Andriamiharisoa 28, 31*, *31B* (TUM, DBEV); Menabe, West coast, from Manombo to Morondava, *A. Grandidier s.n.* (P); Mahobo Farm, *J. Dequaire 27010*; Andranofotsy transit hut, *H. Poisson 482* (P).

Sambirano Domain: Diana region, Nosy Be, *L.H. Boivin 2129* (P).

Central Domain: Analamanga region, *C. d’Alleizette* (P); Alaotra-Mangoro region, East-Ilaka, *J. Bosser 17024* (P).

Eastern Domain: Alaotra-Mangoro region, Andasibe-Perinet village, *H. Schaefer et al. 12* (TUM, DBEV).

Southern Domain: Atsimo-Andrefana region, north of Toliara, *A. Rakotozafy* (P); Ambolonkira, *M. Keraudren 664* (P), *J. Bosser 13581* (P); Vohitany, *M. Keraudren 1436* (P); Near Ambatry southern Betioky, *M. Keraudren 790* (P); Andatabo, *M. Keraudren 556* (P); Mahafaly limestone plateau, *M. Keraudren 867* (P); Ambiamena, Zombitsy NP, *H. Schaefer et al. 91* (DBEV, TUM), *B. Descoings 2275* (P); near Andabolava, *H. Humbert 12419*, *27193* (P); Tanandava, Mangoky, *J. Bosser 16092* (P); Ampandrandava, *A. Seyrig 557* (P).

**Occurrence inside Protected Areas.** Manongarivo Special Reserve, Analamazaotra NP, Ranobe PK32 New Protected Area, and Zombitse-Vohibasia NP.

**Ecology and habitat.** Cultivated for its fruit and often found as an escaped plant on disturbed ground from sea level up to 1,800 m asl. The wild *Cucumis melo* with tiny, bitter fruits differs genetically from the cultivated species (Endl et al. 2018) and may have specific ecological preferences that require further field study.

**Uses.** Fruit eaten raw, seeds roasted.

**Taxonomy.***Cucumis melo* forms a clade that is sister to *C. callosus*, and *C. trigonus* (Endl et al. 2018).

***Diplocyclos* (Endl.) Post & Kuntze, Lex. Gen. Phan.: 178 (1903).**

**Generic type.***Bryonia affinis* Endl.

**Worldwide Distribution.** Old world Tropics and Subtropics (Schaefer 2020).

***Diplocyclos palmatus* (L.) C. Jeffrey, Kew Bull. 15: 352 (1962)**

Fig. 133

**Basionym.***Bryonia palmata* L., *Sp. Pl.*: 1012 (1753).

**Holotype.** Sri Lanka • *P. Hermann s.n* BM (BM000621700!).

**GenBank information.** DNA sequences of this taxon (but not from Malagasy material!) published by Kocyan et al. (2007) and Renner and Schaefer (2016): AY862552, DQ536671, DQ536769, KT779308.

**Distribution.** Only found in the North in Montagne d’Ambre and Ankarana (Fig. 134) where it was first collected by Bosser in June 1970 (Keraudren-Aymonin 1982b) and is still very localised.

Central Domain: Diana region, Montagne d’Ambre, *J. Bosser 20358* (P), *H. Schaefer & M.B. Andriamiharisoa 45, 45B, 45C* (TUM, DBEV), *Ramandimbimanana 134* (MO, P), *S.M. Trigui et al. 506* (P, WAG), *S.D. Ramandimbimanana 134* (P, WAG), *G.E. Schatz 1514* (MO, P).

Western Domain: Matsaborimanga, Ankarana Special Reserve, *M. Bardot-Vaucoulon 44* (P).

**Occurrence inside Protected Areas.** Montagne d’Ambre NP and Ankarana Special Reserve.

**Ecology and habitat.** Medium-altitude moist evergreen forest and thicket vegetation on limestone (tsingy) or volcanic soils, from 300–1,000 m asl.

**Uses.** The fruits of *D. palmatus* are used to treat female infertility on the African continent (Gautam et al. 2013) but no such use has been documented in Madagascar.

**Taxonomy.***Diplocyclos palmatus* is sister species to *D. schliebenii* (Holstein and Renner 2011).

***Momordica* L., Sp. Pl. 1009. 1753.**

**Generic type.***Momordica charantia* L.

**Worldwide Distribution.** Old world Tropics and Subtropics, one species invasive in many parts of the world (Schaefer 2020).

**Figures 129–134.**
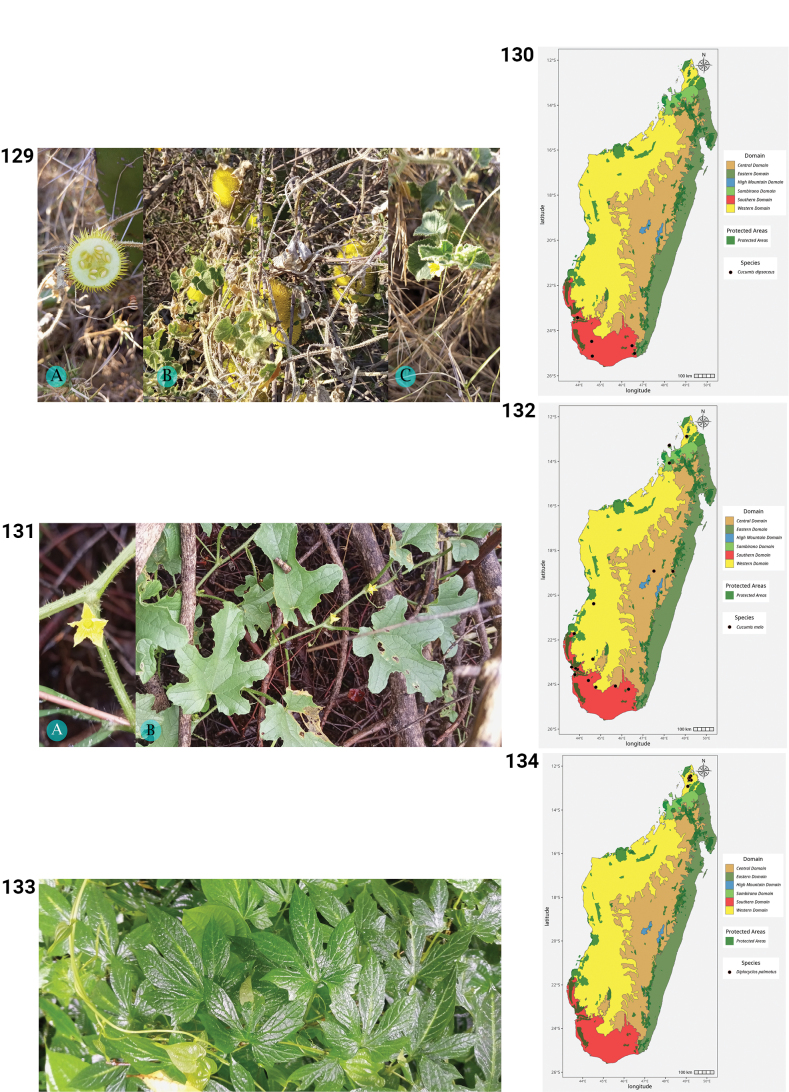
**129.***Cucumis dipsaceus*. **A.** Fruit (cross-section); **B.** Habit; **C.** Flower (photos by MBA, Andohahela NP, October 2022). **130.** Distribution of *Cucumis dipsaceus*. **131.***Cucumis melo*. A. Male flower; B. Habit (photos by MBA, surroundings of Ankarana Special Reserve, December 2023). **132.** Distribution of *Cucumis melo*. **133.***Diplocyclos palmatus*, habit (photo by MBA, Montagne d’Ambre NP, December 2023). **134.** Distribution of *Diplocyclos palmatus*.

***Momordica charantia* L. Sp. Pl. 1009: [s.p.] (1753)**

Fig. 135

**Lectotype.** India • Herb. Clifford: 451, Momordica 2 (BM-000647445), designated by Jeffrey, Fl. Trop. E. Africa, Cucurbitaceae 31 (1967).

**Vernacular name.** Margôzy (Merina).

**Synonyms.***Momordica muricata* Willd., *Sp. Pl.*, ed. 4. 4: 602 (1805); *Momordica papillosa* Peckolt ex Rosenthal, *Syn. Pl. Diaph.*: 678 (1862); *Momordica senegalensis* Lam., *Encycl.* 4: 239 (1797).

**GenBank information.** DNA sequences of the species (but not from Malagasy material!) published e.g., by Kocyan et al. (2007), De Boer et al. (2012), and Urasaki et al. (2016): NW019104488, DQ501269, DQ535760, DQ491019, DQ491013, HE661309.

**Distribution.** Large-fruited forms are only found in cultivation and sometimes as casuals near settlements, but small-fruited forms occur in the wild mainly in the Northern part of the island and require further taxonomic study (Fig. 136).

Central Domain: Diana region, Montagne d’Ambre Montane, *J. Bosser 5921, 20321* (P), *M.H. Razanajatovo 162* (MO, P), *H. Schaefer & M.B. Andriamiharisoa 44B,C* (TUM, DBEV), *D. Meyers 133* (MO, P); Analamanga region, Antananarivo, *M. Keraudren 1559* (P); Ihorombe region, Isalo NPs, *A.M. Homolle 1456* (P).

iNaturalist observations: https://www.inaturalist.org/observations/51929066, https://www.inaturalist.org/observations/89910287, https://www.inaturalist.org/observations/14066793, https://www.inaturalist.org/observations/25434726), https://www.inaturalist.org/observations/77727335.

Western Domain: Diana region, Diego-Suarez, *L.H. Boivin 2571* (P), *M. Keraudren 1729* (P), *A.C.J. Bernier 146* (P); in Sakaramy, *H. Perrier de la Bâthie 106* (P), *J.M. Hildebrandt 3344* (P); Ankarana Special Reserve, *M. Bardot-Vaucoulon 722, 1256, 1815* (MO, P, TAN), *H. Humbert 25528, 32637, 32638* (BR, P), *H. Schaefer & M.B. Andriamiharisoa 24* (TUM, DBEV); Menabe, Mahabo, Mahabo Farm, *J. Dequaire 27082, 27241* (P); Boeny region, nearest Mahajanga, *H. Perrier de la Bâthie 14675* (P); Ambongo-Boina, *H. Perrier de la Bâthie s.n.* (P); Ampijoroa, Ankarafantsika forest, *M. Keraudren 1238* (BR, P); Tsingy of Namoroka, *Service Forestier Madagascar 18* (P); Sofia region, Ankaizinana, *R. Decary 2069* (P).

iNaturalist observation: https://www.inaturalist.org/observations/66238690.

High Mountain Domain: Manongarivo, *P. Derleth 76* (P).

**Figures 135, 136.**
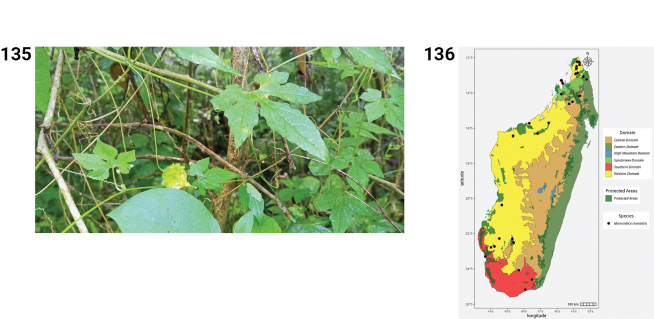
**135.***Momordica charantia*, small-fruited plant from the North (photo by MBA, Montagne d’Ambre, December 2023). **136.** Distribution of *Momordica charantia*.

Sambirano Domain: Diana region, Nosy-Komba, *J.M. Hildebrandt 3229, L.H. Boivin 2131 2* (P); Nosy-be, *Esperandieu 15* (P), *Waterlot 287* (P), *J.M. Hildebrandt 2959* (P), *L.H. Boivin 2131* (P).

Eastern Domain: SAVA region, Daraina, Soloniampilana-Maroadabo, *L. Gautier 4496* (MO), *H. Humbert 32379* (P); Sambava, Bevontro, Tsaratanana, Morafeno, *R. Razakamalala 3096* (MO, P); between Vohémar and Ambilobe, *R. Decary 14644* (P).

Southern Domain: Atsimo-Andrefana region, Ankazoabo, *J. Bosser 17272* (BR, P, MO); Analafanja, *H. Humbert 14301* (P); Mahaboboka, Marotsiraka-Betsileo, *Rakotoarivelo 1019* (MO); Belemboka, *J. Dequaire 27419* (P); Manombo Valley (SW), *R. Decary 18789* (P); Beroroha, Betorabato, *Razakamalala 6104* (MO, TAN); Androy Region, Ampandrandava, *A. Seyrig 811* (P); Ambovombe, *J. Bosser 13570* (P), *M. Keraudren 983* (P); Tranomaro village, *H. Schaefer et al. 11A,B* (DBEV, TUM).

**Occurrence inside in Protected Areas.** Isalo NP, Montagne d’Ambre NP, Marojejy–Anjanaharibe Sud–Tsaratanàna Northern Section Natural Resource Reserve, Ranomafana NP, Ankarafantsika NP, Namoroka NP, Ambohitr’Antsingy Montagne des Français Harmonious Protected Landscape, Loky Manambato Harmonious Protected Landscape, Bongolava Forest Corridor Harmonious Protected Landscape, and Ankarana Special Reserve.

**Ecology and habitat.** Cultivated as a vegetable and medicinal plant. Truly wild plants found on forest edges or in degraded riparian forests at higher altitudes, up to 1,200 m asl.; flowering throughout the year, pollinated by introduced honeybees (*Apis mellifera*).

**Uses**: The fruit is used against dysentery in Madagascar rural communities and is also consumed as vegetable.

**Taxonomy.***Momordica charantia* is sister to *M. angolensis* (Schaefer and Renner 2010).

### ﻿﻿Cultivated species which occasionally escape

Eleven species are cultivated in Madagascar, mostly as vegetables, and are occasionally found on disturbed ground near settlements (Table 3). None of them seems genuinely naturalised with permanent populations outside cultivation. In addition, Keraudren (1966) listed *Benincasa hispida* (Thunb.) Cogn. and *Cucurbita maxima* Duch. but their presence on the island is doubtful.

**Table 3.** Cultivated Cucurbitaceae of Madagascar which occasionally escape.

No.	Scientific name	Status	Distribution
1.	*Citrullus lanatus* (Thunb.) Matsum. & Nakai	Cultivated	Western, Central, Eastern, Sambirano, Southern
2.	*Cucumis sativus* L.	Cultivated	Western, Central, Eastern, Sambirano, Southern
3.	*Cucurbita moschata* Duchesne	Cultivated	Western, Central, Southern
4.	*Cucurbita pepo* L.	Cultivated	Western, Central, Eastern, Sambirano, Southern
5.	*Lagenaria siceraria* (Molina) Standl.	Cultivated	Western, Central, Eastern, Southern
6.	*Lagenaria sphaerica* (Sond.) Naudin	Cultivated	Western, Southern
7.	*Luffa acutangula* (L.) Roxb.	Cultivated	Western, Central, Eastern, Southern
8.	*Luffa aegyptiaca* Mill.	Cultivated	Western, Central, Eastern, Southern
9.	*Sicyos edulis* Jacq.	Cultivated	Western, Central, Eastern
10.	*Telfairia pedata* (Sm.) Hook.	Cultivated	Sambirano
11.	*Trichosanthes cucumerina* L.	Cultivated	Western, Central

***Citrullus* Schrad. ex Eckl. & Zeyh., Enum. Pl. Afric. Austral.: 279 (1836), nom. cons.**

*Citrullus lanatus* (Thunb.) Matsum. & Nakai, Index Seminum (TI, Tokyo) 1915-1916: 30 (1916).

**Basionym.***Momordica lanata* Thunb., *Prodr. Pl. Cap.*: 13 (1794), *nom. cons*.

**Neotype.** USA • St. Louis, grown in 2014 in S. Renner’s Garden in St Louis from the seed of watermelon ‘Crimson Sweet’ bought in a store in St. Louis; 4^th^ July 2014; *S.S. Renner 2816* (M- 0242260!).

**Synonyms.**https://powo.science.kew.org/taxon/291938-1.

**Vernacular names.** Hetsihetsy (Antakarana), Makatendry (Betsimisaraka), Tsikiry (Antakarana, Betsimisaraka), Voabe (Merina, Sakalava), Voaketrihetry, Voaketsihetsy, Voamanga (Betsileo), Voanketsihetsy (Merina, Sakalava), Voantsiriky (Sakalava), Voasavy (Sakalava, Antakarana).

**GenBank information.** DNA sequences published e.g., by Renner et al. (2021): CM031034, JADPLL010000000.

**Pictures.**https://www.inaturalist.org/observations/224425271.

**Distribution.** Native to Africa, probably Sudan, Ethiopia, and Libya (Renner et al. 2021). The species is cultivated and often escaping in all areas of tropical and equatorial regions of the world (Keraudren 1966; POWO 2023). In Madagascar, *Citrullus lanatus* has been reported in all parts of the country (Keraudren 1966) but it seems that most of those records refer to *Citrullus mucosospermus*.

**Ecology and habitat.** Widely cultivated but apparently never truly naturalised.

**Uses.** The watermelon fruit is typically eaten fresh.

**Taxonomy.***Citrullus lanatus* is sister to *C. mucosospermus* (Zhou et al. 2023).

***Cucumis* L., Sp. Pl. 1011. 1753.**

Fig. 137

*Cucumis sativus* L., Sp. Pl.: 1012 (1753)

**Lectotype.***Burser vol. 17, no. 97* (UPS), designated by Ten Pas et al. (1985), Taxon 34: 291.

**Synonyms.**https://powo.science.kew.org/taxon/292296-1.

**Vernacular name.** Voatangombazaha (Merina), Voatsaky (Masikoro, Sakaraha).

**GenBank information.** DNA sequences of the species (but not from Malagasy material!) published e.g., by Plader et al. (2007) and Li et al. (2019): NC007144, NC026655.

**Distribution**: Introduced in Madagascar, probably by Austronesians, but the period remains undetermined (Beaujard 2017). It is often cultivated and occasionally reported as a casual.

Herbarium specimens in P identified by M. Keraudren as *C. sativus* all belong to *C. sacleuxii* (H.S., pers. obs.).

**Ecology and habitat.** Widely cultivated in Madagascar but contrary to statements in Keraudren (1966) does not seem to occur in the wild.

**Uses.** Vegetables, and young fruits are used for salad but rather rare in Madagascar.

**Taxonomy.***Cucumis sativus* is sister to *C. hystrix* (Sebastian et al. 2010).

***Cucurbita* L., Sp. Pl. 1010. 1753 (nom. cons.).**

**Generic type.***Cucurbita pepo* L.

**Worldwide Distribution.** South, Central and North America, cultivated and escaped in tropical and temperate regions worldwide (Schaefer 2020).

***Cucurbita moschata* Duchesne, Ess. Hist. Nat. Courges: 7 (1786)**

Fig. 138

**Type.** Unclear.

**Synonyms.**https://powo.science.kew.org/taxon/320034-2.

**Vernacular name.** Taboara (Masikoro, Sakalava), Voatavo (Merina).

**GenBank information.** DNA sequences of this species have been published e.g., by Zheng et al. (2013)PI653064, PI653064, PI653064, PI653064. Malagasy material has been sequenced by the authors (unpubl. data).

**Distribution.** Introduced to Madagascar probably in the 17^th^ century (Perrier de la Bâthie 1931) and today widely cultivated as a vegetable. Reported occasionally as casual, probably never growing truly wild.

**Ecology and habitat.** Cultivated at altitudes up to 1,800 m.

**Uses.***Cucurbita moschata* is a popular vegetable.

**Taxonomy.***Cucurbita moschata* is sister species to *C. argyrosperma* and split from the *C. okeechobeensis* group about 1 Ma (Castellanos-Morales et al. 2018).

**Note.**Keraudren (1966) listed *Cucurbita maxima* Duch. as cultivated species in the drier areas of Madagascar and the only species of that genus on the island. The cited herbarium material belongs to *C. moschata* and *C. maxima* has not been observed by the authors anywhere in the country.

***Cucurbita pepo* L. Sp. Pl.: 1010 (1753)**

Fig. 139

**Synonyms.**https://powo.science.kew.org/taxon/292416-1.

**Type.***Linnaeus s.n.* (Herb. LINN No. 1151.4), lectotype designated by M Keraudren-Aymonin (1975) in Aubréville & Leroy, Fl. Cambodge Laos Viêt-Nam 15: 105.) .

**GenBank information.** DNA sequences of this species (but not from Malagasy material!) published e.g., by Montero‐Pau et al. (2018): NC036638, NC036641.

**Distribution.***Cucurbita pepo* is cultivated in Madagascar but seems to be much less common than *C. moschata* and is a very rare casual.

**Ecology and habitat.** Cultivated in temperate and (sub) tropical regions around the world in a variety of soil types; more tolerant to lower temperatures than the other annual species of the genus (Whitaker 1968).

**Uses.** Cultivated in Madagascar as a vegetable.

**Taxonomy.***Cucurbita pepo* is sister to *C. argyosperma, C. moschata, C. okeechobeensis*, and *C. lundelliana* (Castellanos-Morales et al. 2018).

***Lagenaria* Ser., Mém. Soc. Phys. Genève 3: 26. 1825.**

**Generic type.***Lagenaria vulgaris* Ser.

**Worldwide Distribution.** Africa, cultivated and escaped in tropical and temperate regions worldwide (Schaefer 2020).

***Lagenaria siceraria* (Molina) Standl., Publ. Field Columb. Mus., Bot. Ser. 3: 435 (1930)**

Fig. 140

**Basionym.***Cucurbita siceraria* Molina, *Sag. Stor. Nat.* Chili 133 (1782).

**Type.** South Africa • Roggefeld, Rietpoort, 1 Jan. 1875, *A. Rehmann 3247* (Z-000004481).

**Synonyms.**https://powo.science.kew.org/taxon/134809-2.

**Vernacular name.** Tsilaniantitra (Merina).

**GenBank information.** DNA sequences of the species from outside Madagascar have been published e.g., by N’dri et al. (2016): KF982914, KF982913. Malagasy material has been sequenced by the authors (unpubl. data).

**Distribution.** Often cultivated and recorded as a casual from three provinces, including the Center, North, and South.

**Ecology and habitat.** Cultivated mainly on sandy soil, from 100–1,800 m asl.

**Uses.** The young, tender peeled fruits are eaten as vegetables, the hard, dried shells of the calabash are used as a water container or to make musical instruments.

**Taxonomy.***Lagenaria siceraria* is sister to *L. breviflora* (Schaefer et al. 2009).

***Lagenaria sphaerica* (Sond.) Naudin, Ann. Sci. Nat., Bot., sér. 5, 5: 9 (1866).**

**Basionym.***Luffa sphaerica* Sond., *Fl. Cap.* 2: 490 (1862).

**Syntypes.** South Africa • zwischen Omsamculo und Omcomas, in feuchten Niederungen und sumpfigem Thal, 1832, *J.F. Drège s.n.* HBG (HBG506395!), TUB (TUB-004706!).

**Synonyms.***Lagenaria sphaerocarpa* Arn., J. Bot. (Hooker) 3: 277 (1841); *Sphaerosicyos meyeri* Hook.f., *Fl. Trop. Afr.* 2: 532 (1871), nom. superfl.; *Sphaerosicyos sphaericus* (Sond.) Cogn., *Monogr. Phan.* 3: 466 (1881); *Lagenaria mascarena* Naudin, *Ann. Sci. Nat., Bot.*, sér. 4, 18: 187 (1862).

**GenBank information.** DNA sequences of the species (but not from Malagasy material!) published e.g., by Kress and Erickson (2007): EF590539, EF590627, EF590405, EF590779.

**Pictures.**https://powo.science.kew.org/taxon/293050-1.

**Distribution.** Widely cultivated and sometimes found as casual.

Western Domain: Diana region, Ambahatra, midstream Ambahatra on the left side, between Anketrakabe and Antafiabe, *S.-L. Stiefel 77* (P).

Sambirano Domain: Diana region, Sambirano alluvial deposits, *H. Perrier de la Bâthie 16268* (P); Nosy Be, *J.M. Hildebrandt 2961* (P), *L.H. Boivin 2128* (P); Nosy Komba, *J. Bosser 14757*, *14757bis*, *15915* (P).

Eastern Domain: Antsinanana region, Befalafa, *L. Gautier 3350* (P, WAG).

**Ecology and habitat.** Riverbanks and humid forest, mainly on clayey soil or alluvial deposits, from 400–1,000 m asl.

**Figures 137–140.**
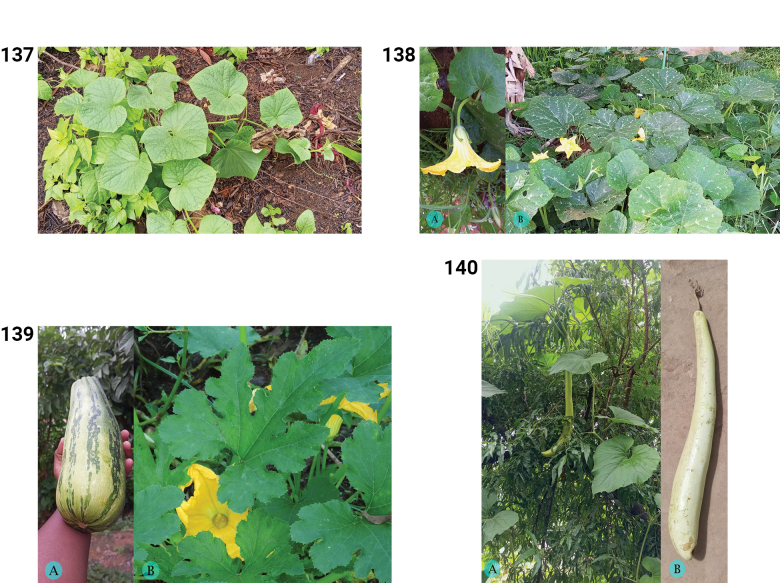
137. *Cucumis sativus*, habit (photo by MBA, Montagne des Français, January 2024). **138.***Cucurbita moschata*. **A.** Flower; **B.** Habit (photos by MBA, Joffreville, January 2024). **139.***Cucurbita pepo*. **A.** Fruit from cultivation; **B.** Habit, (photos by MBA, Antananarivo, March 2024 (**A**) and Betafo, Antsirabe, January 2020 (**B**)).**140.***Lagenaria siceraria*. **A.** Habit; **B.** Mature fruit (photos by MBA, Antananarivo, December 2023, March 2024).

**Uses.** Vegetable, the immature fruit is peeled and cooked or fried or used in curries.

***Luffa* Mill., Gard. Dict. Abridg. ed. 4. 1754.**

**Generic type.***Luffa aegyptiaca* Mill.

**Worldwide Distribution.** Tropics and Subtropics of the Old and New world (Schaefer 2020).

***Luffa acutangula* (L.) Roxb., Fl. Ind., ed. 1832. 3: 713 (1832)**

Fig. 141

**Basionym.***Cucumis acutangulus* L., *Sp. Pl.*: 1011 (1753).

**Holotype.** Philippines • Antipolo, Rizal Province, Luzon, Nov. 1914, *Merill* (Species Blancoanae No. 107), n.s.

**Synonyms.***Cucurbita acutangula* (L.) Blume, *Bijdr. Fl. Ned. Ind.*: 932 (1826).

**Vernacular name.** Papangay (Betsimisaraka, Sakalava).

**GenBank information.** DNA sequences of the species from outside Madagascar have been published e.g., by Filipowicz et al. (2014): KF487441, KF487394, KF487331, KF487330. Malagasy material has been sequenced by the authors (unpubl. data).

**Distribution.** Widely cultivated and occasionally found as casual, e.g., in the Western and Central Domains.

Western Domain: Diana region, Ankarana, *H. Schaefer & M.B. Andriamiharisoa 29, 30, 33*, *34* (TUM, DBEV); Boeny region, Mahajanga, *Poisson 98* (P); Ankarafantsika, Ampijoroa, on the forest edge, *M. Keraudren 1250* (P); Melaky region, Soalala District, Namoroka, Andranomavo township, *H. Randriamiera 7997* (P).

Central Domain: Diana region, Montagne d’Ambre, *H. Schaefer & M.B. Andriamiharisoa 43*, *43B* (January 2024).

**Ecology and habitat.** Cultivated on forest edges and in shaded areas at lower altitudes.

**Uses.** Young fruits and leaves are cooked or fried and eaten in soups and sauces.

**Taxonomy.***Luffa acutangula* is sister to all other *Luffa* species (Filipowicz et al. 2014).

***Luffa aegyptiaca* Mill., Gard. Dict., ed. 8.: [s.p.] (1768)**

Fig. 142

**Type.** Unclear.

**Synonyms.***Luffa cylindrica* auct., non (L.) Roem., Syn. Mon. 2: 63 (1846).

**Vernacular name.** Papangay (Sakalava, Merina).

**GenBank information.** DNA sequences of the species from outside Madagascar published e.g., by Filipowicz et al. (2014) and Wu et al. (2020): CM029398 JAETGN010000000, KF487456, KF487500, KF487377, KF487344, KF487450. Malagasy material has been sequenced by the authors (unpubl. data).

**Distribution.***Luffa aegyptiaca* is widely cultivated and frequently reported as casual near settlements.

**Ecology and habitat.** Cultivated and locally escaped growing on alluvial deposits at lower altitudes.

**Uses.** Young fruits and leaves eaten as vegetables.

**Taxonomy.***Luffa aegyptiaca* is sister to *L. graveolens* and *L. saccata* (Filipowicz et al. 2014).

***Sicyos* L., Sp. Pl. 2: 1013. 1753.**

**Generic type.***Sicyos angulatus* L.

**Worldwide Distribution.** South, Central and North America, Hawaii, New Zealand, Australia, invasive in Africa and Eurasia (Schaefer 2020).

***Sicyos edulis* Jacq., Enum. Syst. Pl.: 32 (1760)**

Fig. 143

**Holotype.** Jamaica • *N.J. von Jacquin* BM (BM000559376!).

**Synonyms.***Sechium edule* (Jacq.) Sw., *Fl. Ind. Occid.* 2: 1150 (1800).

**Vernacular name.** Sôsety (Merina).

**GenBank information.** DNA sequences of the species from outside Madagascar published e.g., by Kocyan et al. (2007) and Sebastian et al. (2012): DQ536727, JN560580, DQ535843, JN560209, DQ536589, DQ536861, JN560486. Malagasy material has been sequenced by the authors (unpubl. data).

**Distribution.** Widely cultivated throughout the Tropics including Madagascar and sometimes found outside cultivation but not truly naturalised on the island.

Central Domain: Analamanga region, Antananarivo, *A.C d’Alleizette, s.n.* (L), *M. Keraudren 1556* (P); Analamazaotra, *C. d’ Alleizette s. n.* (P); on the Mangoro riverbank near Antandrokomby, frequent sub-spontaneous, around the village in the warmer parts of the Central East, but not naturalised, current introduction, relatively recent introduction (during the 20^th^ century), *H. Perrier de la Bâthie 17190* (P).

Western Domain: Ankarafantsika NP, *H. Schaefer et al. 42* (TUM, DBEV); eastern Mahabo, *G. Aymonin 25961* (P).

**Ecology and habitat.** Widely cultivated in Madagascar but does not seem to occur in the wild.

**Uses.** Fruits, young leaves, shoots, and tuberous roots are commonly consumed as vegetables.

**Taxonomy.***Sicyos edulis* is sister to *Sechium chinantlense* (Sebastian et al. 2012).

***Telfairia* Hook., Bot. Mag. 2751-52. 1827.**

**Generic type.***Telfairia pedata* (Sm.) Hook.

**Worldwide Distribution.** African Tropics (Schaefer 2020).

***Telfairia pedata* (Sm.) Hook., Bot. Mag. 54: t. 2751 (1827).**

**Basionym.***Fevillea pedata* Sm., *Bot. Mag.* 53: t. 2681 (1826).

**Type.** Tanzania • Zanzibar, 1833, *W. Bojer s.n.* (K).

**Synonyms.***Joliffia africana* Delile, *Mém. Soc. Hist. Nat.* Paris 3: 314 (1827); *Telfairia africana* (Delile) A.Chev., *Rev. Int. Bot. Appl. Agric. Trop.* 29: 217 (1949).

**GenBank information.** DNA sequences of the species (but not from Malagasy material!) published e.g., by Kocyan et al. (2007): DQ374439, DQ491021, DQ501271, DQ535853.

**Pictures.**https://www.inaturalist.org/observations?taxon_id=1139409.

**Distribution.** Introduced, only known from one ancient collection from the North, no recent reports.

Sambirano Domain: Nosy-Be, cultivated, *L.H. Boivin s.n.* (P).

**Ecology and habitat.** Was probably cultivated in humid low altitude areas of Madagascar in the past but not seen more recently.

**Uses.** Seeds are eaten raw and used to produce vegetable oil on the African continent, but no such use has been documented in Madagascar.

**Taxonomy.***Telfairia pedata* is sister to *T. occidentalis* (Kocyan et al. 2007).

***Trichosanthes* L. Sp. Pl. 2: 1008. 1753.**

**Generic type.***Trichosanthes anguina* L.

**Worldwide Distribution.** Australasia (Schaefer 2020).

***Trichosanthes cucumerina* L., Sp. Pl.: 1008 (1753)**

Fig. 144

**Holotype.** Indonesia • Java, s.d., *T. Horsfield 499981A* U (U0008345).

**Isotype.** U (U0008346!).

**Vernacular name.** Mapangay (Sakalava).

**GenBank information.** DNA sequences of the species from outside Madagascar have been published e.g., by Schaefer et al. (2008) and De Boer et al. (2012): EU155603, EU155609, HE661486, HF586722, HF586731, HF586766, HF586768. Malagasy material has been sequenced by the authors (unpubl. data).

**Distribution.** Introduced to Madagascar and cultivated as a vegetable and rarely found as a casual.

**Ecology and habitat.** Cultivated at low altitudes.

**Uses.** Fruits and leaves are consumed as vegetables.

**Taxonomy.***Trichosanthes cucumerina* is sister to *T. nervifolia* (De Boer et al. 2012).

### ﻿﻿Doubtful species and erroneous records

***Cucumis africanus* L. fil., Suppl. Pl.: 423 (1782).**

**Lectotype.** France • Jardin d’expérience de Collioure, de l’intérieur de l’Afrique, 1874, *C. Naudin s.n*. P (P00346219!), designated by Kirkbride, Biosyst. Monogr. Cucumis 31 (1993).

**Distribution.** Native to the African continent (Angola, Botswana, South Africa, Namibia, and Zimbabwe (POWO 2023), based on our herbarium studies, all reports from Madagascar seem to be in error for *C. anguria*.

***Cucumis cinereus* (Cogn.) Ghebret. & Thulin, Novon 17: 177 (2007).**

**Basionym.***Kedrostis cinerea* Cogn., *Bull. Herb. Boissier*, sér. 2, 1: 883 (1901).

**Holotype**. Namibia • Hereroland, An der Giftkopje, 12^th^ Feb., 1900, *M.K. Dinter 1440* Z (Z000004457!).

**Isotype.** BR (BR0000008888297!).

**Synonyms.***Cucumella cinerea* (Cogn.) C.Jeffrey, *Kew Bull*. (1962); *Melothria cinerea* (Cogn.) A. Meeuse, *Bothalia* 8: 17 (1962).

**Distribution.** Native to Namibia, Angola, Tanzania, Kenya (POWO 2023) and has been reported from Madagascar in error.

**Taxonomy.** Genetic analyses show that Malagasy material identified as this species in fact belongs to a different undescribed species.

***Cucumis hirsutus* Sond., W.H. Harvey Cap. 2: 497 (1862).**

**Holotype**. Zimbabwe • Marandellas, 7 Jan. 1942, *G. Dehn* 582 m (M-

0105801! / 27188! / 18229!).

**Distribution.** Native to continental Africa (including Angola, Botswana, Burundi, Cameroon, Cape Provinces, Congo, Kenya, KwaZulu-Natal, Malawi, Mozambique, Northern Provinces, Sudan, Swaziland, Tanzania, Zambia, Zaïre, Zimbabwe) (POWO 2023) and has been reported from Madagascar in error.

**Figures 141–144.**
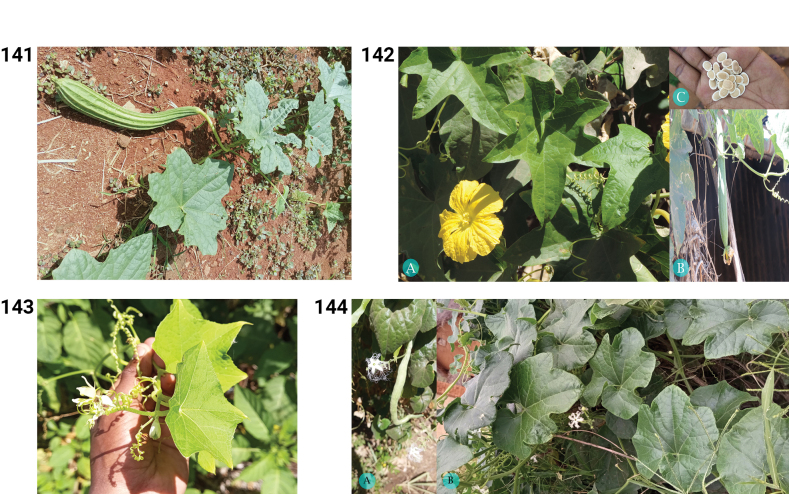
141. *Luffa acutangula*, habit (photo by MBA, Ankazomiriotra, March 2023). **142.***Luffa aegyptiaca*. **A.** Female flower; **B.** Young fruit; **C.** Seeds (photos by MBA, Marovoay, March 2020). **143.***Sicyos edulis*, cultivated plant with flowers and young fruit (photo by MBA, Ranomafana village, October 2022). **144.***Trichosanthes cucumerina*. **A.** Young fruit; **B.** Habit (photos by MBA, Andringitra, March 2023).

**Taxonomy.** Genetic analyses show that Malagasy material identified as this species in fact belongs to a different undescribed species.

***Momordica trifoliolata* Hook.f., Fl. Trop. Afr. 2: 537 (1871).**

**Holotype.** Tanzania • Morogoro Dist., Ngomero, *Grant s.n.* K (K000310605!).

**GenBank information.** DNA sequences of the species (but not from Malagasy material!) published e.g., by Schaefer and Renner (2010): GQ162917, GQ162962, GQ163454, GQ163091, GQ163214, GQ163336.

**Distribution.** Only known from a single collection in Antananarivo, Mandraka, near Mandraka forest, *C. d’ Alleizette 1079* (P). It is doubtful if this specimen really came from Madagascar.

**Taxonomy.***Momordica trifoliolata* is sister to *M. rostrata* (Schaefer and Renner 2010).

***Zehneria maysorensis* (Wight & Arn.) Arn., J. Bot. (Hooker) 3: 275 (1841).**

**Basionym.***Bryonia maysorensis* Wight & Arn., *Prodr. Fl. Ind. Orient.* 1: 345 (1834)

**Vernacular name.** Vahifamakiela (Betsileo).

**Lectotype.** India • Peninsula Ind. Orientalis [Chovei], *E.P. Wright 1116* P (P00218566!).

**GenBank information.** DNA sequences of the species (but not from Malagasy material!) published e.g., by Dwivedi et al. (2018): KY523335, KY523256, KY523425, KY523368.

**Distribution.***Zehneria maysorensis* is native to the Indian Subcontinent and has also been reported from Madagascar (POWO 2023). However, it is unlikely that the Malagasy material belongs to the same taxon found in India.

The species has been reported from the Central Domain: Haute-Matsiatra region, Ambalavao District, Mahazony township, Ankidontany, *C. Rakotovao 9173* (P), and s. loc., *P. Commerson s.n* (BR).

***Zehneria thwaithesii* (Schweinf.) C. Jeffrey, Kew Bull. 15: 371 (1961, publ. 1962).**

**Basionym.***Melothria thwaitesii* Schweinf., Reliq. Kotschy.: 44 (1868)

**Lectotype.** Sri Lanka • *Walker 275* K (K000742779), designated by C Jeffrey, Kew Bulletin 15(3): 371(1962).

**GenBank information.** DNA sequences of the species (but not from Malagasy material!) published by Dwivedi et al. (2018): AM981145, KY523315, KY523349, KY523412, KY523465.

**Distribution.** Tropical Africa, southern India, and Sri Lanka (De Wilde and Duyfjes 2006).

**Note.** Not present in Madagascar, where it has been confused with *Z. tridactyla.*

## ﻿﻿Results

### ﻿﻿Total species and comparison with the lists of Keraudren and Rauh

Following Keraudren’s study of the Cucurbitaceae family in Madagascar, which resulted in the publication of the respective volume of the Flore de Madagascar et des Comores (Keraudren 1966), very few studies of the Cucurbitaceae have been conducted, with the notable exception of Rauh’s work on the succulent taxa (1995). Monique Keraudren (1966) listed 27 genera, eight of them endemic, 66 species, and nine varieties, including six for the genus *Peponium* and three for *Zehneria*. In our revision, we accept 26 genera, five of which are endemic to Madagascar, comprising 82 taxonomic units. This total includes 77 species and five varieties: three for the genus *Peponium* and two for *Zehneria*. Among the additions in our list are new species described or reported by Keraudren after the publication of her flora volume: *Seyrigia marnieri* (Keraudren 1967), *Odosicyos bosseri* (Keraudren-Aymonin 1980, 1982a), *Diplocyclos palmatus* (Keraudren-Aymonin 1982b), and *Seyrigia napifera*, which has been described by Rauh (1995). Due to modern phylogenetic analyses, genus circumscriptions changed for many of the Malagasy cucurbits, leading to numerous name changes mainly at the genus level: *Cucumella* and *Oreosyce* were included in *Cucumis*, *Odosicyos* and *Tricyclandra* were included in *Ampelosycios*, *Sechium* was moved to *Sicyos*, *Zombitsia* moved to *Blastania*, and *Zygosicyos* included in *Xerosicyos* (Kocyan et al. 2007; Schaefer 2007; Schaefer and Renner 2011; Sebastian et al. 2012; Lindner et al. 2017). Most or all records of *Cucumis africanus*, *Cucumis sativus*, *Cucurbita maxima*, and *Citrullus colocynthis* by Keraudren (1966) seem to have been in error for *Cucumis anguria, Cucumis sacleuxii*, *Cucurbita moschata*, and *Citrullus mucosospermus*. *Cucumis dipsaceus* and *Coccinia grandis* seem to be recently introduced invaders that arrived in Madagascar after the 1980s.

Despite considerable efforts, we were unable to locate living plants of 24 taxa. Five species remain doubtful because they have most likely been confused with species occurring in India or Africa: *Cucumis cinereus, Cucumis hirsutus, Momordica trifoliolata, Zehneria thwaitesii*, and *Z. maysorensis.* A total of five new species were identified in this study, two for the genus *Ampelosycios*, two for *Cucumis*, and one for *Xerosicyos*. In addition, five species from continental Africa and India are newly reported from Madagascar: *Citrullus mucosospermus, Cucumis anguria, Coccinia grandis, Gerrardanthus grandiflorus*, and *Zehneria tridactyla*.

### ﻿﻿Total species with DNA sequences

For the vast majority of the 82 Cucurbitaceae species and varieties, DNA sequence data from Madagascar is already available on GenBank (https://www.ncbi.nlm.nih.gov/) or has been collected during our project and will be made available publicly in the near future. The genera, where more fieldwork and collection are needed most are *Cayaponia*, *Cyclantheropsis, Peponium*, and *Kedrostis*, especially those taxa known only from the types in the P herbarium.

### ﻿﻿Endemism

**Figure 145.**
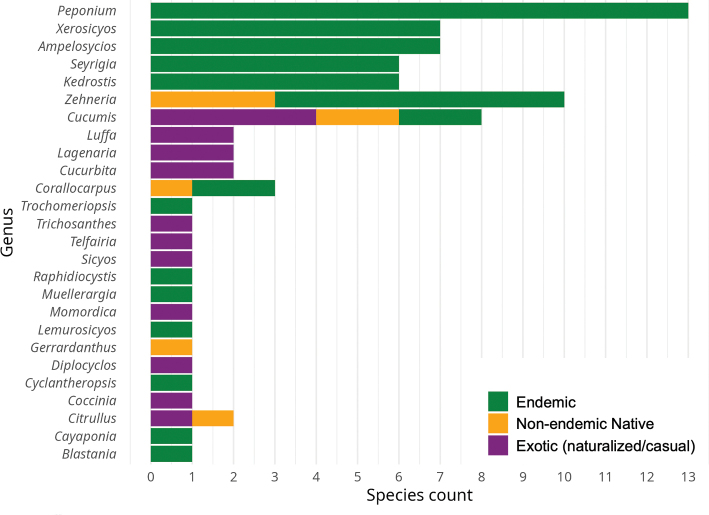
Status of Malagasy Cucurbitaceae by genus.

Regarding the distribution status of the Cucurbitaceae family in Madagascar, we classify the species in our checklist as follows: 60 are native, representing 80% of the total diversity. Endemism level is high with 86% (52 species plus 5 varieties), eight species are probably native but not endemic (Fig. 145).

We find that 17 species have been introduced, eleven of them are found in cultivation and rarely as casuals near settlements, while six exotic species have established permanent populations outside cultivated areas and some even start to become invasive in natural habitats (e.g., *Cucumis dipsaceus*).

### ﻿﻿Diversity

**Figure 146.**
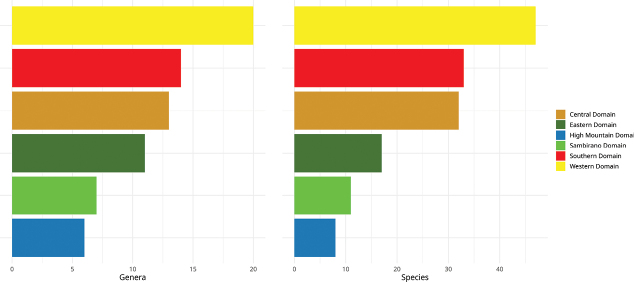
Number of Cucurbitaceae genera and species per phytogeographic domain.

The diversity analysis conducted across Madagascar’s five phytogeographic divisions (Humbert 1955) indicates that both at species and genus levels, Western, Southern and Central domains are most diverse (Fig. 146). Most of the native species, (47, 68%) occur in the Western domain, 33 (48%) in the Southern, and 32 (46%) in the Central domain. Only 17 species (25%) have been found in the Eastern domain, 11 (16%) in the Sambirano, and eight (12%) in the High Mountain Domain.

### ﻿﻿Analysis of IUCN classifications

**Figure 147.**
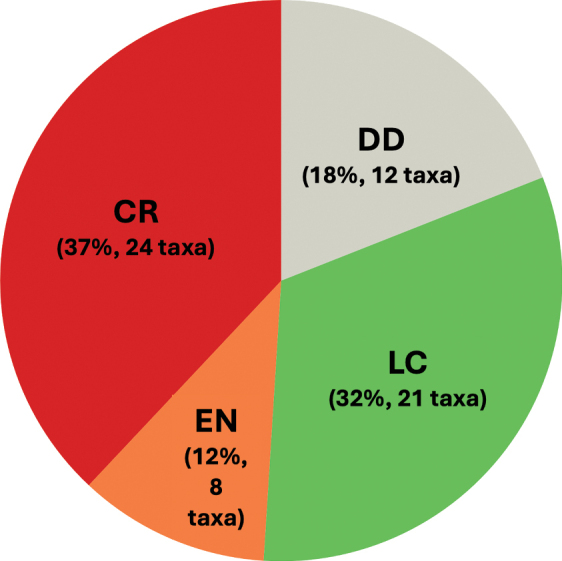
Distribution of Red list categories of Malagasy Cucurbitaceae based on IUCN Criterion B1, B2, and author’s attribution.

Our preliminary analysis of extinction risks of Malagasy wild Cucurbitaceae based on IUCN criteria B1 and B2 (Table 4), revealed that the available information for 12 taxa is insufficient, so that we suggest classifying them as Data Deficient (DD), even though some of them might have become extinct already. For 24 taxa, we suggest classification as Critically Endangered (CR), and for eight taxa, the category Endangered (EN) seems most appropriate. The remaining 21 are classified Least Concern (LC) (Fig. 147).

**Table 4.** Estimated Area of Occupancy, Extent of Occurrence, and suggested IUCN classification of the native Cucurbitaceae of Madagascar.

Scientific name	AOO (km2)	EOO (km2)	AOO Category	EOO Category	Final Assessment
*Ampelosycios bosseri*	7	121.4	CR	EN	CR
*Ampelosycios humblotii*	10	252074.8	EN	LC	LC
*Ampelosycios leandrii*	7	29229.8	CR	LC	CR
*Ampelosycios meridionalis*	3	13375.6	CR	VU	DD
*Ampelosycios scandens*	10	31560.9	EN	LC	LC
*Ampelosycios* sp. nov. Ankarana	7	163.3	CR	EN	EN
*Ampelosycios* sp. nov. Montagne des Français				EN	EN
*Blastania lucorum*	6	8574.5	CR	VU	CR
Cayaponia africana var. madagascariensis					DD
*Citrullus mucosospermus*	20	252769.8	EN	LC	LC
*Corallocarpus grevei*	29	230860	EN	LC	LC
*Corallocarpus perrieri*	22	40750.2	EN	LC	LC
*Corallocarpus poissonii*	3	1957.8	CR	EN	EN
*Cucumis sacleuxii*				LC	LC
*Cucumis* sp. nov. (aff. cinereus)	2	0	CR	CR	CR
*Cucumis* sp. nov. (aff. hirsutus)	2	0	CR	CR	CR
*Cucumis subsericeus*	27	123453.3	EN	LC	LC
*Cyclantheropsis madagascariensis*	5	233722.4	CR	LC	CR
*Gerrardanthus grandiflorus*	2	0	CR	CR	CR
*Kedrostis cogniauxii*					DD
*Kedrostis dissecta*	3	51910.4	CR	LC	CR
*Kedrostis elongata*	20	230798.4	EN	LC	LC
*Kedrostis lanuginosa*	3	40348.2	CR	LC	CR
*Kedrostis laxa*	11	8340.4	EN	VU	EN
*Kedrostis perrieri*	2	0	CR	CR	CR
*Lemurosicyos variegata*	36	376031.6	EN	LC	LC
*Muellerargia jeffreyana*	12	185339.2	EN	LC	EN
*Peponium betsiliense*	6	116897.7	CR	LC	CR
*Peponium boivinii*	3	709.5	CR	EN	CR
*Peponium grandidieri*	8	230445.2	CR	LC	LC
Peponium hirtellum var. hirtellum	3	198.3	CR	EN	CR
Peponium hirtellum var. longiracemosum	4	20	CR	CR	CR
*Peponium humbertii*	4	697.6	CR	EN	CR
*Peponium laceratum*					DD
Peponium perrieri var. glabrescens					DD
Peponium perrieri var. perrieri	6	198370.2	CR	LC	CR
*Peponium poissonii*	5	30988.5	CR	LC	CR
*Peponium racemosum*	4	26850.8	CR	LC	CR
Peponium seyrigii var. linearlobum					DD
Peponium seyrigii var. seyrigii					DD
*Raphidiocystis brachypoda*	30	126767.9	EN	LC	LC
*Seyrigia bosseri*	4	213.9	CR	EN	CR
*Seyrigia gracilis*	28	56717.7	EN	LC	LC
*Seyrigia humbertii*	5	187.2	CR	EN	EN
*Seyrigia marnieri*	2	0	CR	CR	DD
*Seyrigia multiflora*	17	44153.6	EN	LC	LC
*Seyrigia napifera*	2	0	CR	CR	CR
*Trochomeriopsis diversifolia*	61	460246.7	EN	LC	LC
*Xerosicyos danguyi*	65	102590.5	EN	LC	LC
*Xerosicyos decaryi*	16	147808.8	EN	LC	LC
*Xerosicyos hirtellus*					CR
*Xerosicyos perrieri*	33	104911.3	EN	LC	LC
*Xerosicyos pubescens*	3	36.2	CR	CR	CR
*Xerosicyos* sp. nov. North	2	0	CR	CR	CR
*Xerosicyos tripartitus*	4	901.8	CR	EN	EN
*Zehneria emirnensis*	33	262186.7	EN	LC	LC
*Zehneria madagascariensis*	2	0	CR	CR	CR
*Zehneria martinez-crovettoi*	2	0	CR	CR	DD
*Zehneria peneyana*	2	0	CR	CR	DD
Zehneria perrieri var. parvula					DD
Zehneria perrieri var. perrieri	14	56130.8	EN	LC	LC
Zehneria perrieri var. tsaratananensis					DD
*Zehneria polycarpa*	23	186792.3	EN	LC	LC
*Zehneria rutenbergiana*	5	2830.3	CR	EN	EN
*Zehneria tridactyla*	9	233017.3	CR	LC	LC

Nine of the native species are not known from any protected areas: *Ampelosicyos meridionalis*, Cayaponia africana var. madagascariensis, *Kedrostis lanuginosa*, *Peponium perrieri* (both varieties), *P. seyrigi* (both varieties), *Seyrigia marnieri*, *Xerosicyos hirtellus*, *Zehneria martinez-crovettoi*, and *Z. peneyana* (Suppl. material 1). They appear to be especially vulnerable, and their habitats should be considered for future reserve plans. About one third of the native species (21) is known only from a single of the 45 protected areas with cucurbit data, which highlights the importance of every single reserve. The maximum number of cucurbit species known from a protected area is 13, which is found in the Andohahela NP, while 12 species have been found in Zombitsy-Vohibasia NP. In each of these two parks, three cucurbit species were found that are not known from any other protected area. These two reserves are therefore crucial for protection of Cucurbitaceae diversity in Madagascar. For 52 protected areas in Madagascar, we have no occurrence data at all for native cucurbit species.

## ﻿﻿Discussion and conclusions

Our study provides new insights into the diversity of the Cucurbitaceae in Madagascar. We accept 26 genera, 77 species, and 5 varieties. We add ten species compared to the treatment of Keraudren (1966) and the species described by Keraudren and Rauh in the following years, while we delete three species and classify five as doubtful. Five species are new to science and will be described when more complete morphological, molecular, and ecological data are available. These changes can be explained by the advancements brought by molecular identification techniques and fieldwork in remote regions. Molecular approaches are essential for rapid identification and the development of conservation strategies for threatened plants and animals in Madagascar. Recent molecular revisions of various Malagasy plant and animal groups, such as the Madagascar Olive (*Norhonia*, Oleaceae) (Hong-Wa and Besnard 2014) and the Mouse Lemurs (Yoder and Heckman 2006) revealed numerous overlooked species and emphasize the need for broad sampling and comprehensive analyses of molecular, but also morphological and bioclimatic data. Such analyses are essential for an accurate assessment of species boundaries and conservation of these species.

We find the highest diversity of Cucurbitaceae in the Western phytogeographic domain, followed by the Southern, the Eastern, Sambirano, and the High Mountain domain. There is no specific centre of diversity, cucurbit lineages seem to have diversified in all major vegetation zones of the island. The high diversity of Cucurbitaceae in the Western and the Southern phytogeographic domain can be explained probably by the high temperature and low precipitation in these regions, leading to neo-endemism as described by Said Gutiérrez-Ortega et al. (2018) and Omollo et al. (2024). This is probably the case for the genus *Seyrigia*, in which several species occur in sympatry in the limit of the Western and Southern phytogeographic domain. However, a more in-depth analysis of the biogeographic and evolutionary patterns is required to test such hypothesis. At species and genus level, Andohahela NP (13 species) and the Zombitsy-Vohibasia NP (12 species) are the regions with highest taxonomic richness. The high diversity in the Andohahela region can be explained by the higher altitudinal range of this area, leading to diverse microclimates that are favourable for speciation, as observed for Cophylinae frogs (Wollenberg et al. 2008). The humid forests of the Southeast and East of Madagascar had an important role as refugia and centres of recent and rapid radiations (Antonelli et al. 2022), as this zone is in the intersection between the Southern, the Central and the Eastern domain. The lack of any records of Cucurbitaceae from 52 protected areas is most likely an artefact and not biological reality. Due to poor accessibility of many areas and research focuses on other taxonomic groups, the inventory of Malagasy cucurbits is certainly far from complete and more fieldwork will most likely lead to the discovery of native cucurbit populations in the vast majority of terrestrial reserves.

Compared to India, with an area of 3.287 Mio km^2^, Madagascar, with an area of 587,041 km^2^, has a much higher endemism rate in Cucurbitaceae. Indeed, while India harbours 94 accepted species belonging to 31 genera, with 10% endemics, the endemism rate in Madagascar reaches 88%. This significant endemism rate was highlighted by Schaefer et al. (2009) who analysed the biogeographic origin of the Malagasy Cucurbitaceae. Their results show that Cucurbitaceae reached Madagascar at least 13 times, apparently always from the African mainland, and that some of these 13 lineages then underwent local radiations, resulting in the 60 native species we find today. While this supports the status of the island as a biodiversity hotspot (Myers et al. 2000), Malagasy Cucurbitaceae seem still less diverse than other plant families on the island and even with the discovery of a few more overlooked cucurbit species in the next decades, it seems unlikely that this pattern will change. Why some lineages (*Peponium*, *Kedrostis*, *Ampelosycios*) diversified in Madagascar, while others (*Muellerargia*, *Lemurosicyos*, *Cayaponia*) did not, should be analysed carefully in follow-up studies.

The IUCN Red List assessment using criterion B1, B2, and integrating field observations of the authors shows that two thirds of the species are either threatened or cannot be assessed due to limited data. In comparison to other plant groups, Cucurbitaceae seem to have a lower extinction risk than palms (Araceae) where 83% of the endemic species have been classified as threatened (Rakotoarinivo et al. 2014) and *Pandanus* (Pandanaceae) where 91% of the species are assessed as threatened (Callmander et al. 2007). In a global assessment, however, about 36% of the plant species have been classified as threatened in their native ranges (Nic Lughadha et al. 2020), so Madagascar’s cucurbits therefore appear to be at a significantly higher risk than the global average. The high number of taxa classified as data deficient indicates that more field work is urgently needed to properly assess the threat levels and clarify the status of taxa that have not been seen for decades. The impacts of climate change with prolonged drought events and associated wildfires make the dry habitats in Madagascar with their highly specialized succulent Cucurbitaceae especially vulnerable (Ingram and Dawson 2005). In addition, illegal collection affecting more than 87% of Madagascar’s plants (Ralimanana et al. 2022), is a problem for the enigmatic succulent species, including *Xerosicyos tripartitus* and *X. pubescens*.

In summary, our results provide a solid basis for in-depth research on the native Cucurbitaceae of Madagascar, which are still poorly understood. Specifically, the ecology of most species, including their pollinators, seed dispersers, and their ethnobotanical value is poorly known. More fieldwork, including molecular identification and emerging techniques like eDNA analysis of pollinators and seed dispersers, is urgently needed to better understand and protect the wild species of this important plant family in Madagascar. Since many of them are close relatives of important crop species, their study and protection are not only a conservation issue but could have real economic benefit when we have to deal with breeding of drought-tolerant cucurbit crops in the future.

## ﻿﻿Additional information

### Use of AI

No use of AI was reported.

### Author ORCIDs

Minosoa B. Andriamiharisoa https://orcid.org/0009-0005-6237-976X

Edgardo M. Ortiz https://orcid.org/0000-0001-8052-1671

Tendro Radanielina https://orcid.org/0009-0000-4776-8605

Hanno Schaefer https://orcid.org/0000-0001-7231-3987

### Data availability

All of the data that support the findings of this study are available in the main text or Supplementary Information.

## Supplementary materials

﻿Supplementary material 1 Occurrences in protected areas Data type xlsx https://binary.pensoft.net/file/1485396This dataset is made available under the Open Database License (http://opendatacommons.org/licenses/odbl/1.0/). The Open Database License (ODbL) is a license agreement intended to allow users to freely share, modify, and use this Dataset while maintaining this same freedom for others, provided that the original source and author(s) are credited.Minosoa B. Andriamiharisoa, Edgardo M. Ortiz, Tendro Radanielina, Hanno Schaefer
